# Supplementary material for: C(alkyl)–C(vinyl) bond cleavage enabled by Retro-Pallada-Diels-Alder reaction
Source: Nat Commun. 2023 May 4;14:2572. doi: 10.1038/s41467-023-38067-7 (PMC10160084; doi:10.1038/s41467-023-38067-7)
Supplement: Supplementary file 1 — Supplementary Information [file 41467_2023_38067_MOESM1_ESM.pdf]

Supplementary Information

C(*alkyl*)–C(*vinyl*) Bond Cleavage Enabled by Retro-Pallada-Diels-Alder Reaction

Qingyang Zhao,[1,2,3] Le Yu,[1,3] Yao-Du Zhang,[1]† Yong-Qiang Guo,[1] Ming Chen,[1] Zhi-Hui Ren,[1] and Zheng-Hui Guan[1*]

[1] Key Laboratory of Synthetic and Nature Molecule of Ministry of Education, Department of Chemistry & Materials Science, Northwest University, Xi'an 710127, P. R. China
[2] School of Pharmaceutical Sciences (Shenzhen), Shenzhen Campus of Sun Yat-sen University, Shenzhen, 518107, P.R. China

[3] These authors contributed equally: Qingyang Zhao, Le Yu, Yao-Du Zhang

*guanzhh@nwu.edu.cn

CONTENT

1. General information. 2

2. Supplementary Methods and Discussions 2

2.1. General procedure for Pd-catalyzed C(alkyl)–C(vinyl) bond cleavage. 2

2.2. Model reaction with N-^{t}Bu 1A as the substrate 3

2.3. Proposed pathway for Pd-catalyzed reductive hydrolysis of Int-C. 3

2.4. Density functional theory (DFT) calculations. 4

2.5. Spectroscopic data for products 5

2.6. Copies of 1H and 13C NMR spectra 10

3. Supplementary References 58

1. General information.

Unless otherwise stated, all reagents and solvents were purchased from commercial suppliers (Energy Chemical and Bidepharm) and used without further purification. Reactions were monitored by analytical thin layer chromatography (TLC) or Agilent GC-MS analysis. ^{1}H and ^{13}C NMR spectra were recorded on Bruker instrument Advance 400 and referenced internally to the residual proton resonance in $CDCl_3$ (δ 7.26 ppm), or with tetramethylsilane (TMS, δ 0.00 ppm) as the internal standard. Chemical shifts (δ) were reported as part per million (ppm) in δ scale downfield from TMS. The following abbreviations (or combinations thereof) were used to explain multiplicities: s = singlet, d = doublet, t = triplet, q = quartet, m = multiplet, b = broad. Coupling constants *J* were reported in Hertz unit (Hz). Analytical TLC was performed on silica gel GF254, and visualization was carried out with UV light. Flash column chromatography was performed with Silica gel 60 (200-300 mesh).

The starting allyl ketones in the paper were prepared according to the following literature:

Y.-Q. Guo, M.-N. Zhao, Z.-H. Ren, Z.-H. Guan, *Org. Lett.* **2018**, *20*, 3337−3340.

2. Supplementary Methods and Discussions

2.1. General procedure for Pd-catalyzed C(alkyl)–C(vinyl) bond cleavage.

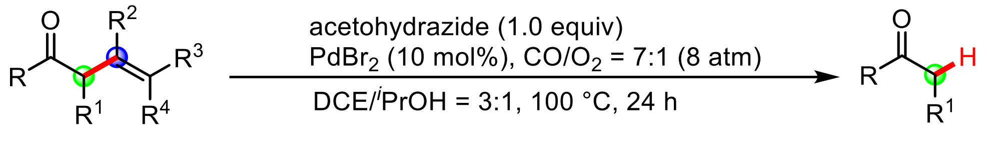

Supplementary Fig. 1. General procedure for Pd-catalyzed C(alkyl)–C(vinyl) bond cleavage.

Ketone **1a** (0.2 mmol), acetohydrazide (0.2 mmol, 14.8 mg), $PdBr_2$ (10 mol%, 5.3 mg), and 2 mL mixed solvents (DCE/^{i}PrOH = 3/1) was added to a 10 mL glass vial capped with perforated aluminum foil. The vial was then put into an autoclave and was carefully evacuated and backfilled with O_2 (1 atm) for three cycles, followed by CO (7 atm). The reaction mixture in the autoclave was stirred at a 100 °C oil bath for 24 hours. After that, the autoclave was removed from the oil bath and cooled down to room temperature prior to the release of gas carefully in a well-ventilated fume hood. The reaction mixture was quenched with H_2O (10 mL) and extracted with EtOAc (3 × 10 mL). The combined organic layers were dried over anhydrous Na_2SO_4 and

then evaporated in vacuo. The residue was purified by column chromatography on silica gel to afford the corresponding ketone **2** with hexanes/EtOAc (20/1) as the eluent.

2.2. Model reaction with *N*-^{t}Bu 1A as the substrate

(<u>the model substrate for DFT calculations</u>).

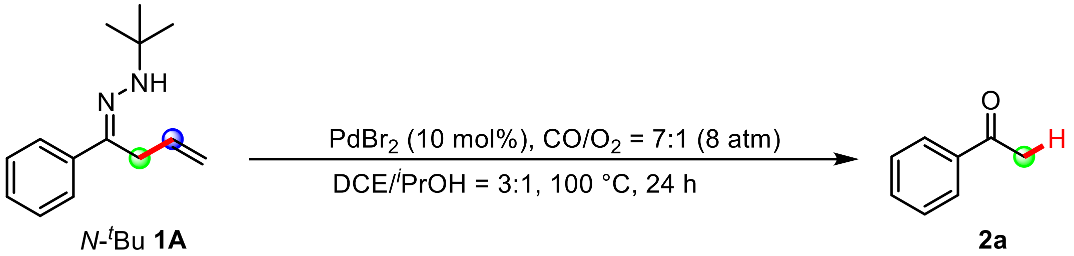

Supplementary Fig. 2. Model reaction with *N*-^{t}Bu **1A** as the substrate.

N-^{t}Bu **1A** (0.2 mmol), $PdBr_2$ (10 mol%, 5.3 mg), and 2 mL mixed solvents (DCE/^{i}PrOH = 3/1) was added to a 10 mL glass vial capped with perforated aluminum foil. The vial was then put into an autoclave and was carefully evacuated and backfilled with O_2 (1 atm) for three cycles, followed by CO (7 atm). The reaction mixture in the autoclave was stirred at a 100 ^oC oil bath for 24 hours. After that, the autoclave was removed from the oil bath and cooled down to room temperature prior to the release of gas carefully in a well-ventilated fume hood. The reaction mixture was quenched with H_2O (10 mL) and extracted with EtOAc (3 × 10 mL). The combined organic layers were dried over anhydrous Na_2SO_4 and then evaporated in vacuo. The residue was purified by column chromatography on silica gel to afford the corresponding ketone **2a** in 81% yield.

2.3. Proposed pathway for Pd-catalyzed reductive hydrolysis of Int-C.

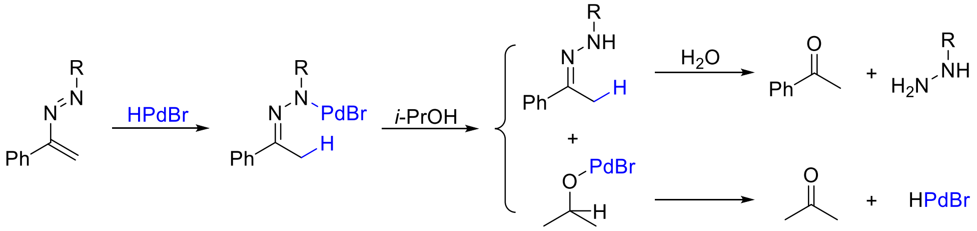

Supplementary Fig. 3. Proposed pathway for Pd-catalyzed reductive hydrolysis of **Int-C**.

2.4. Density functional theory (DFT) calculations.

Materials and Methods: All calculations were performed with the Gaussian 09 package (1). Geometry optimizations were performed with the dispersion-corrected density functional method B3LYP-D3 (2-4) with solvent effects in dichloroethane represented by the SMD solvation model (5). The 6-311++G(d,p) basis set (6-8) was used for C, H, O and N atoms and the SDD basis set (9-10) was used for Pd. Normal vibrational mode analysis at the same level of theory confirmed that the optimized structures are minima (zero imaginary frequency) or saddle points (one imaginary frequency). The relative energies and free energies (at 298.15K) with respect to **Im1** are in kcal/mol.

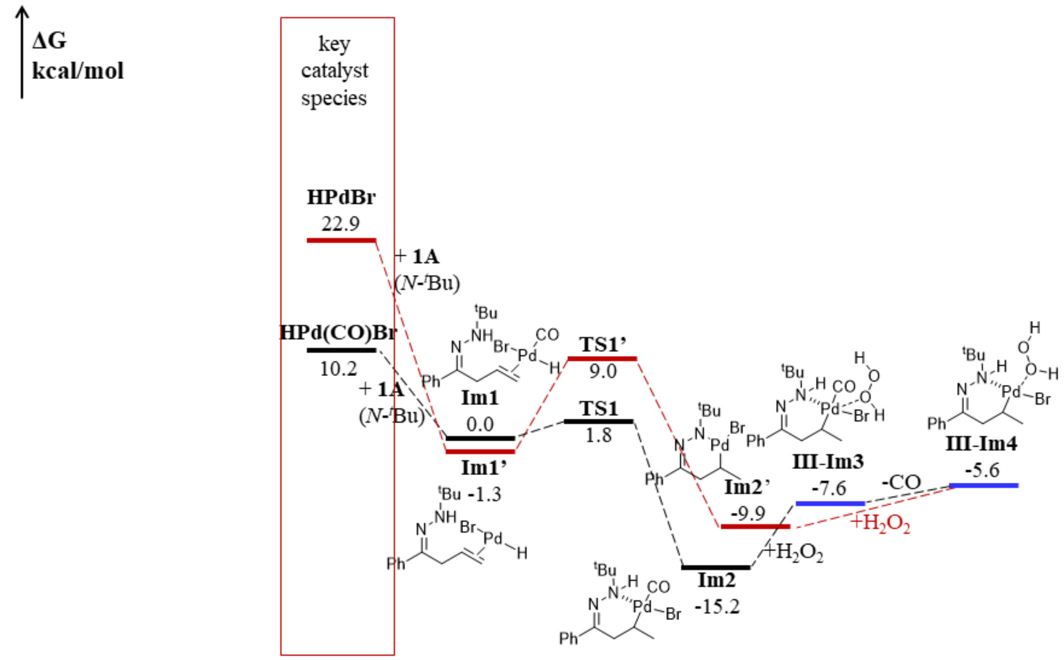

Supplementary Fig. 4. DFT calculated Gibbs free energy profiles for transformation of $PdBr_2$ to palladium hydride and insertion of palladium hydride catalyst into C=C bond, with or without CO.

2.5. Spectroscopic data for products

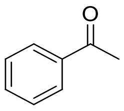

2a

2a: Yield: 82%, 19.7 mg, oil. ^{1}H NMR (400 MHz, $CDCl_3$) δ 7.96 (d, *J* = 7.2 Hz, 2H), 7.56 (t, *J* = 7.4 Hz, 1H), 7.46 (t, *J* = 7.6 Hz, 2H), 2.60 (s, 3H); ^{13}C NMR (101 MHz, $CDCl_3$) δ 198.1, 137.1, 133.0, 128.5, 128.3, 26.6.

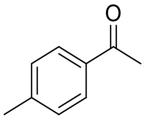

2b

2b: Yield: 82%, 22.0 mg, oil. ^{1}H NMR (400 MHz, $CDCl_3$) δ 7.86 (d, *J* = 8.3 Hz, 2H), 7.26 (d, *J* = 8.0 Hz, 2H), 2.58 (s, 3H), 2.41 (s, 3H); ^{13}C NMR (101 MHz, $CDCl_3$) δ 197.8, 143.8, 134.7, 129.2, 128.4, 26.5, 21.6.

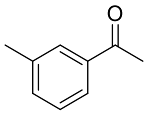

2c

2c: Yield: 76%, 20.4 mg, oil. ^{1}H NMR (400 MHz, CDCl3) δ 7.78-7.46 (m, 2H), 9.39-7.33 (m, 2H), 2.56 (s, 3H), 2.42(s, 3H); ^{13}C NMR (101 MHz, $CDCl_3$) δ 198.4, 138.3, 137.1, 133.8, 128.8, 128.4, 125.6, 26.7, 21.3.

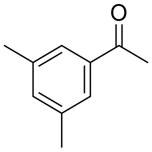

2d

2d: Yield: 83%, 24.6 mg, oil. ^{1}H NMR (400 MHz, CDCl3) δ 7.57 (s, 2H), 7.20 (s, 1H), 2.56 (s, 3H), 2.37 (s, 6H); ^{13}C NMR (101 MHz, $CDCl_3$) δ 198.6, 138.2, 137.2, 134.7, 126.1, 26.7, 21.2.

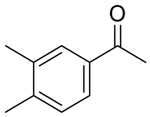

2e

2e: Yield: 83%, 24.6 mg, oil. ^{1}H NMR (400 MHz, $CDCl_3$) δ 7.73 (s, 1H), 7.69 (d, *J* = 7.8 Hz, 1H), 7.21 (d, *J* = 7.7 Hz, 1H), 2.57 (s, 3H), 2.31 (s, 6H); ^{13}CNMR (101 MHz, $CDCl_3$) δ 198.1, 142.6, 136.8, 135.1, 129.7, 129.4, 126.1, 26.5, 20.0, 19.7.

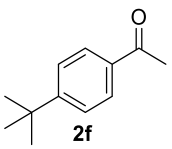

2f

2f: Yield: 72%, 25.3 mg oil. ^{1}H NMR (400 MHz, $CDCl_3$) δ 7.90 (d, *J* = 8.6 Hz, 2H), 7.48 (d, *J* = 8.6 Hz, 2H), 2.59 (s, 3H), 1.34 (s, 9H); ^{13}C NMR (101 MHz, $CDCl_3$) δ 197.9, 156.8, 134.6, 128.3, 125.5, 35.1, 31.1, 26.5.

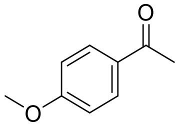

2g

2g: Yield: 80%, 24.0 mg, oil. ^{1}H NMR (400 MHz, $CDCl_3$) δ 7.94 (d, *J* = 9.0 Hz, 1H), 6.93 (d, *J* = 9.0 Hz, 1H), 3.87 (s, 2H), 2.56 (s, 2H).^{13}C NMR (101 MHz, $CDCl_3$) δ 196.7, 163.4, 130.6, 130.3, 113.6, 55.4, 26.3.

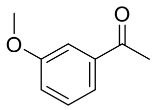

2h

2h: Yield: 76%, 22.8 mg, oil. ^{1}H NMR ($CDCl_3$, 400MHz) δ 7.53 (d, *J* = 7.9 Hz, 1H), 7.48 (s, 1H), 7.37 (t, *J* = 7.9 Hz, 1H), 7.11 (dd, *J* = 8.3, 2.7 Hz, 1H), 3.85 (s, 3H), 2.59 (s, 3H); ^{13}CNMR (101 MHz, $CDCl_3$) δ 197.8, 159.6, 138.3, 129.4, 121.0, 119.5, 112.2, 55.3, 26.6.

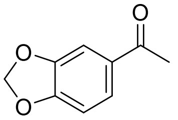

2i

2i: Yield: 69%, 22.6 mg, oil. ^{1}HNMR (400 MHz, CDCl3) δ 7.56-7.54 (dd, *J* = 8.0 Hz, 1H), 7.43 (s, 1H), 7.85 (d, *J* = 8.0 Hz, 1H), 6.05 (s, 2H), 2.54 (s, 3H); ^{13}C NMR (101 MHz, $CDCl_3$) δ 196.2, 151.7, 148.1, 132.0, 124.7, 107.9, 107.8, 101.8, 26.4.

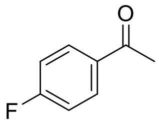

2j

2j: Yield: 73%, 20.2 mg, oil. ^{1}H NMR (400 MHz, CDCl3) δ 8.00-7.97 (m, 2H), 7.16-7.11 (m, 2H), 2.59 (s, 3H), ^{13}C NMR (101 MHz, $CDCl_3$) δ 196. 5, 165.8 (d, J_{CF} = 126.5 Hz), 133.6 (d, J_{CF} = 1.5 Hz), 130.2 (d, J_{CF} = 4.6 Hz), 115.6 (d, J_{CF} = 10.8 Hz), 26.5.

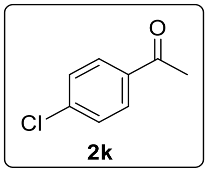

2k: Yield: 75%, 23.1 mg, oil. ^{1}H NMR (400 MHz, $CDCl_3$) δ 7.90 (d, *J* = 8.6 Hz, 2H), 7.44 (d, *J* = 8.6 Hz, 2H), 2.59 (s, 3H); ^{13}C NMR (101 MHz, $CDCl_3$) δ 196.8, 139.5, 135.4, 129.7, 128.8, 26.5.

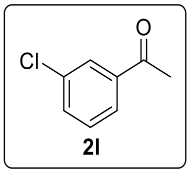

2l: Yield: 71%, 21.9 mg, oil. ^{1}HNMR (400 MHz, $CDCl_3$) δ 7.93 (s, 1H), 7.85-7.82 (m, 1H), 7.55-7.52 (m, 1H), 7.41(t, *J* = 8.0Hz, 1H), 2.60 (s, 3H); ^{13}C NMR (101 MHz, $CDCl_3$) δ 196.7, 138.5, 134.9, 133.0, 129.9, 128.4, 126.4, 26.6.

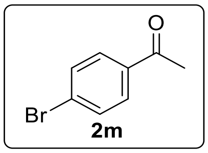

2m: Yield: 76%, 30.1 mg, oil. ^{1}H NMR (400 MHz, $CDCl_3$) δ 7.82 (d, *J* = 8.8 Hz, 2H), 7.61 (d, *J* = 8.8 Hz, 2H), 2.59 (s, 3H); ^{13}C NMR (101 MHz, $CDCl_3$) δ 197.0, 135.8, 131.9, 129.8, 128.3, 26.5.

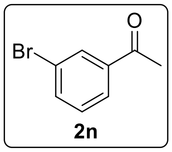

2n: Yield: 72%, 28.5 mg, oil. ^{1}H NMR (400 MHz, $CDCl_3$) δ 8.08 (s, 1H), 7.88 (d, *J* = 7.8 Hz, 1H), 7.70-7.68 (m, 1H), 7.35 (t, *J* = 7.9 Hz, 1H), 2.59 (s, 3H); ^{13}C NMR (101 MHz, $CDCl_3$) δ 196.6, 138.8, 135.9, 131.3, 130.2, 126.8, 122.9, 26.6.

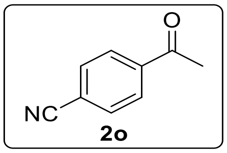

2o: Yield: 55%, 16.0 mg, oil. ^{1}H NMR (400 MHz, $CDCl_3$) δ 8.05 (d, *J* = 8.4 Hz, 2H), 7.78 (d, *J* = 8.0 Hz, 2H), 2.65 (s, 3H); ^{13}C NMR (101 MHz, $CDCl_3$) δ 196.5, 139.9, 132.5, 132.3, 128.6, 117.9, 116.3, 26.7.

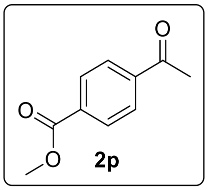

2p: Yield: 65%, 23.1 mg, oil. ^{1}H NMR (400 MHz, $CDCl_3$) δ 8.13 (d, *J* = 8.4 Hz, 2H), 8.01 (d, *J* = 7.6 Hz, 2H), 3.95 (s, 3H), 2.65 (s, 3H); ^{13}C NMR (101 MHz, $CDCl_3$) δ 197.5, 166.2, 140.2, 133.9, 129.8, 128.2, 52.4, 26.8.

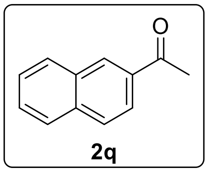

2q: Yield: 74%, 25.2 mg, oil. ^{1}H NMR (400 MHz, $CDCl_3$) δ 8.46 (s, 1H), 8.03 (dd, *J* = 8.6, 1.8 Hz, 1H), 7.96 (d, *J* = 8.0 Hz, 1H), 7.91-7.84 (m, 2H), 7.62-7.53 (m, 2H), 2.72 (s, 3H); ^{13}CNMR (101 MHz, $CDCl_3$) δ 198.1, 135.5, 134.4, 132.5, 130.2, 129.5, 128.4, 128.4, 127.7, 126.7, 123.8, 26.7.

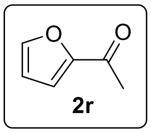

2r: Yield: 70%, 15.4 mg, oil. ^{1}H NMR (400 MHz, $CDCl_3$) δ 7.59 (dd, *J* = 2.0 Hz, 0.8 Hz, 1H), 7.19 (dd, *J* = 3.6 Hz, 0.8 Hz, 1H), 6.55- 6.54 (dd, *J* = 3.6 Hz, 2.0 Hz, 1H), 2.49 (s, 3H); ^{13}C NMR (101 MHz, $CDCl_3$) δ 186.8, 152.8, 146.4, 117.2, 112.2, 26.0.

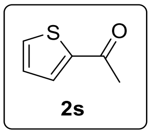

2s: Yield: 68%, 17.1 mg, oil. ^{1}H NMR (400 MHz, $CDCl_3$) δ 7.71-7.70 (dd, *J* = 4.0 Hz, 1.2 Hz, 1H), 7.65-7.63 (dd, *J* = 4.8 Hz, 1.2 Hz, 1H), 7.15-7.12 (dd, *J* = 4.8 Hz, 4.0 Hz, 1H), 2.57 (s, 3H); ^{13}C NMR (101 MHz, $CDCl_3$) δ 190.7, 144.6, 133.7, 132.4, 128.1, 26.9.

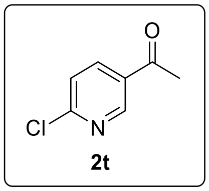

2t: Yield: 78%, 24.2 mg, colorless solid. ^{1}H NMR (600 MHz, $CDCl_3$) δ 8.94 (d, *J* = 2.4 Hz, 1H), 8.20 (dd, *J* = 8.3, 2.5 Hz, 1H), 7.45 (dd, *J* = 8.3, 0.7 Hz, 1H), 2.64 (s, 3H). ^{13}C NMR (151 MHz, $CDCl_3$) δ 195.4 , 155.7, 150.2, 138.1, 131.2, 124.6, 26.7.

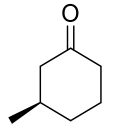

2w

2w: Yield: 45%, 10.1 mg, oil.^{1}H NMR (600 MHz, $CDCl_3$) δ 2.40–2.32 (m, 2H), 2.26–2.21 (m, 1H), 2.06–1.98 (m, 2H), 1.92–1.84 (m, 2H), 1.72–1.63 (m, 1H), 1.37–1.20 (m, 1H), 1.02 (d, *J* = 6.5 Hz, 2H). ^{13}C NMR (151 MHz, $CDCl_3$) δ 211.9, 50.0, 41.1, 34.2, 33.3, 25.3, 22.0.

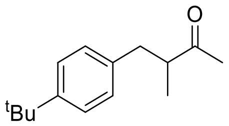

2x

2x: Yield: 79%, 34.4 mg, oil. ^1H NMR (600 MHz, $CDCl_3$) δ 7.29 (d, *J* = 8.1 Hz, 2H), 7.08 (d, *J* = 8.1 Hz, 2H), 2.97 (dd, *J* = 13.6, 6.7 Hz, 1H), 2.82 (dd, *J* = 14.2, 7.1 Hz, 1H), 2.53 (dd, *J* = 13.7, 7.9 Hz, 1H), 2.09 (s, 3H), 1.30 (s, 9H), 1.09 (d, *J* = 7.0 Hz, 3H). ^{13}C NMR (151 MHz, $CDCl_3$) δ 212.2, 149.0, 136.5, 128.6, 125.3, 48.8, 38.4, 34.4, 31.4, 28.7, 16.3.

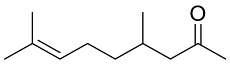

2y

2y: Yield: 68%, 22.9 mg, oil. ^1H NMR (400 MHz, $CDCl_3$) δ 5.11-5.06 (m, 1H), 2.45-2.39 (m, 1H), 2.31(d, *J* = 3.2 Hz, 1H), 2.26-2.21 (m, 1H), 2.12(s, 3H), 2.04-1.94 (m, 4H), 1.68 (s, 3H), 1.60 (s, 3H), 1.24-1.13 (m, 2H), 0.88 (d, *J* = 6.8Hz, 3H); ^{13}C NMR (101 MHz, $CDCl_3$) δ 209.1, 131.5, 124.2, 51.2, 36.9, 30.4, 29.0, 25.7, 25.4, 19.7, 17.6.

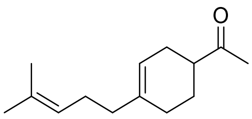

2z

2z: Yield: 52%, 21.4 mg, oil. ^1H NMR (600 MHz, $CDCl_3$) δ 5.41 (s, 1H), 5.09 (t, *J* = 6.4 Hz, 1H), 2.60-2.49 (m, 1H), 2.18 (s, 2H), 2.17 (s, 3H), 2.10-1.94 (m, 8H), 1.68 (s, 3H), 1.60 (s, 3H). ^{13}C NMR (151 MHz, $CDCl_3$) δ 211.8, 137.5, 131.5, 124.1, 118.9, 47.4, 37.5, 27.9, 27.9, 27.0, 26.3, 25.6, 24.9, 17.6.

2.6. Copies of 1H and 13C NMR spectra

Supplementary Fig. 5.^{1}H NMR of compound **2a**

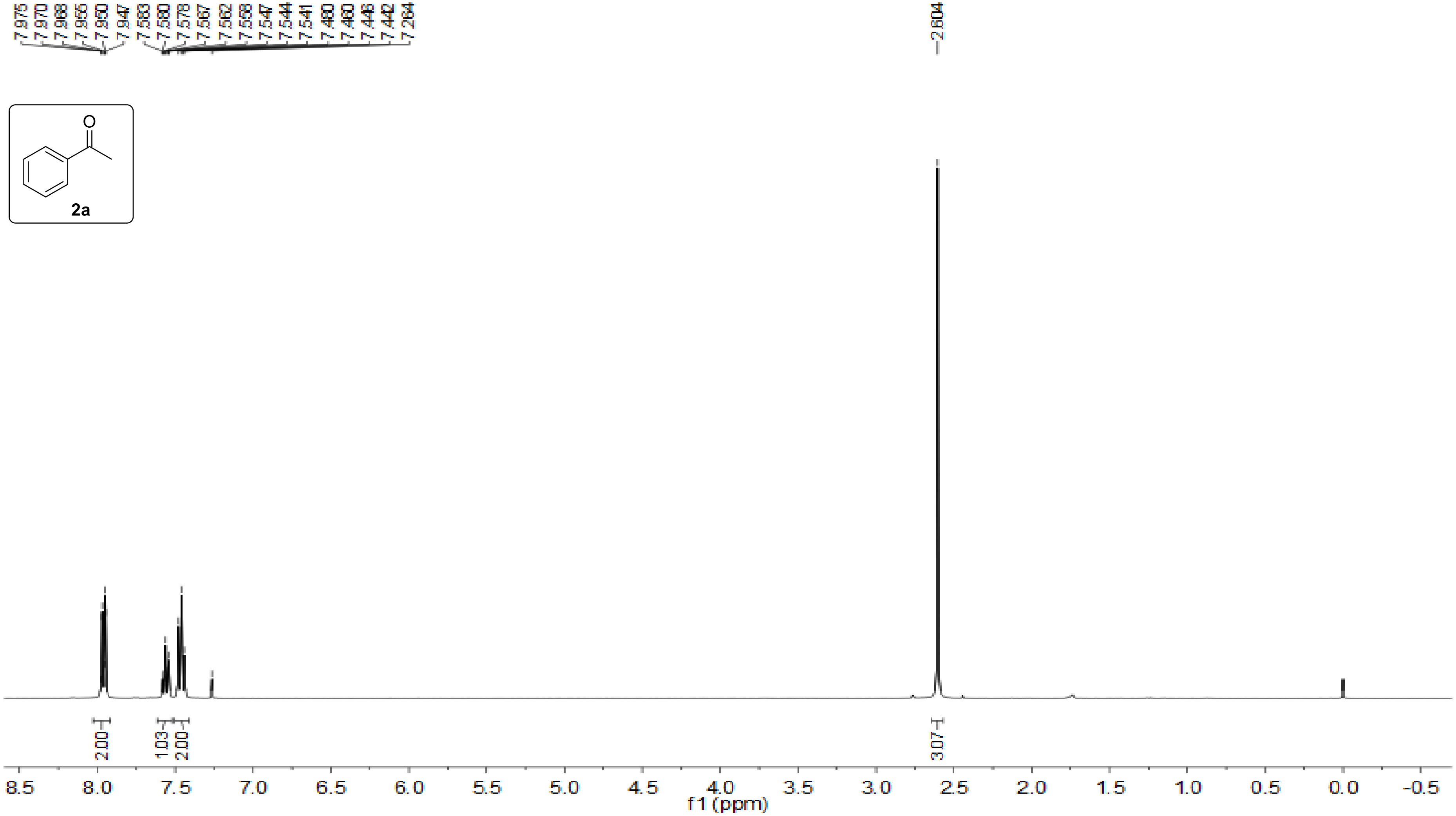

Supplementary Fig. 6. ^{13}C NMR of compound **2a**

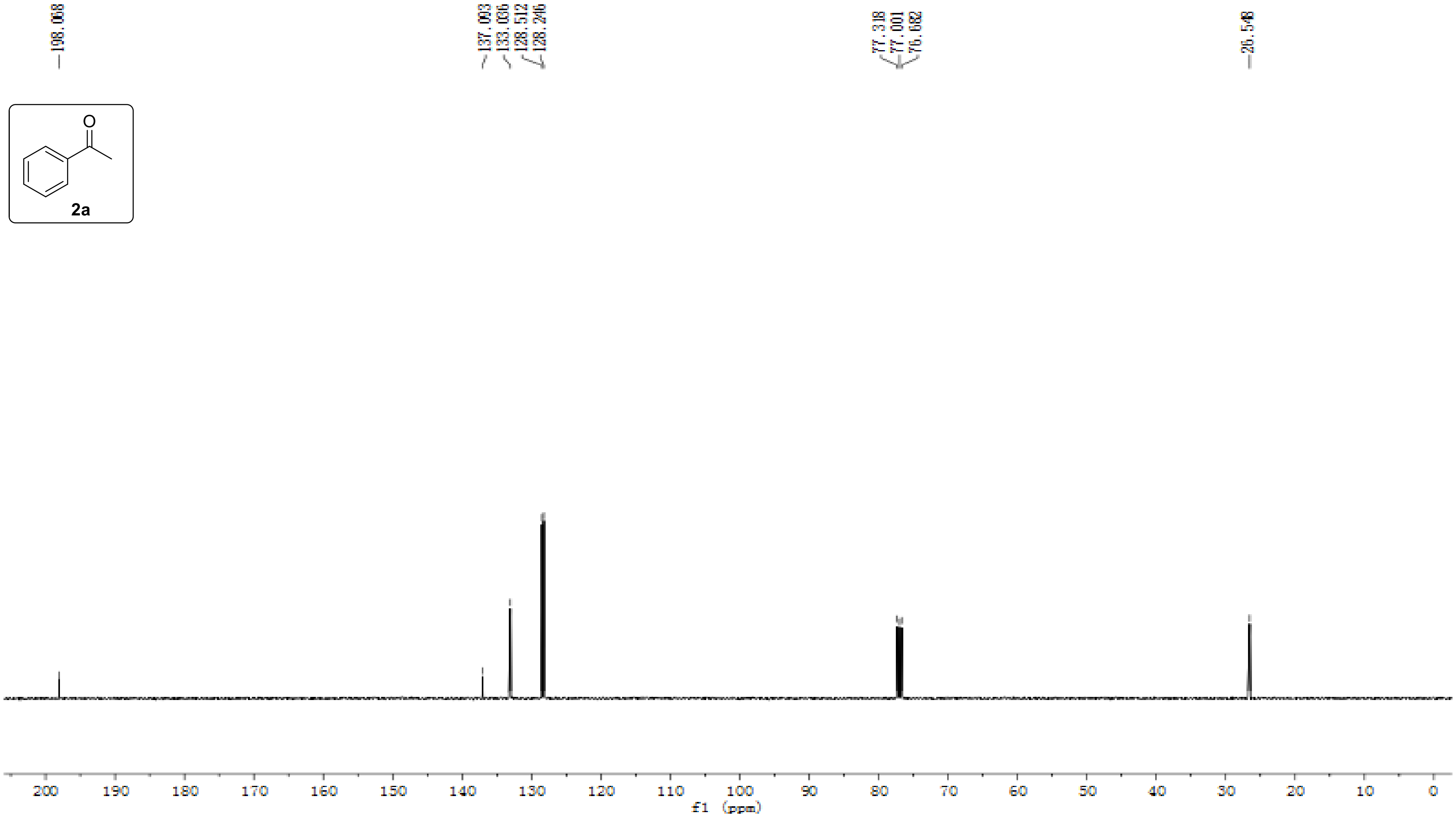

Supplementary Fig. 7.^{1}H NMR of compound **2b**

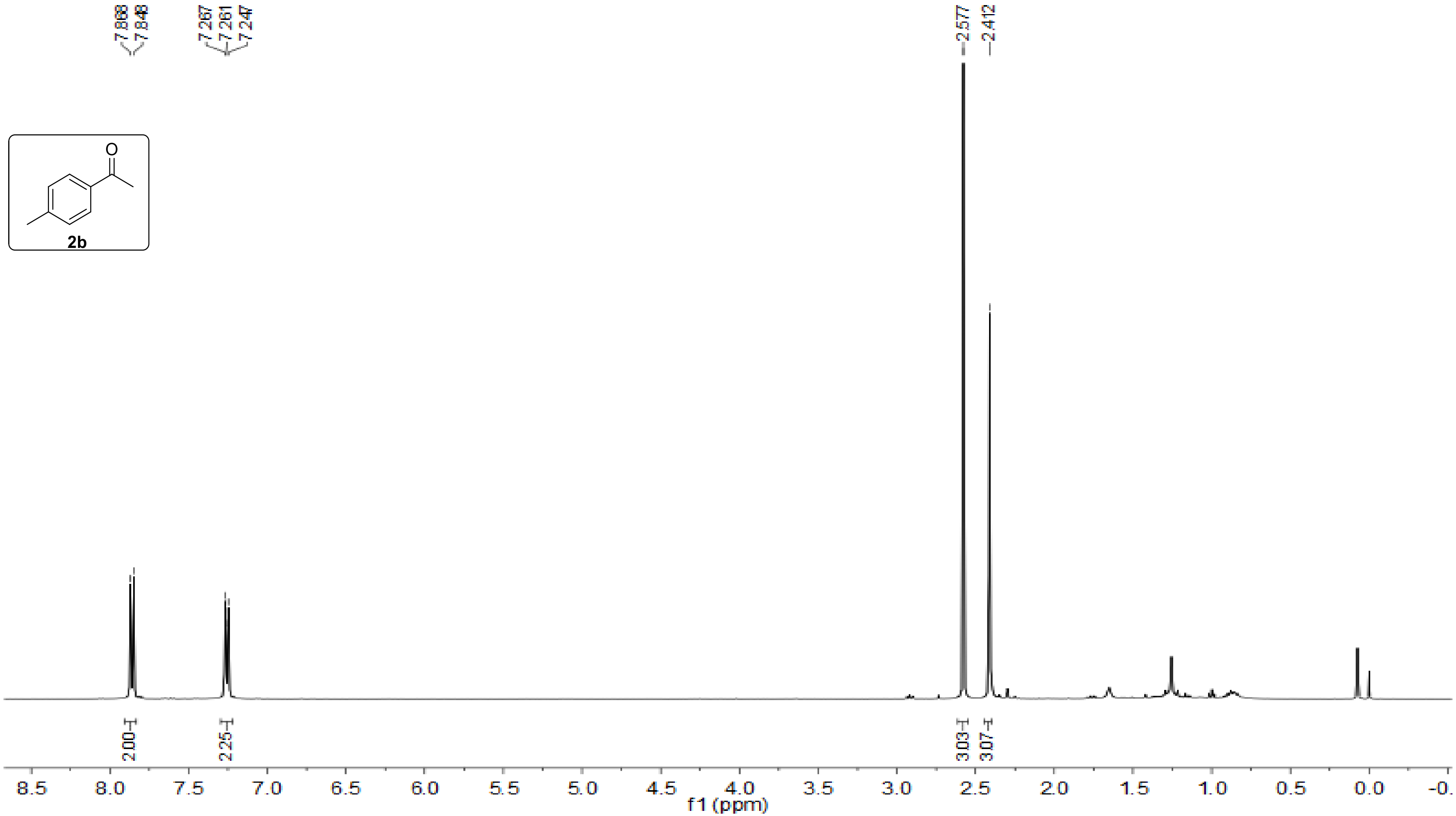

Supplementary Fig. 8. ^{13}C NMR of compound **2b**

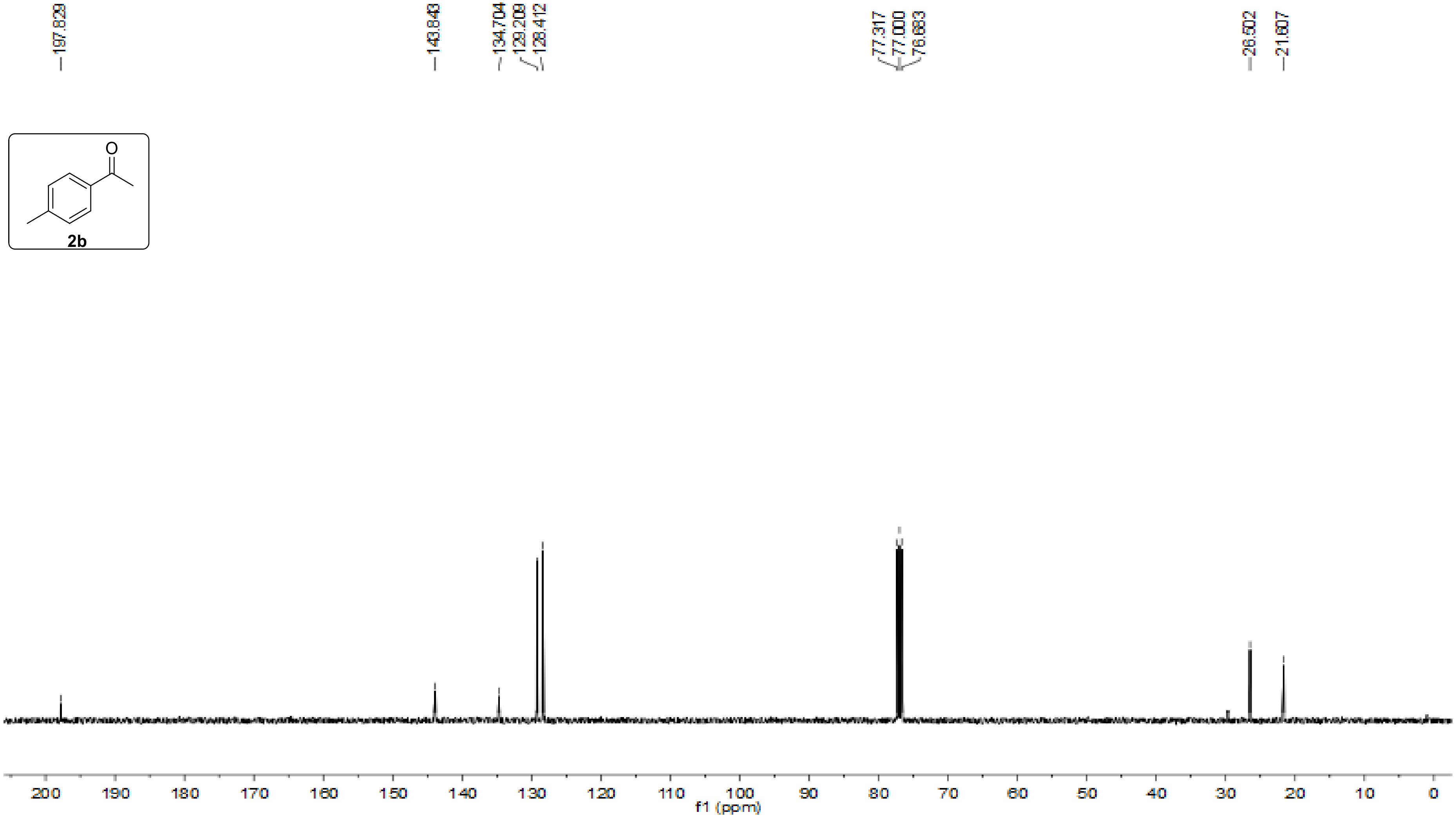

Supplementary Fig. 9.^{1}H NMR of compound **2c**

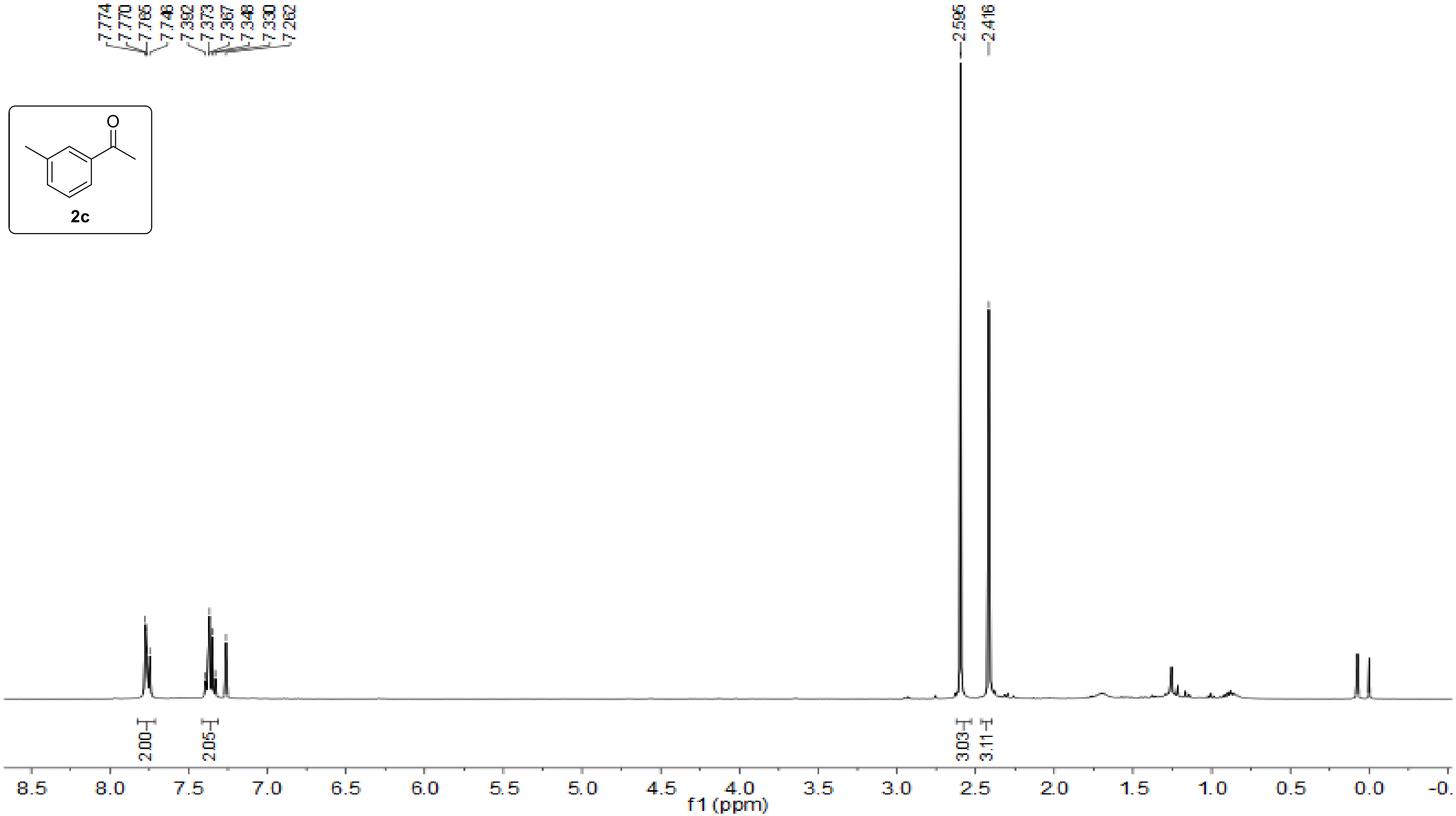

Supplementary Fig. 10. ^{13}C NMR of compound **2c**

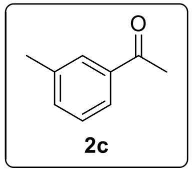

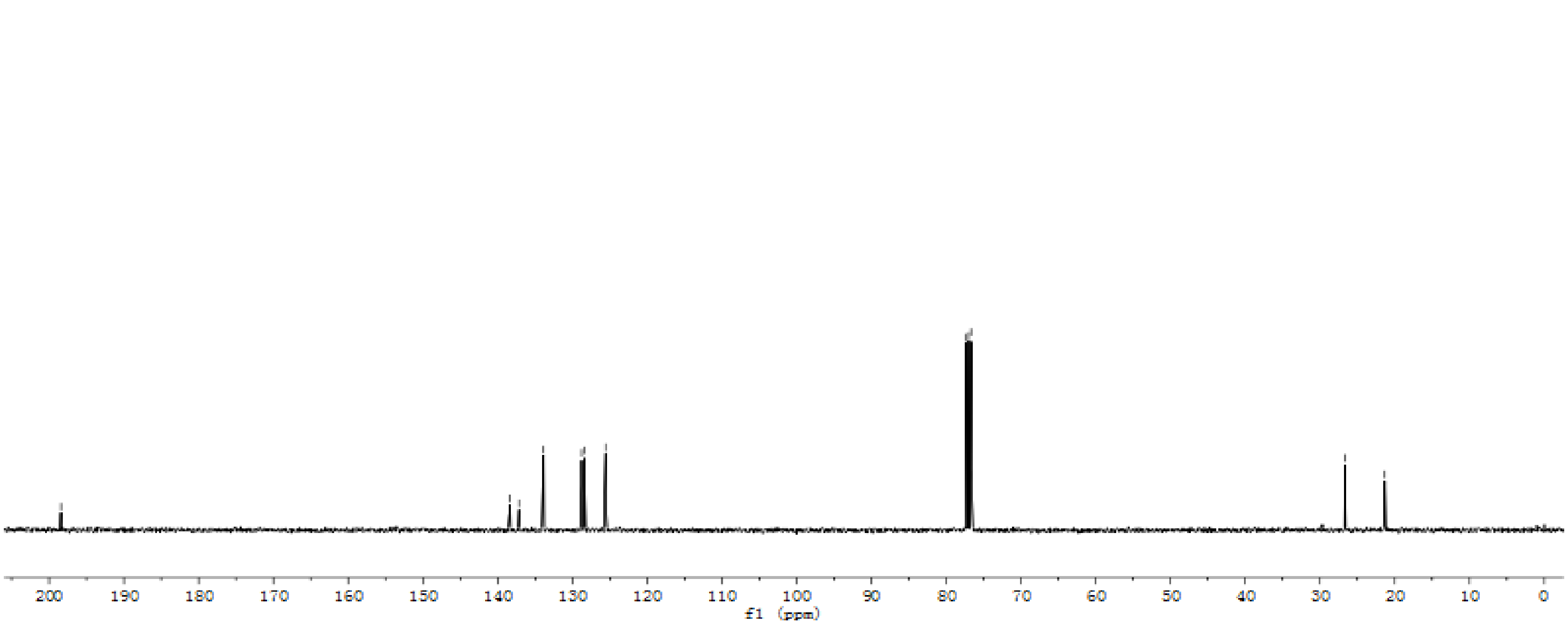

Supplementary Fig. 11.^{1}H NMR of compound **2d**

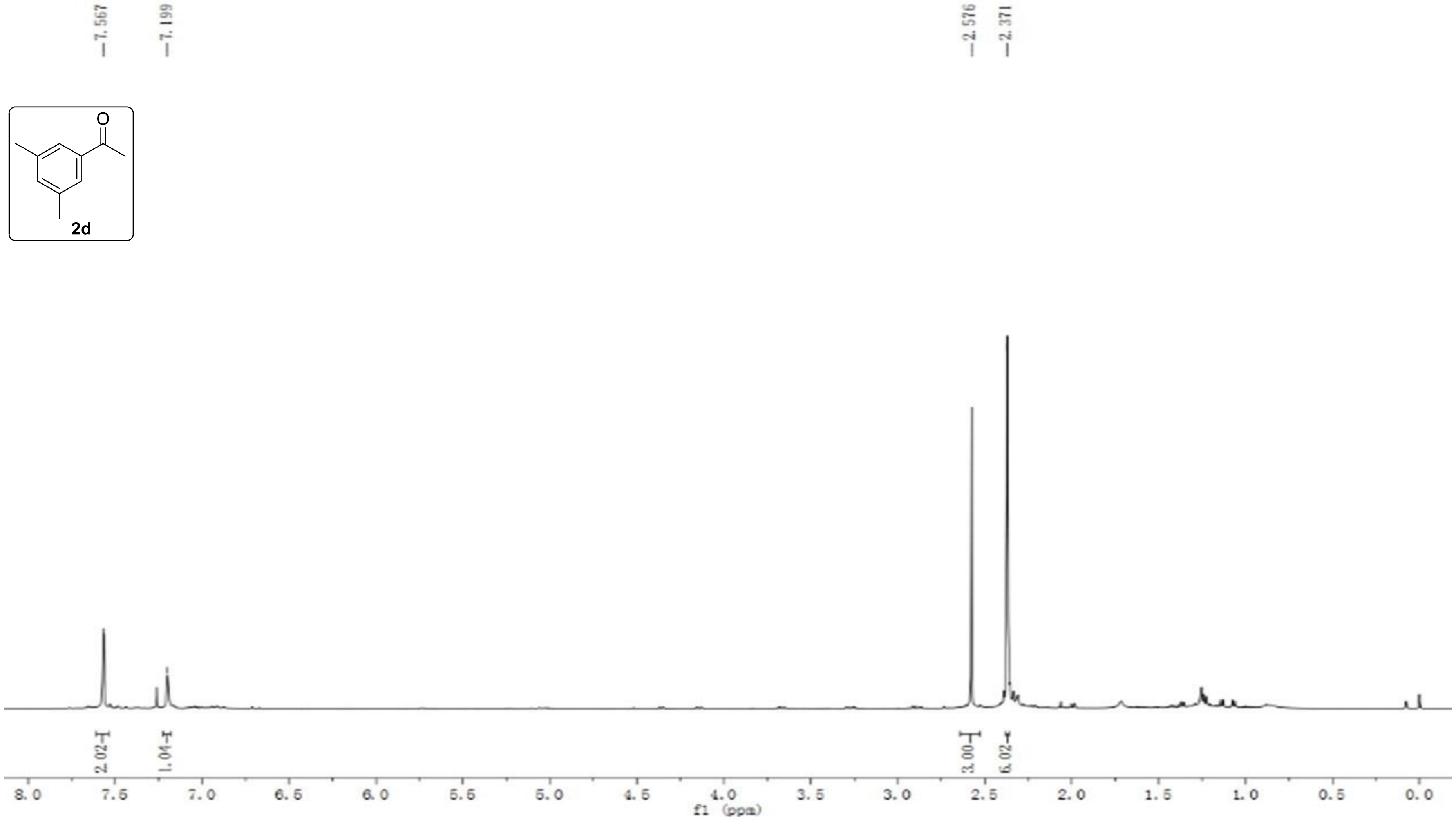

Supplementary Fig. 12. ^{13}C NMR of compound **2d**

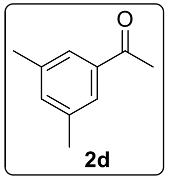

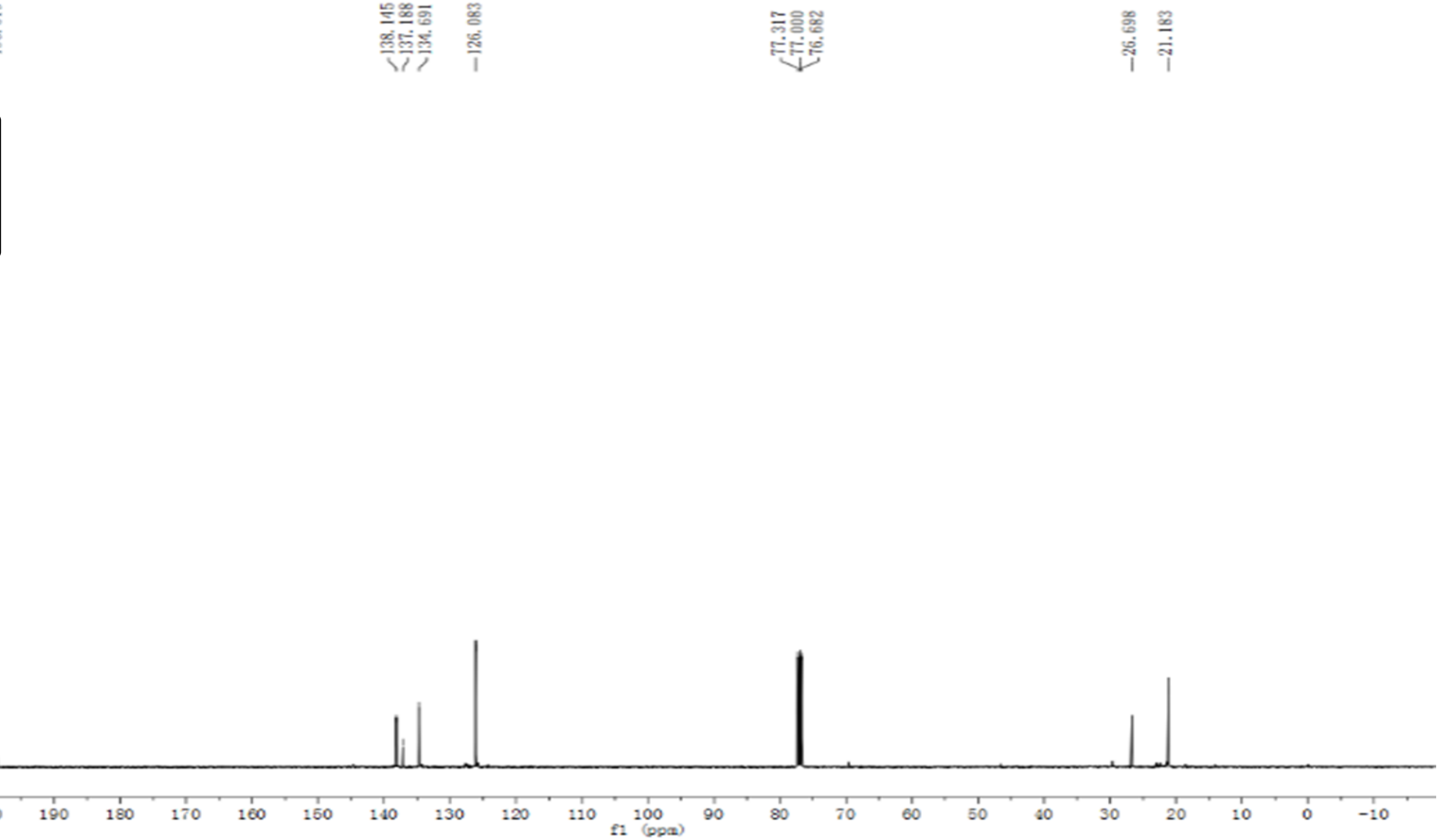

Supplementary Fig. 13.^{1}H NMR of compound **2e**

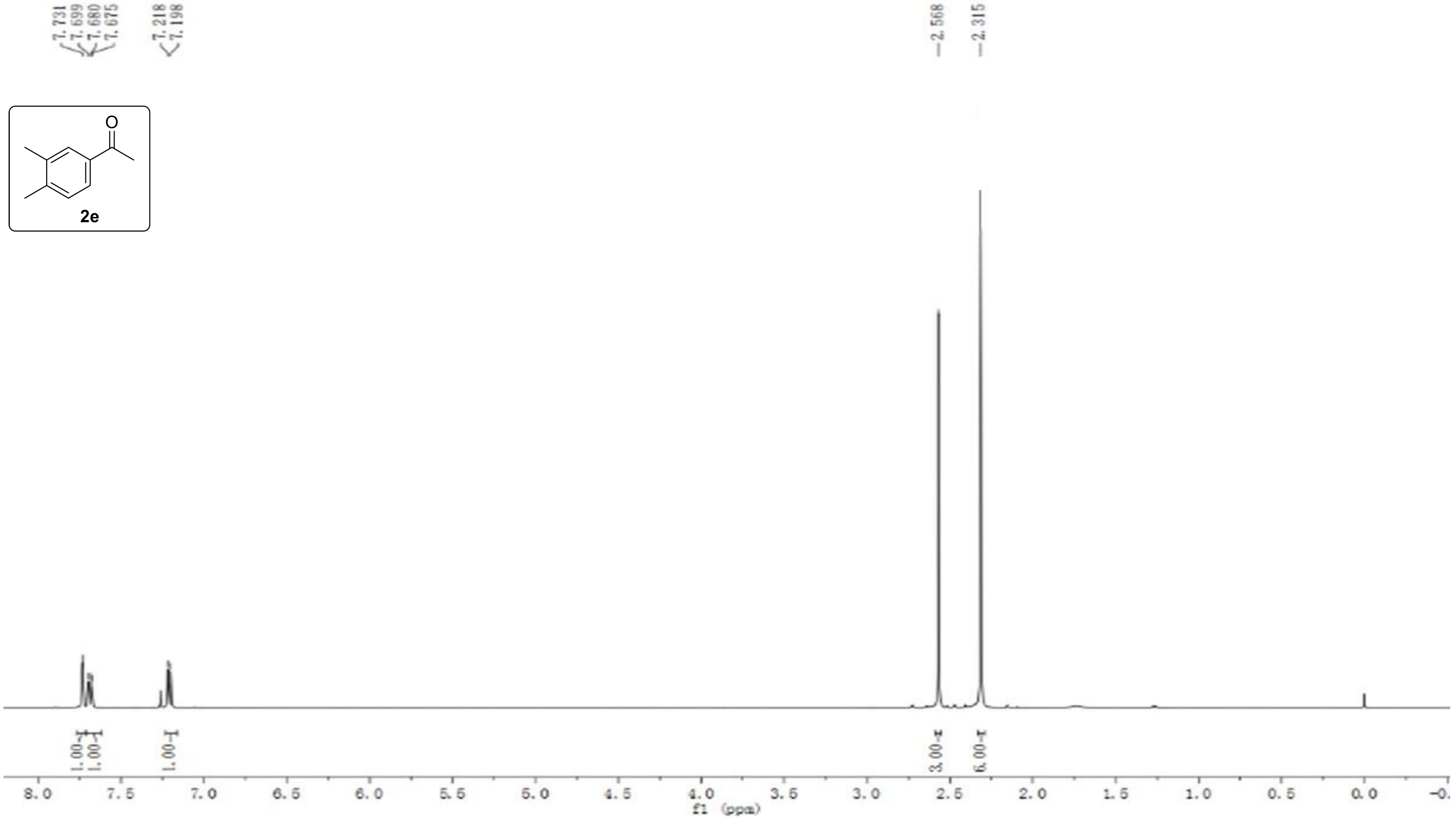

Supplementary Fig. 14.^{13}C NMR of compound **2e**

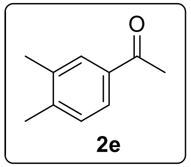

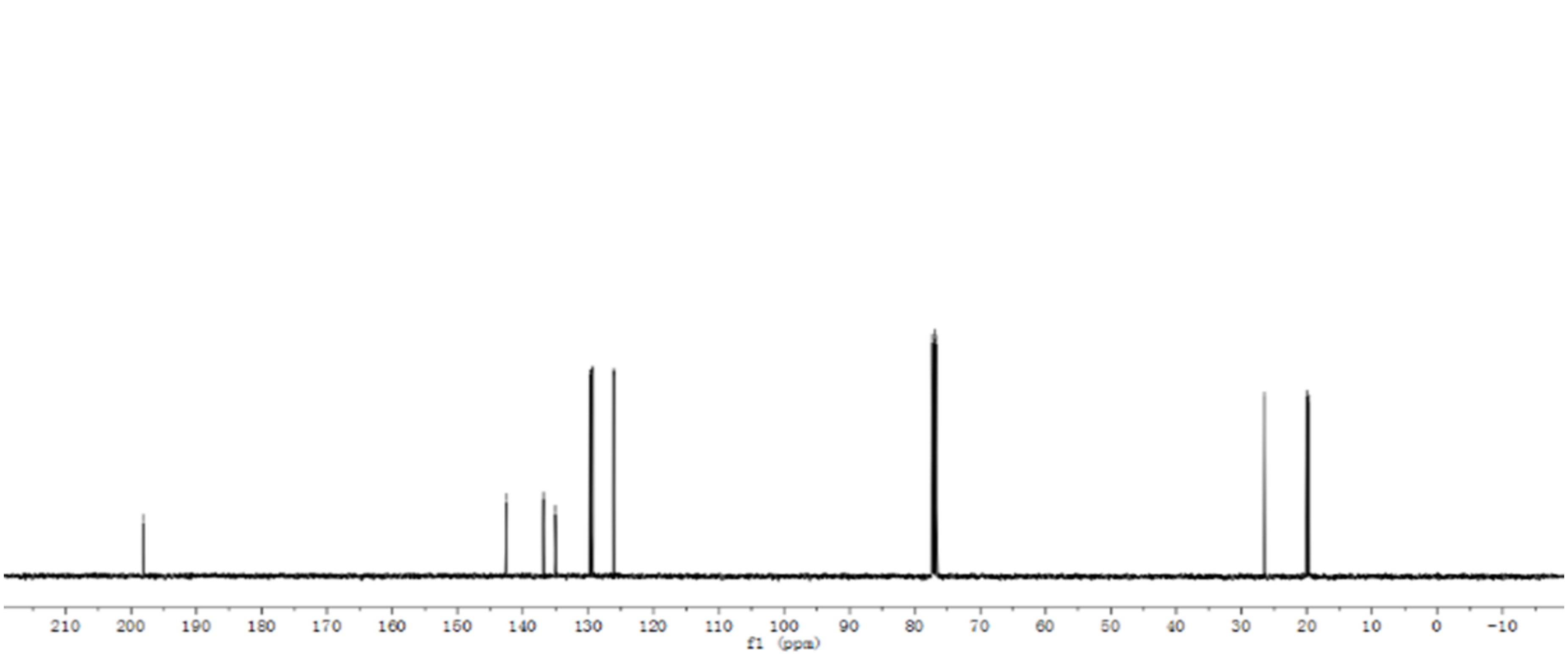

Supplementary Fig. 15. ^{1}H NMR of compound **2f**

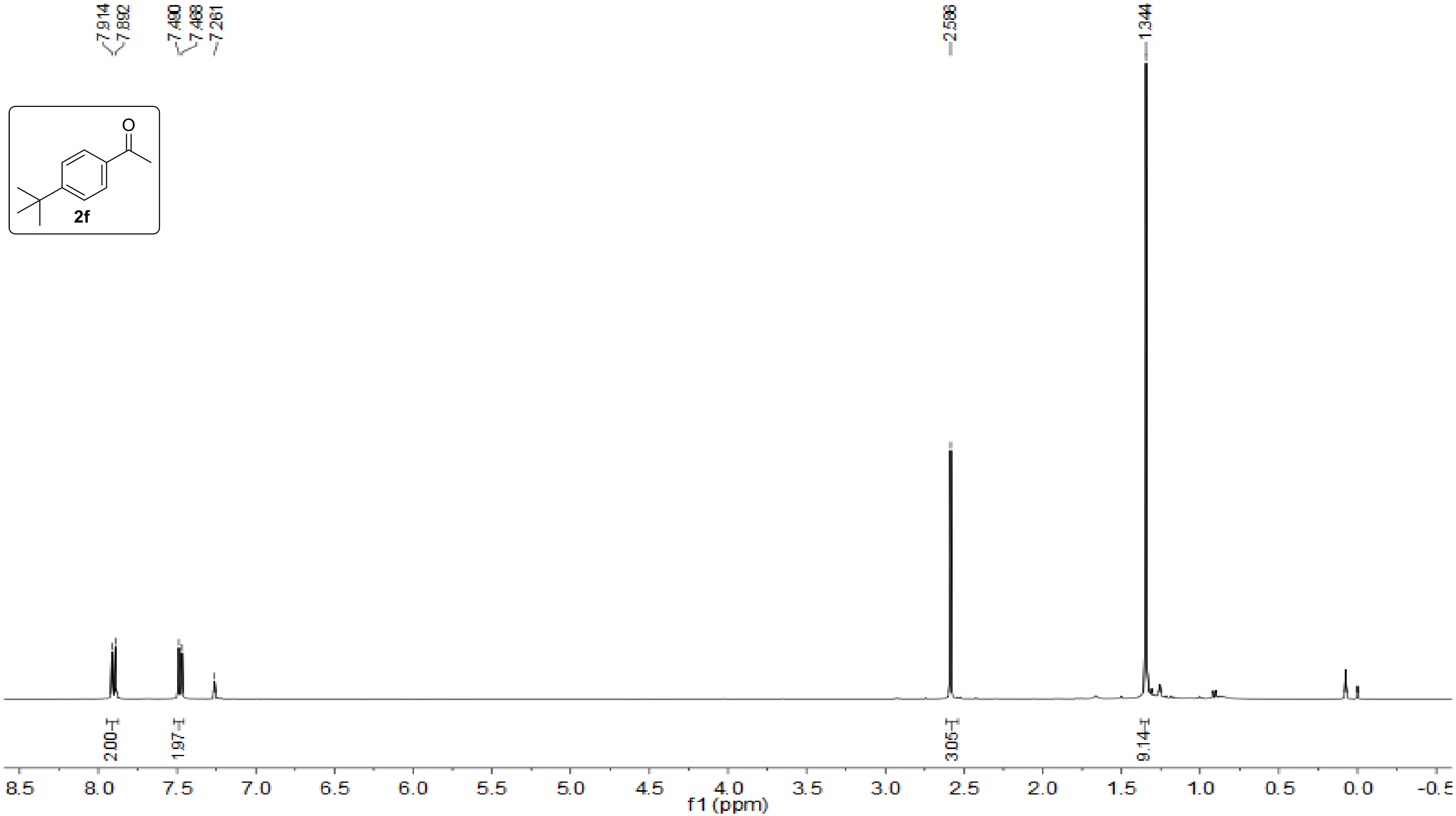

Supplementary Fig. 16. ^{13}C NMR of compound **2f**

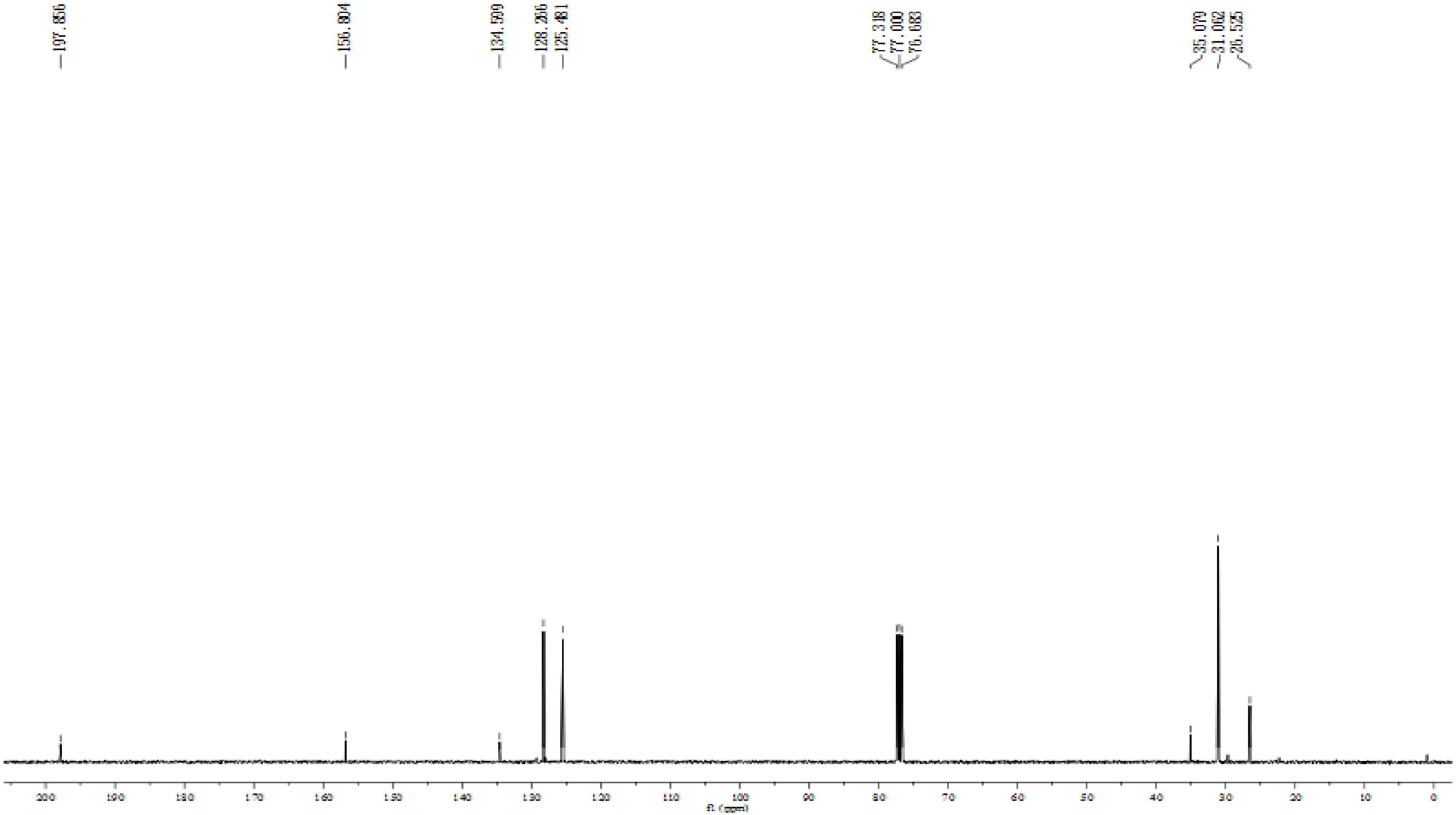

Supplementary Fig. 17.^{1}H NMR of compound **2g**

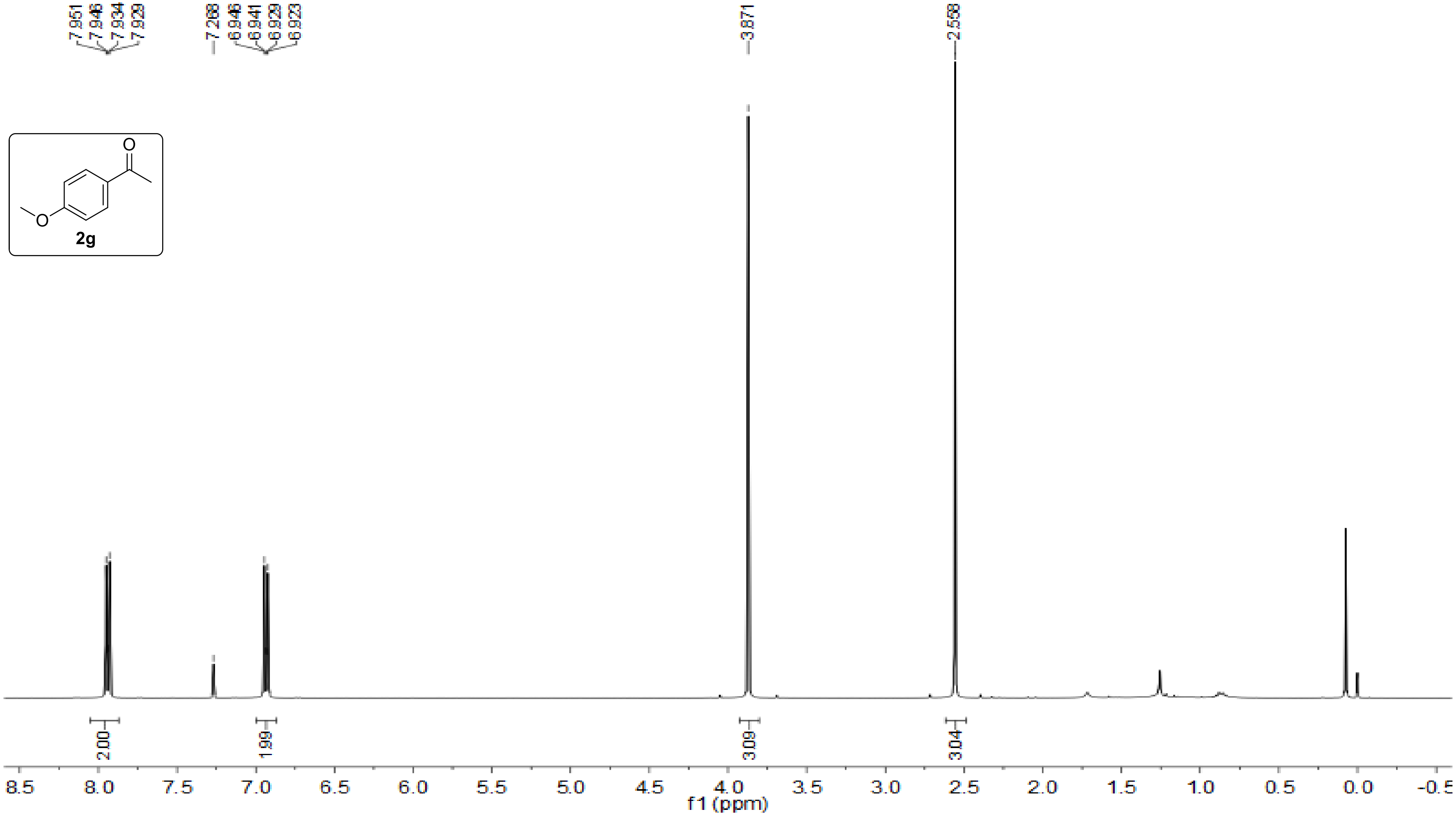

Supplementary Fig. 18.^{13}C NMR of compound **2g**

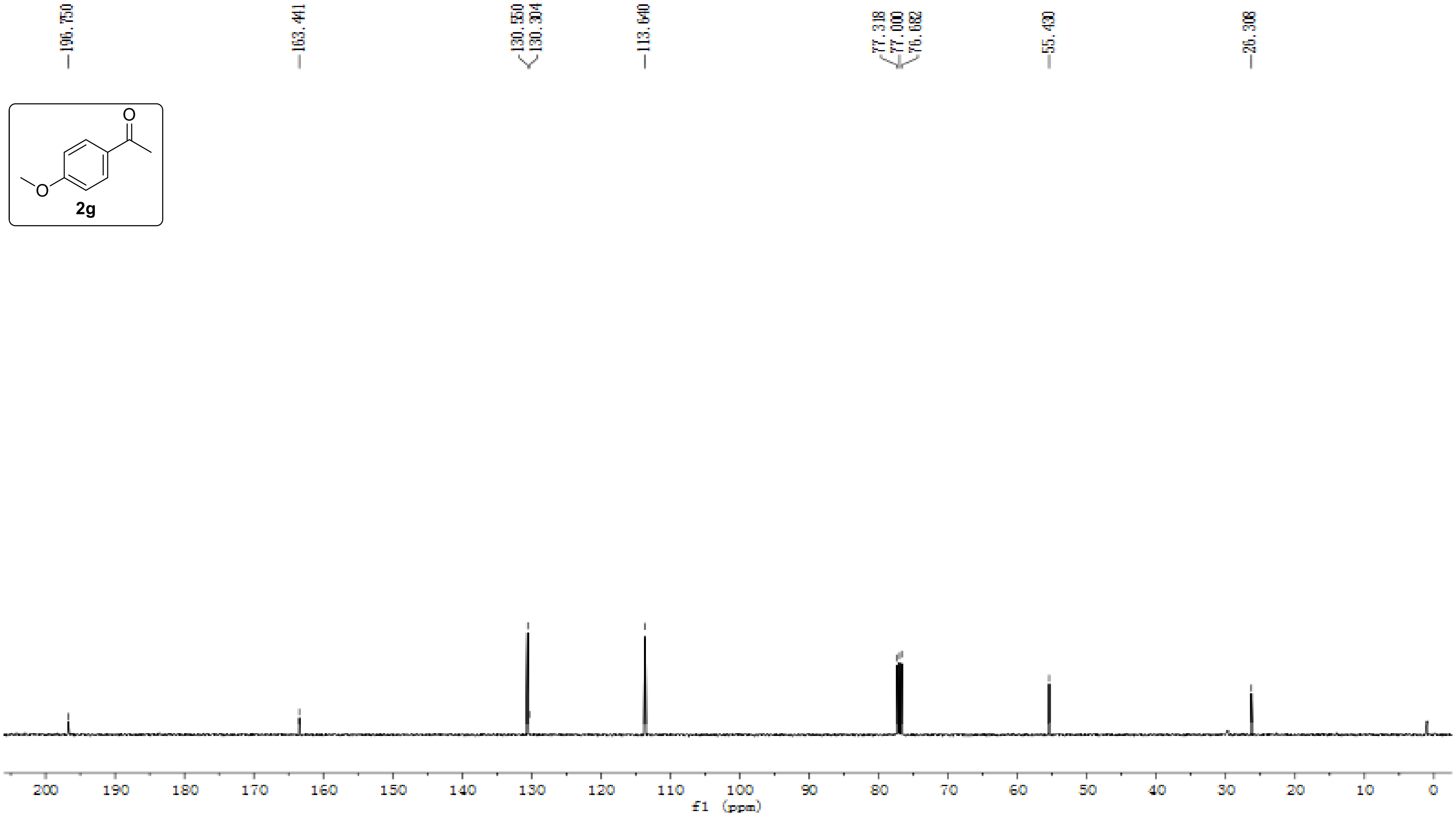

Supplementary Fig. 19.^{1}H NMR of compound **2h**

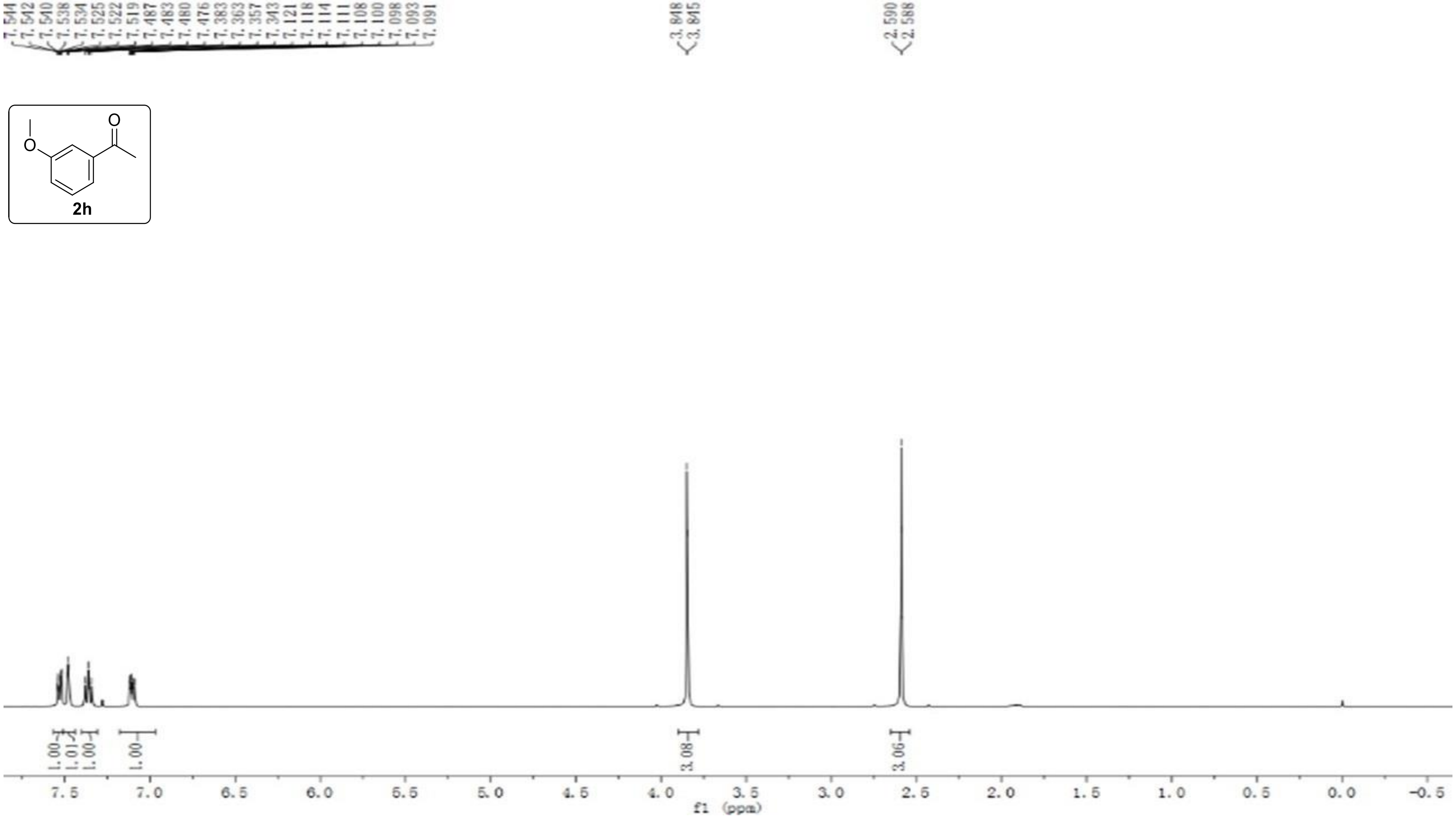

Supplementary Fig. 20.^{13}C NMR of compound **2h**

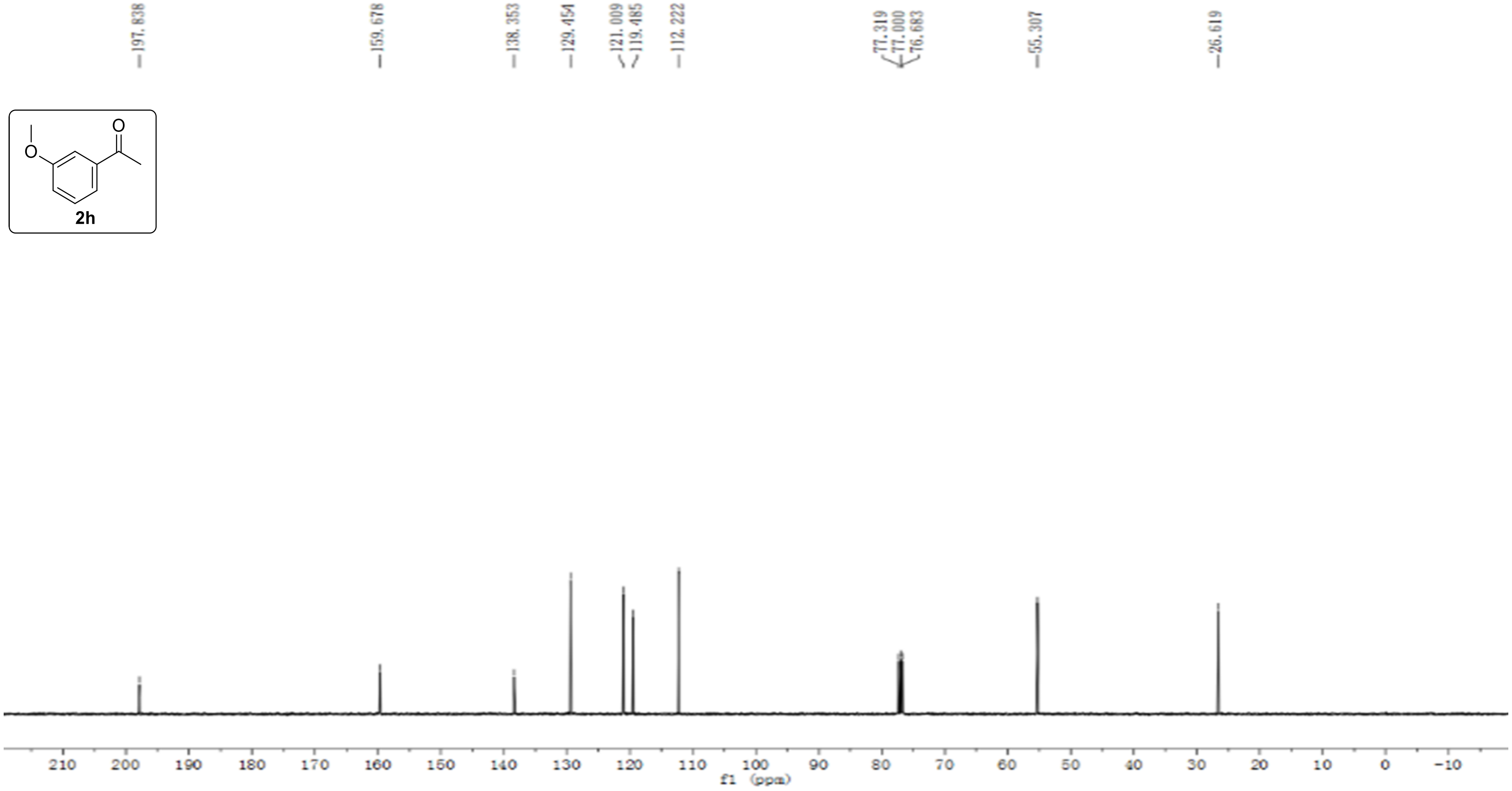

Supplementary Fig. 21. ^{1}H NMR of compound **2i**

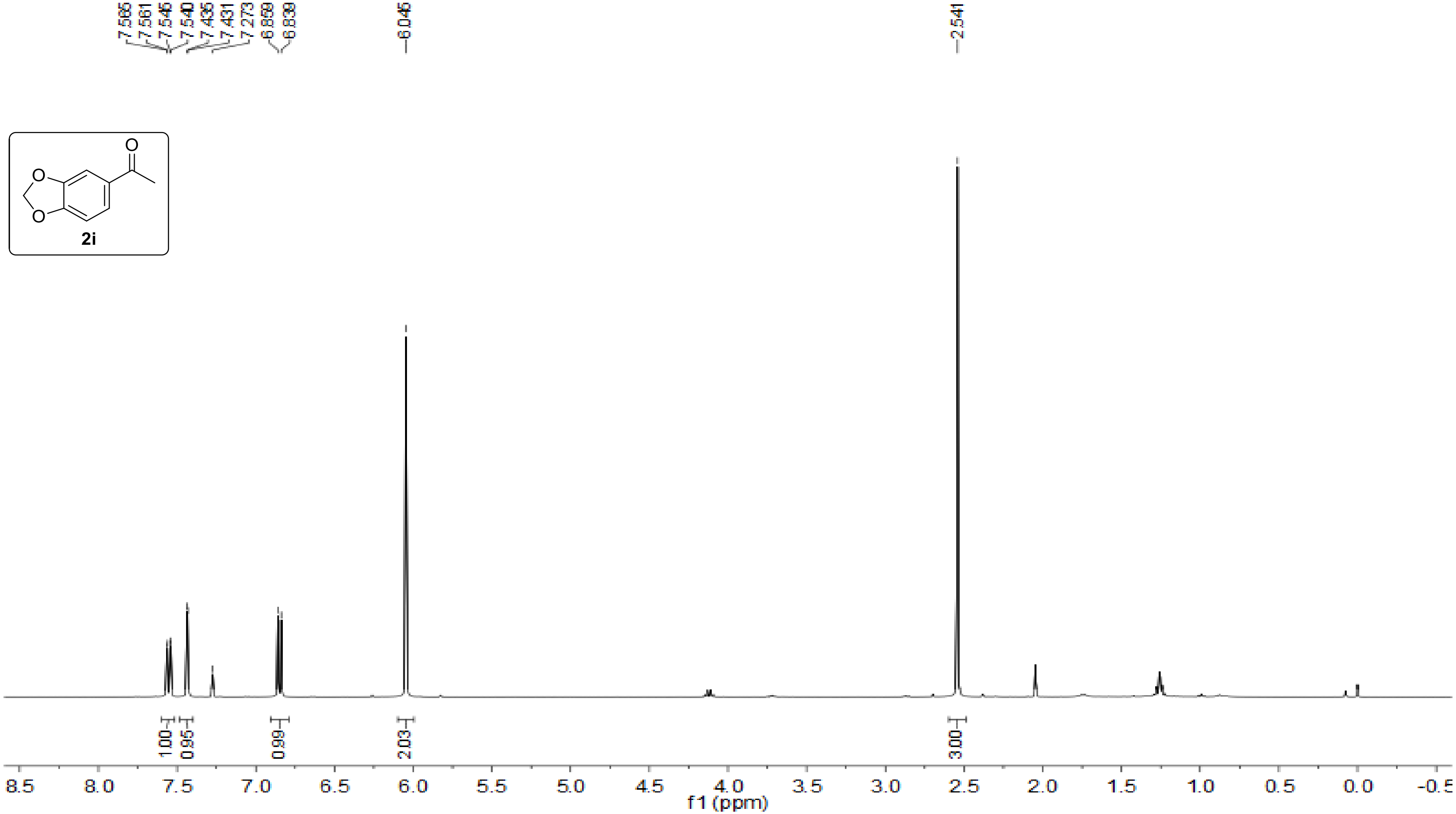

Supplementary Fig. 22. ^{13}C NMR of compound **2i**

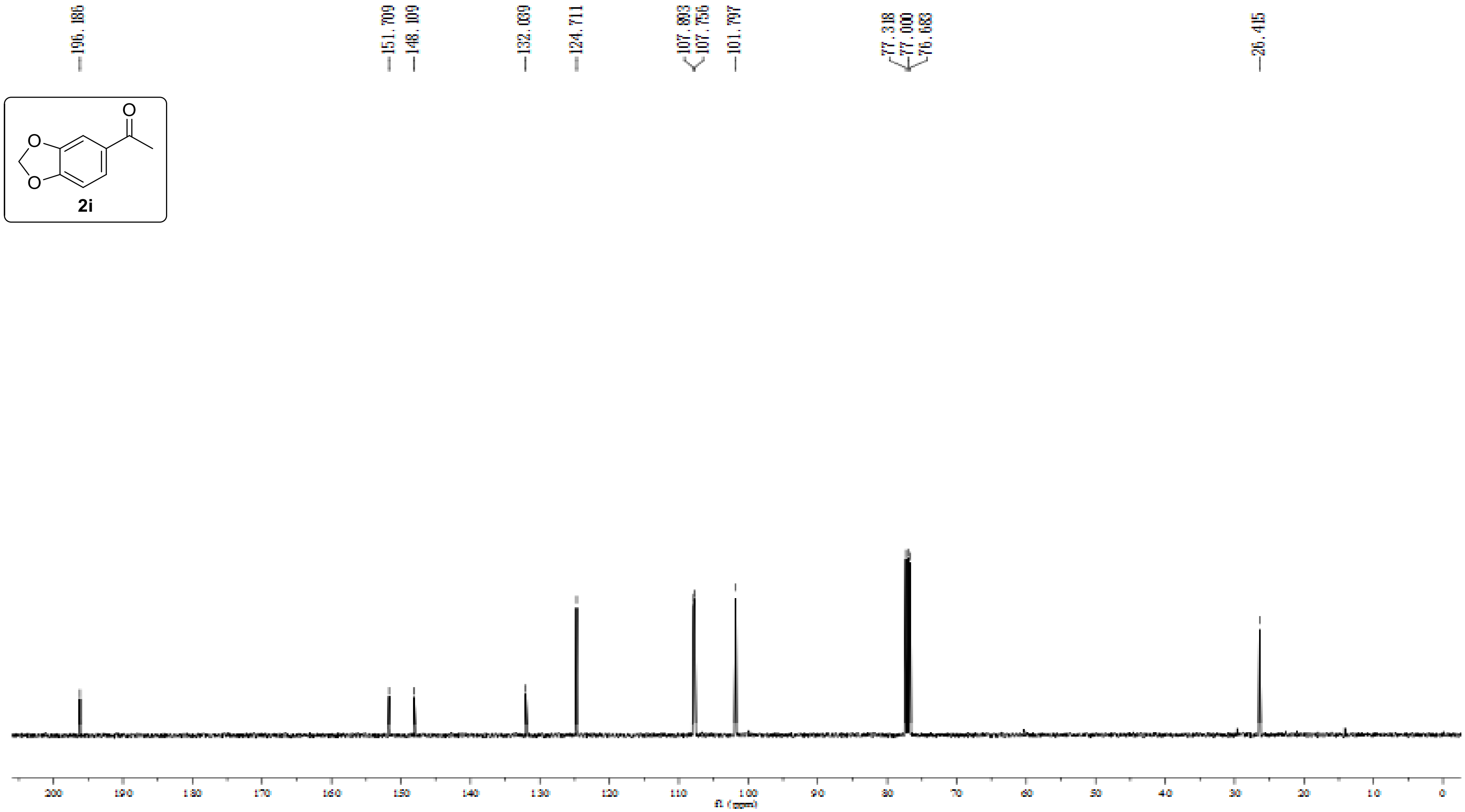

Supplementary Fig. 23.^{1}H NMR of compound **2j**

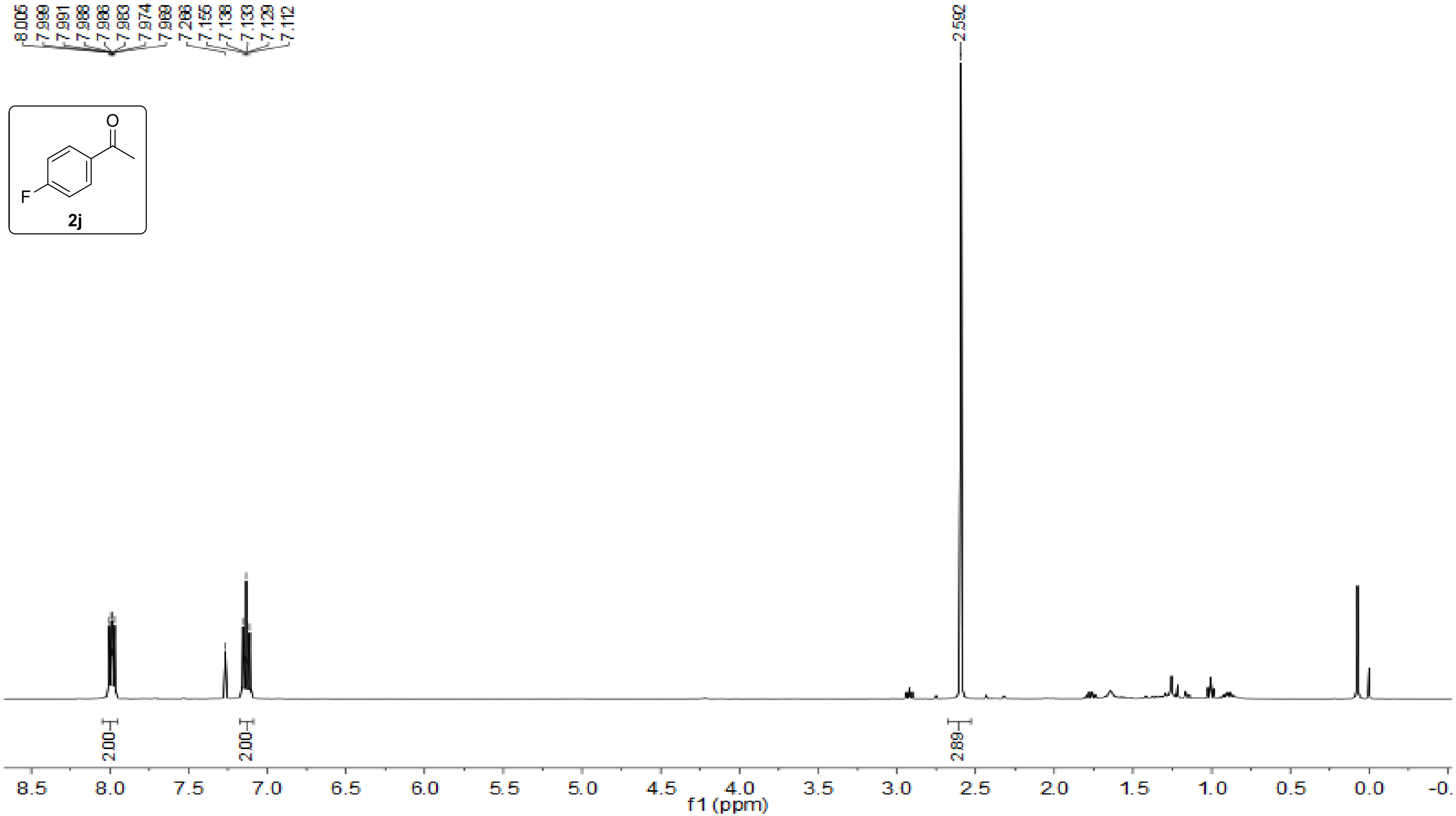

Supplementary Fig. 24.^{13}C NMR of compound **2j**

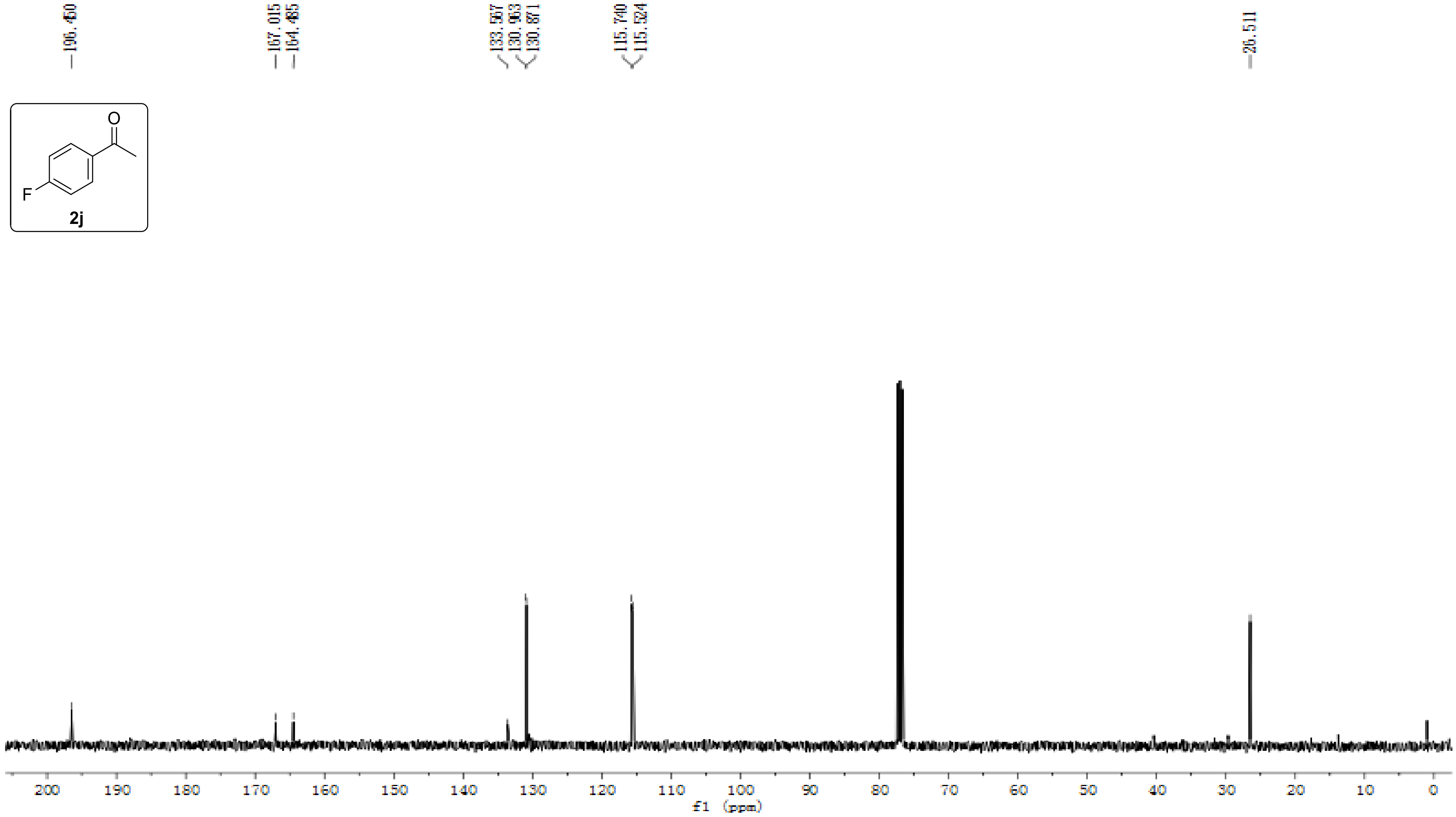

2j

Supplementary Fig. 25. ^{1}H NMR of compound **2k**

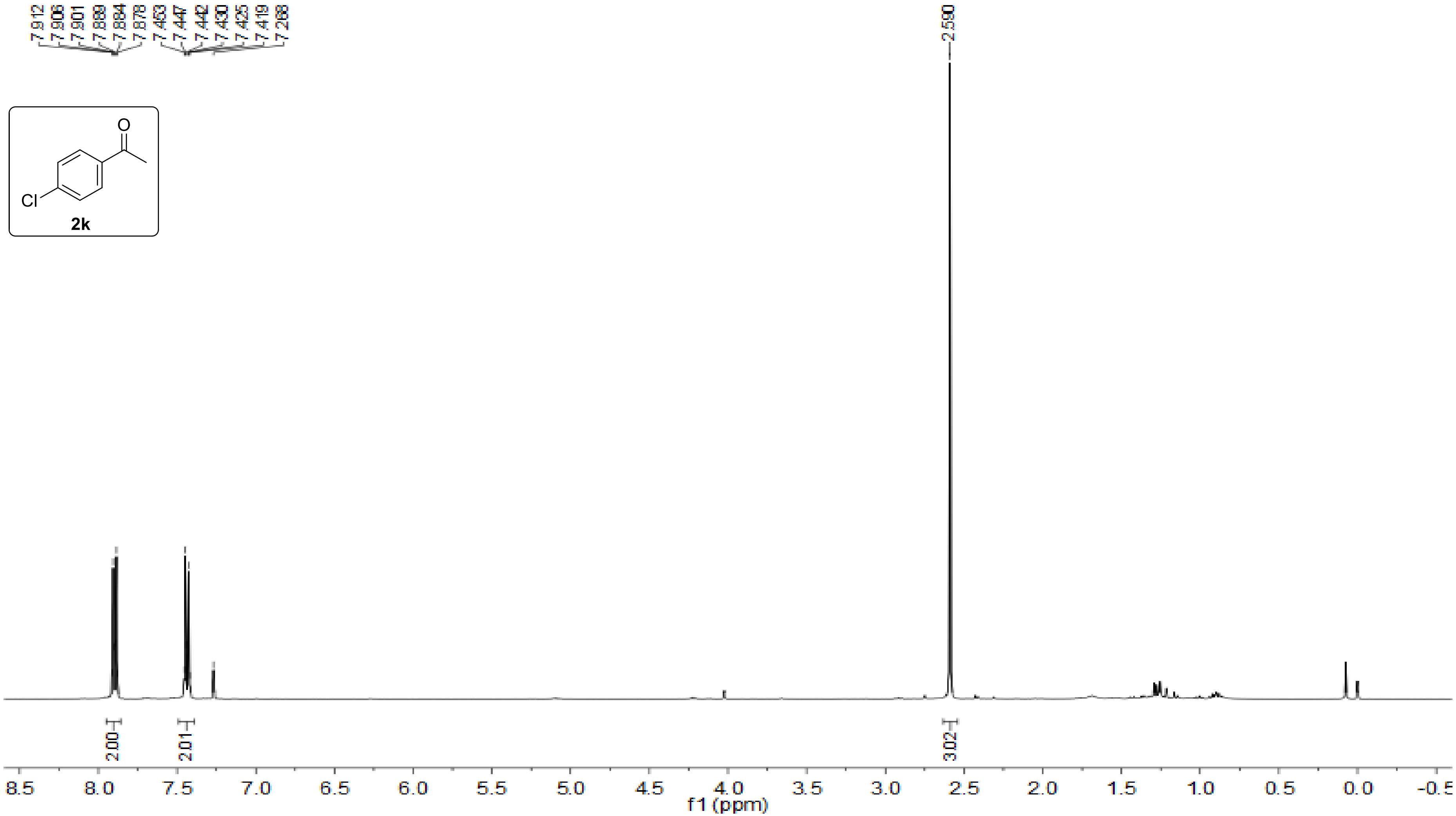

Supplementary Fig. 26.^{13}C NMR of compound **2k**

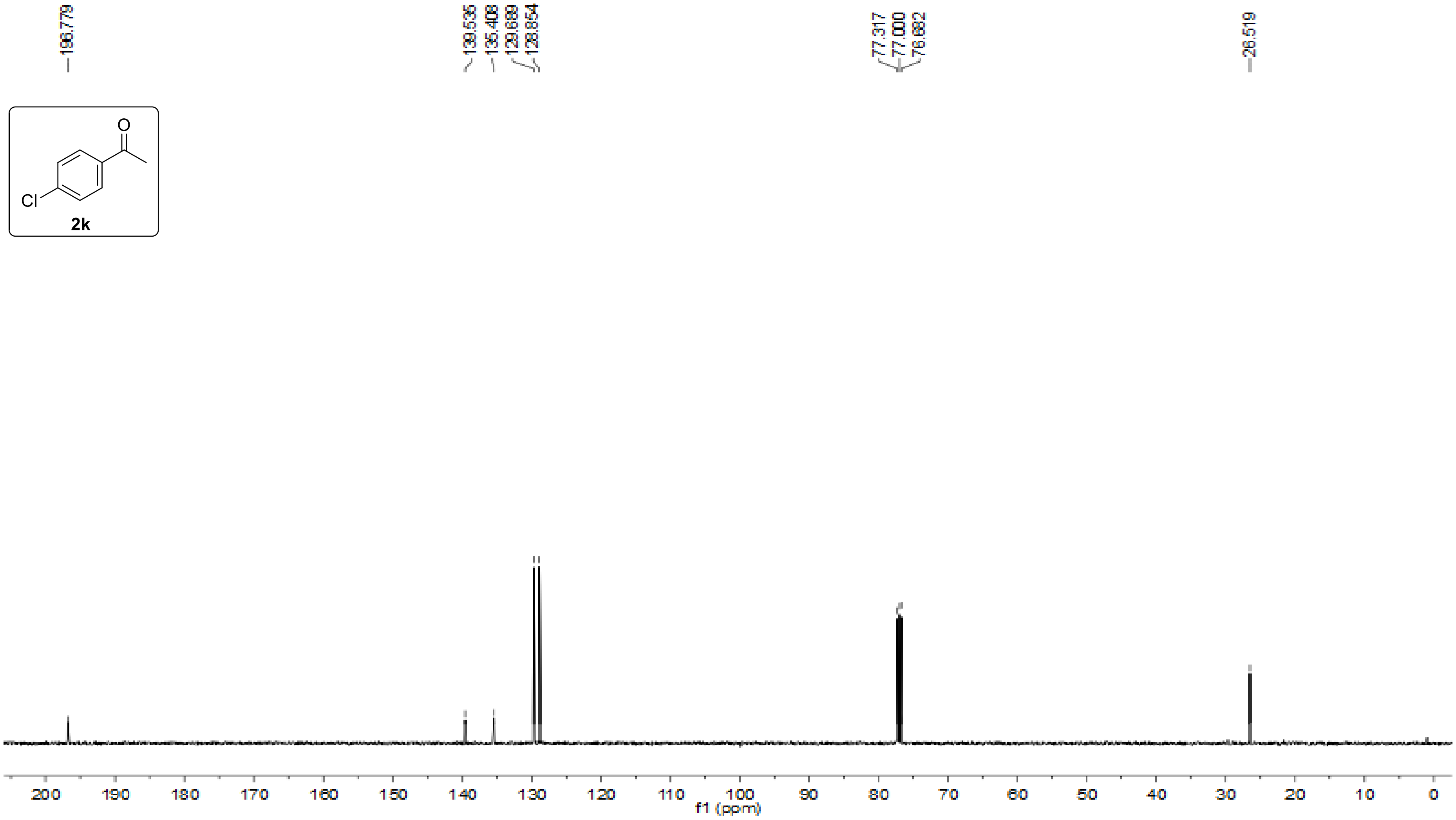

Supplementary Fig. 27. ^{1}H NMR of compound **2l**

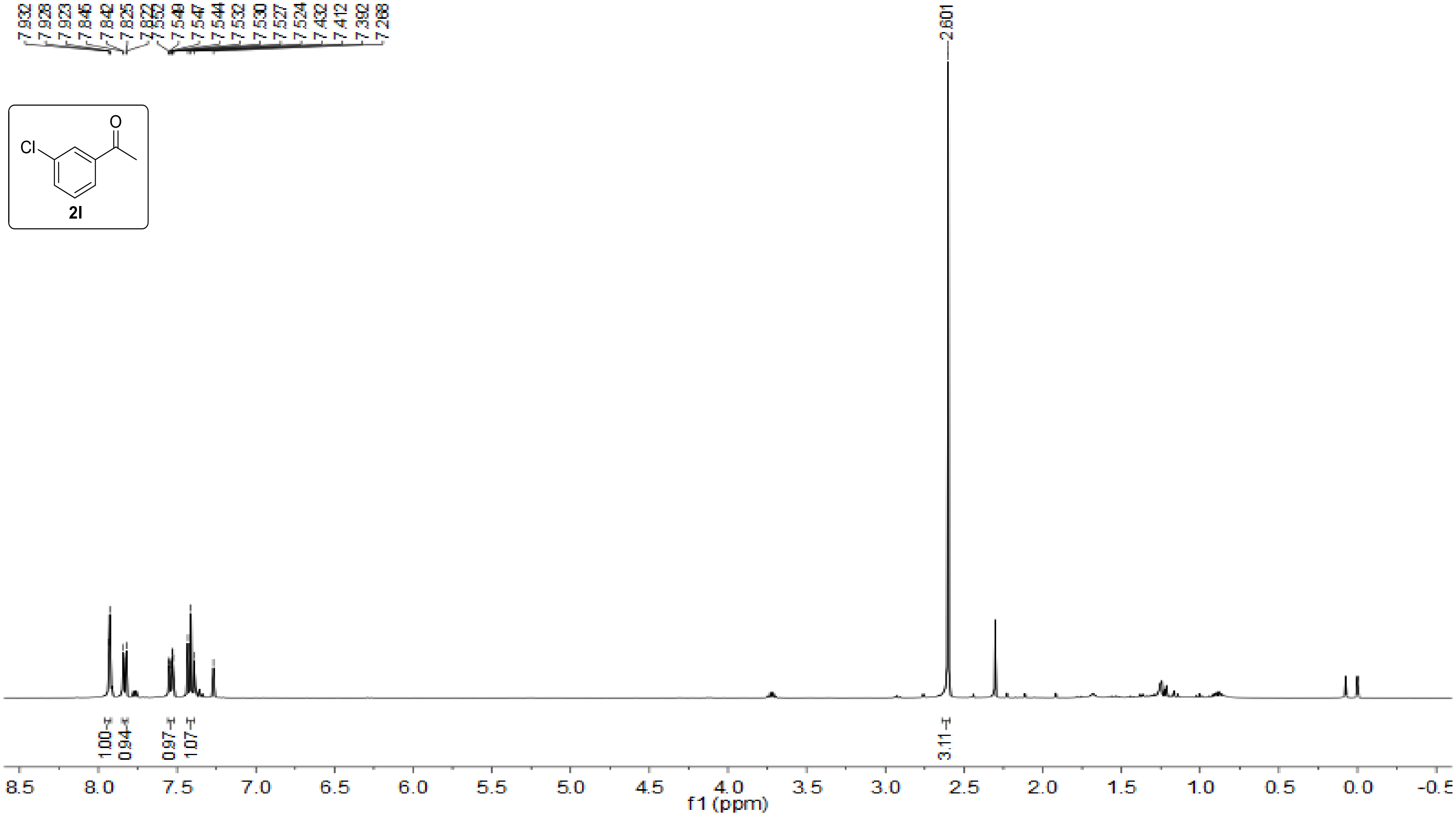

Supplementary Fig. 28. ^{13}C NMR of compound **2l**

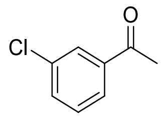

2l

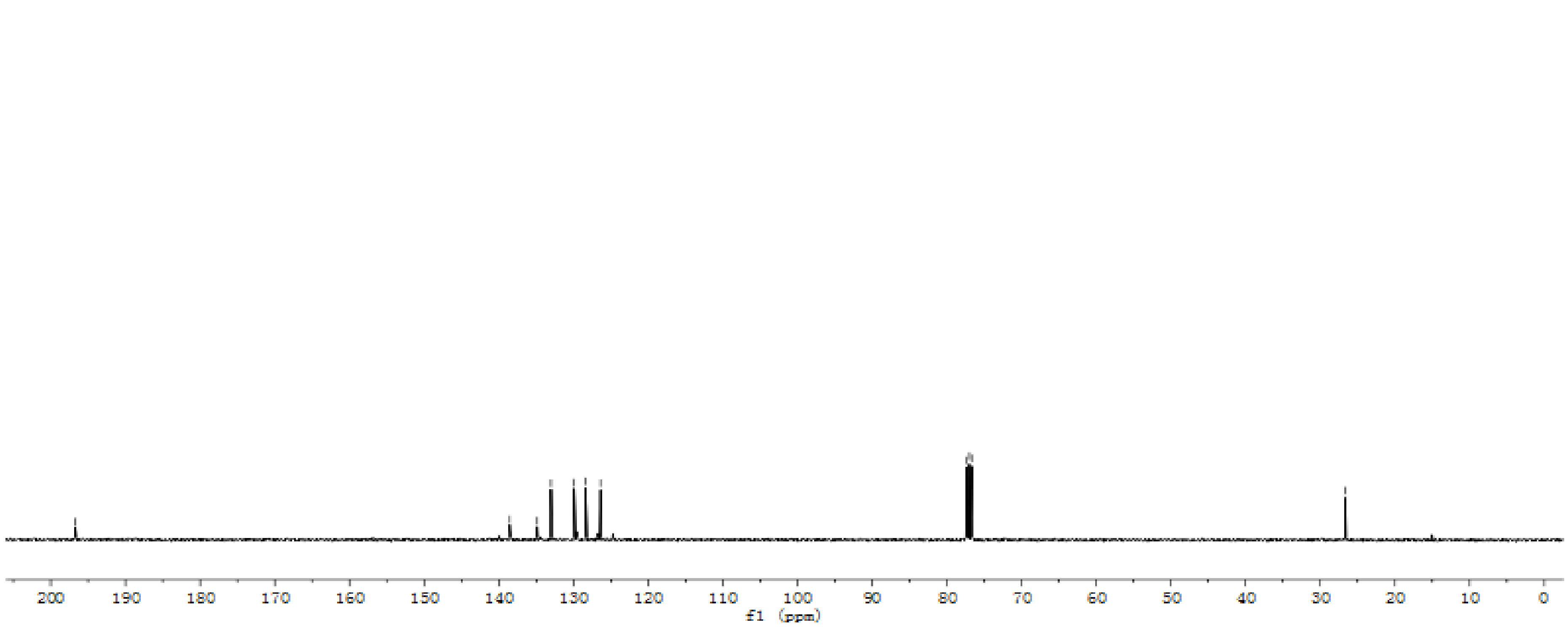

Supplementary Fig. 29. ^{1}H NMR of compound **2m**

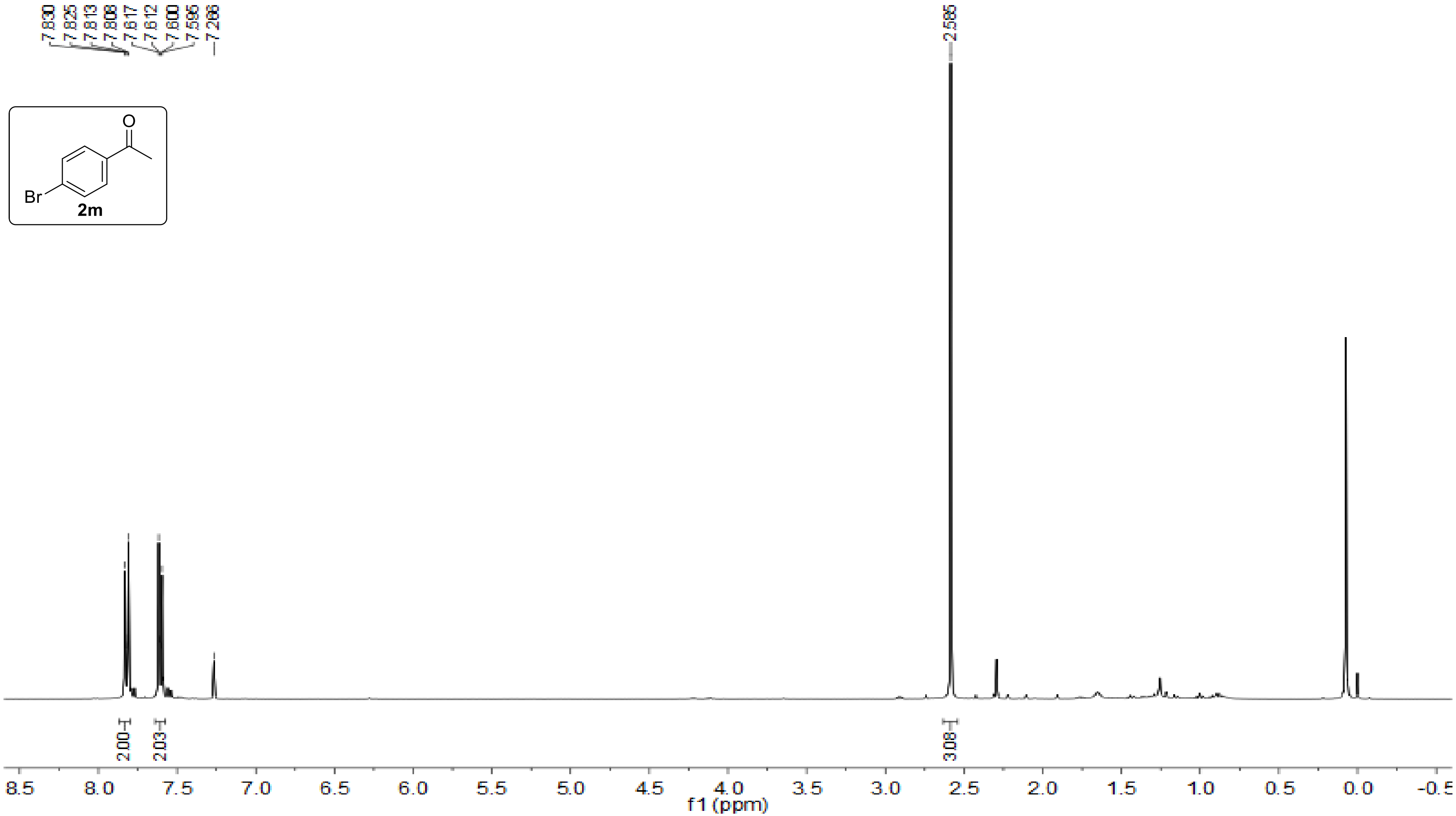

Supplementary Fig. 30.^{13}C NMR of compound **2m**

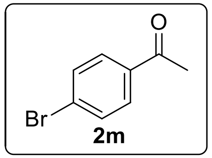

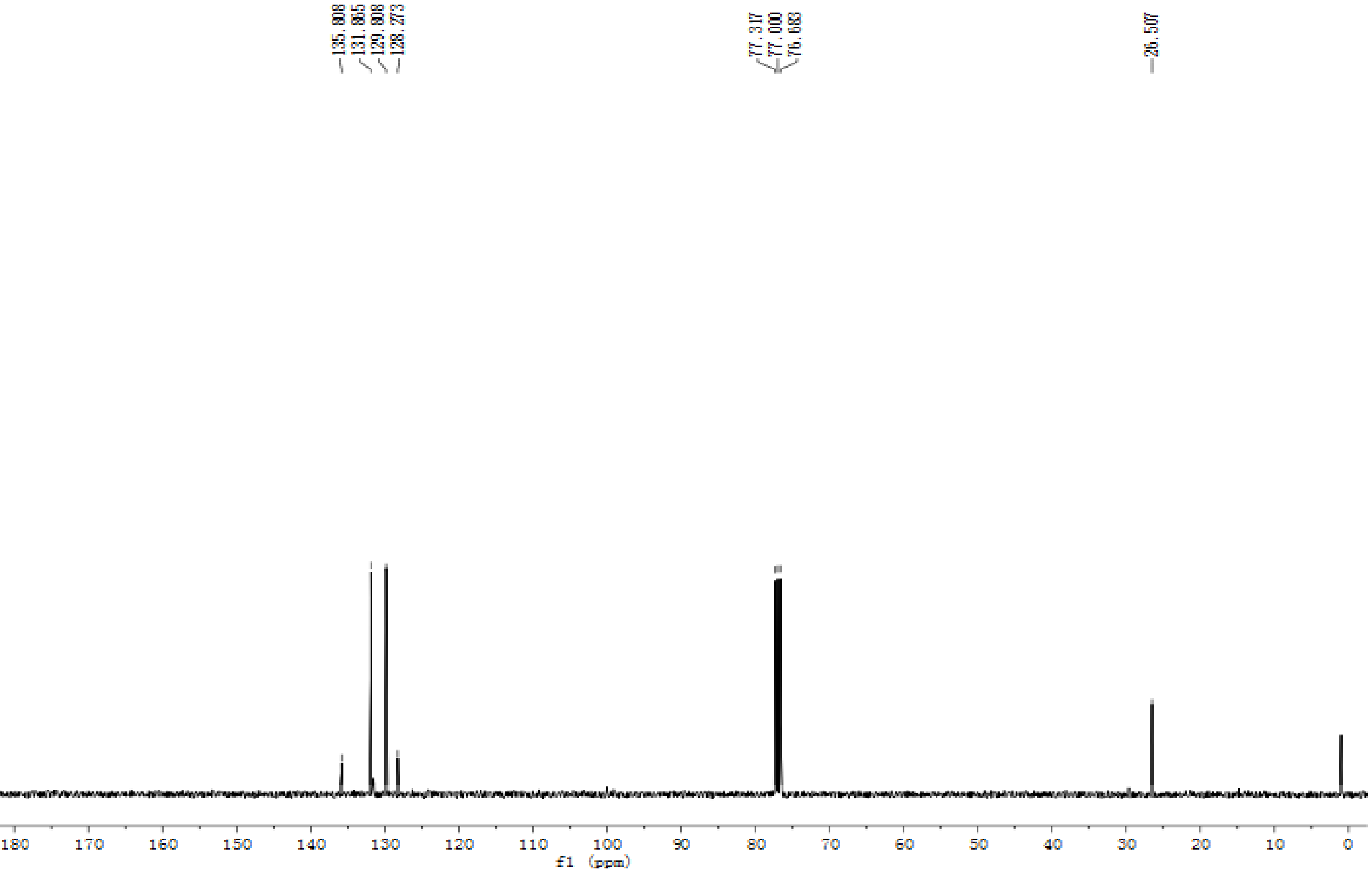

Supplementary Fig. 31.^{1}H NMR of compound **2n**

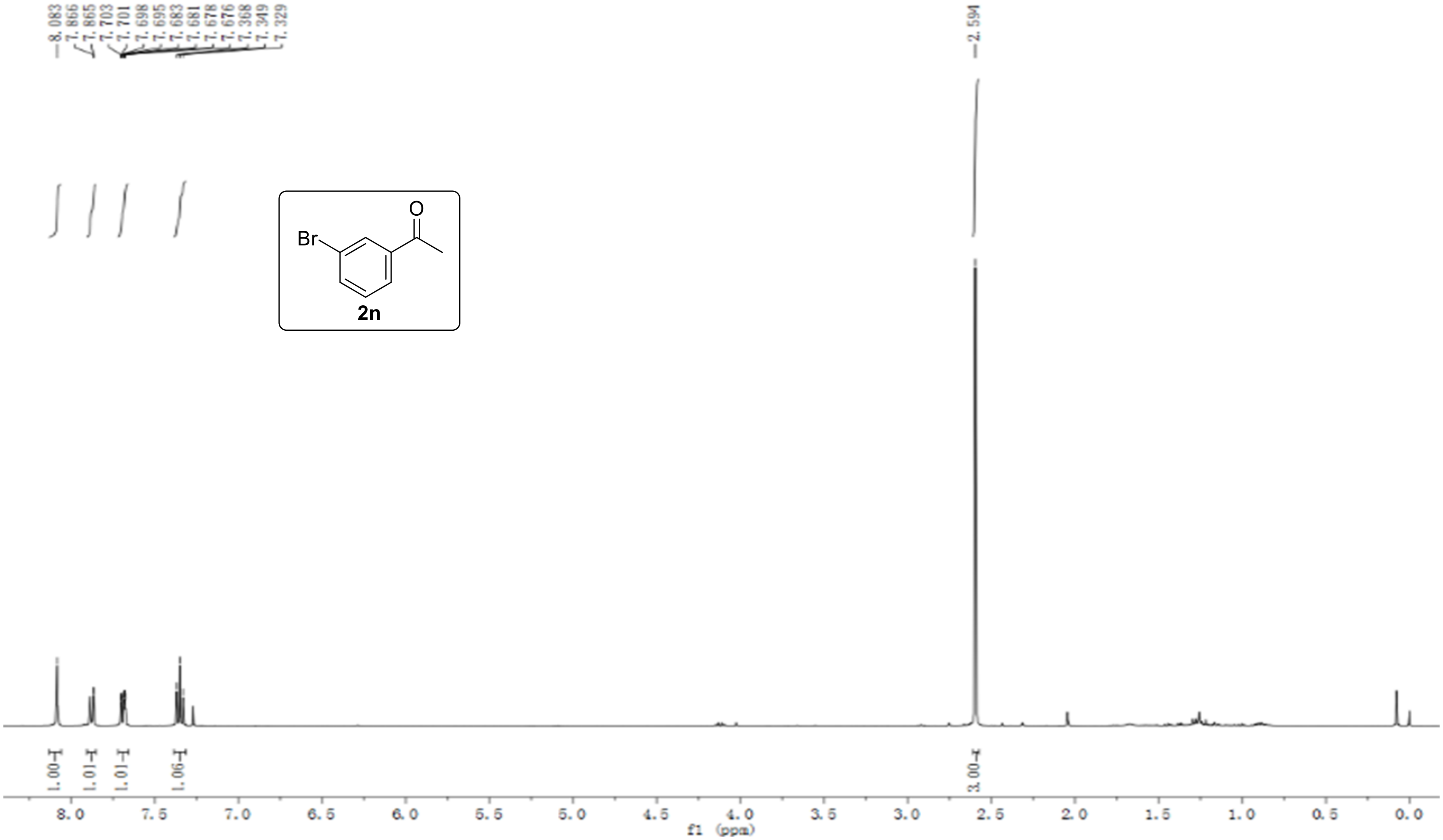

^{1} **Supplementary Fig. 32.**^{3}C NMR of compound **2n**

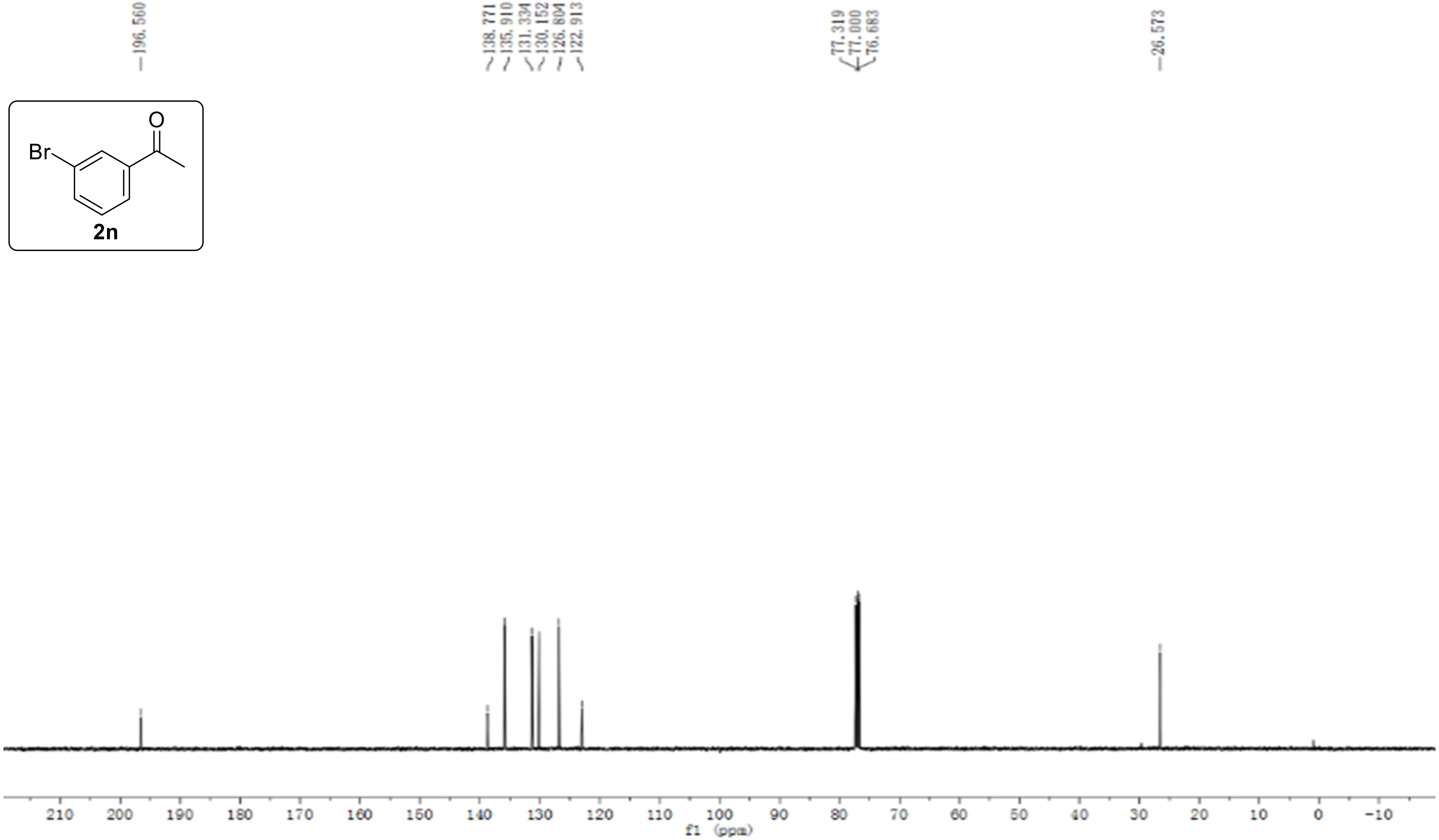

Supplementary Fig. 33. ^{1}H NMR of compound **2o**

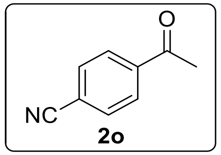

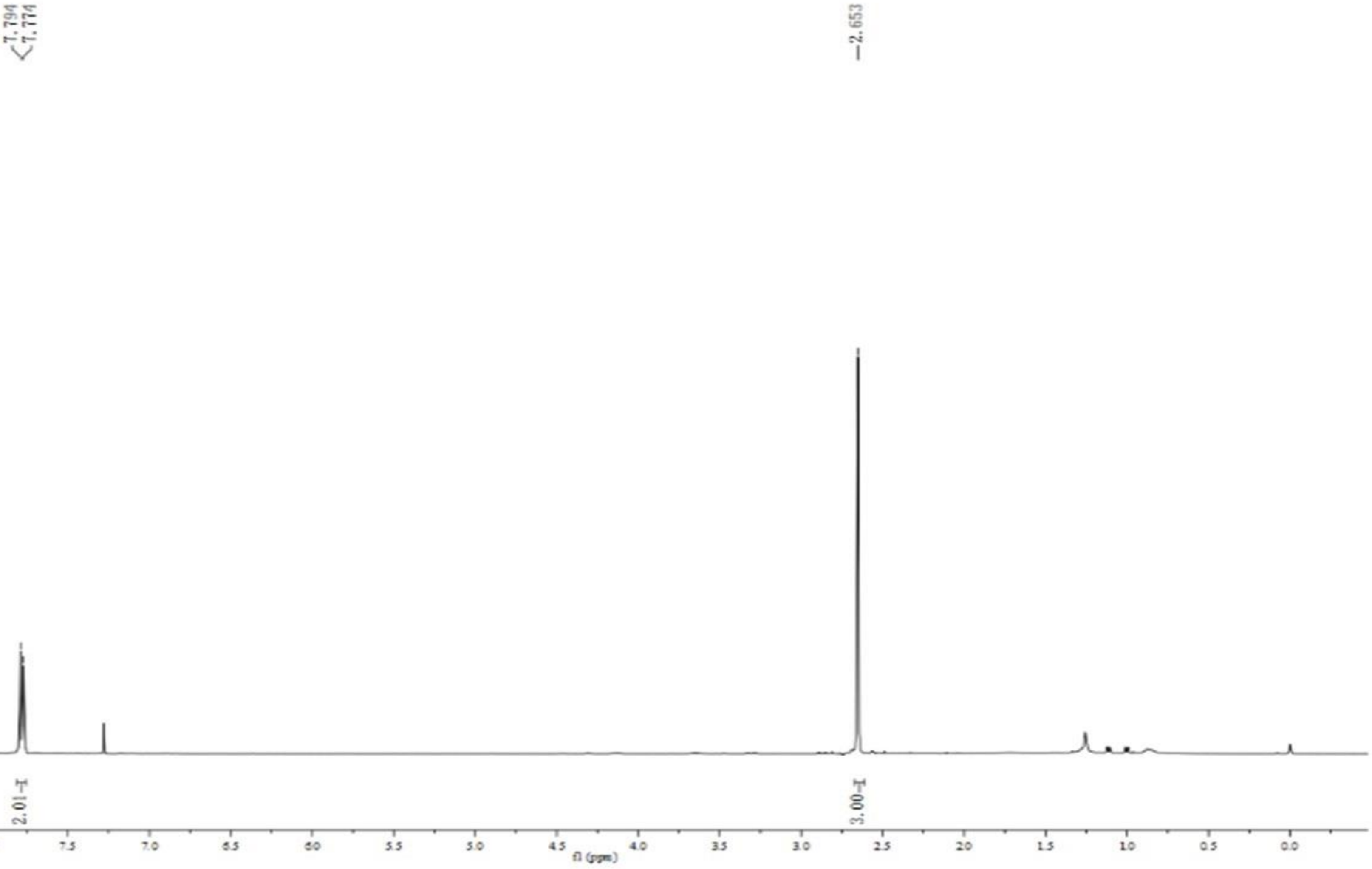

Supplementary Fig. 34. ^{13}C NMR of compound **2o**

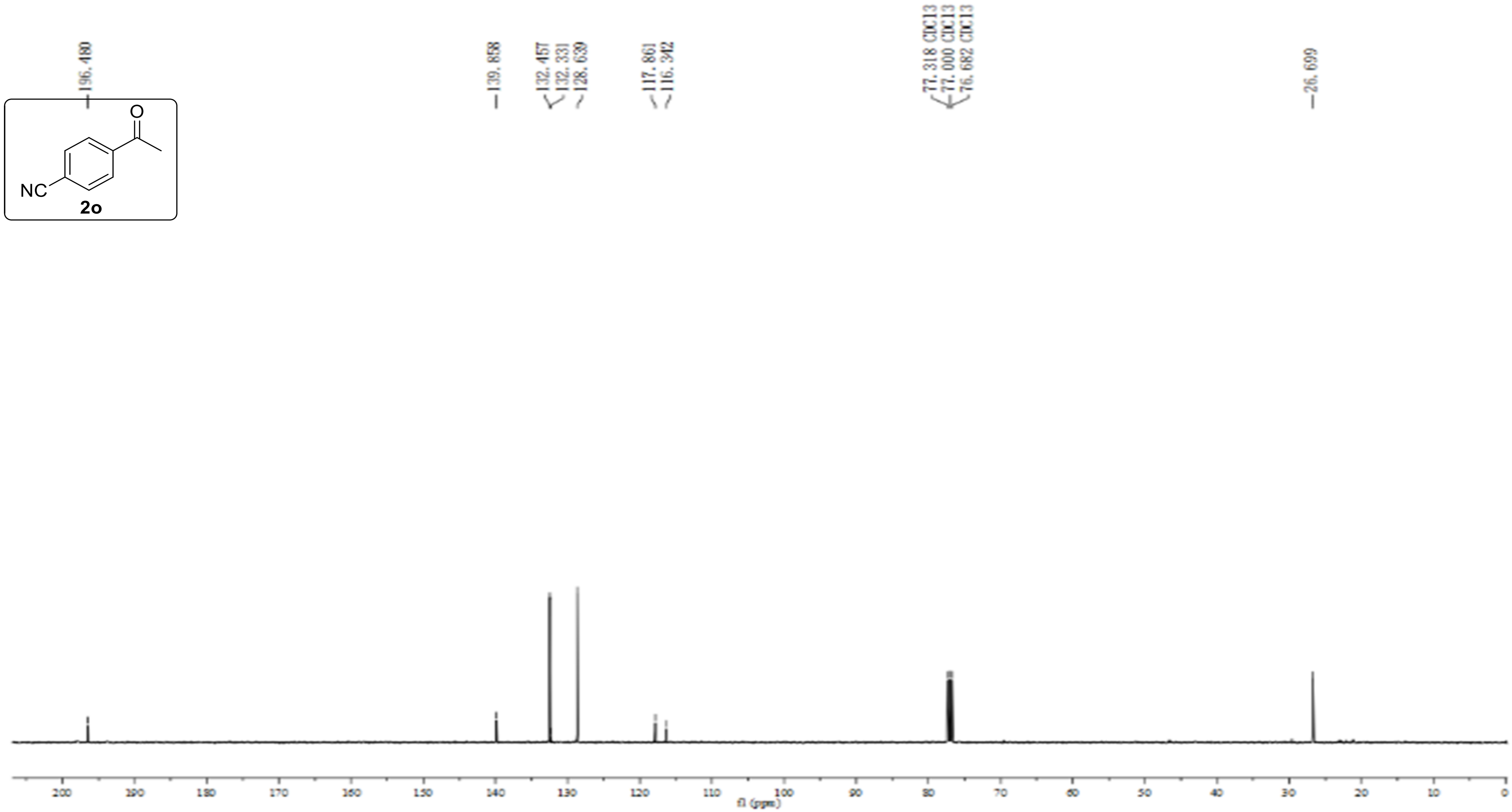

Supplementary Fig. 35.^{1}H NMR of compound **2p**

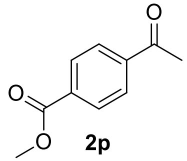

2p

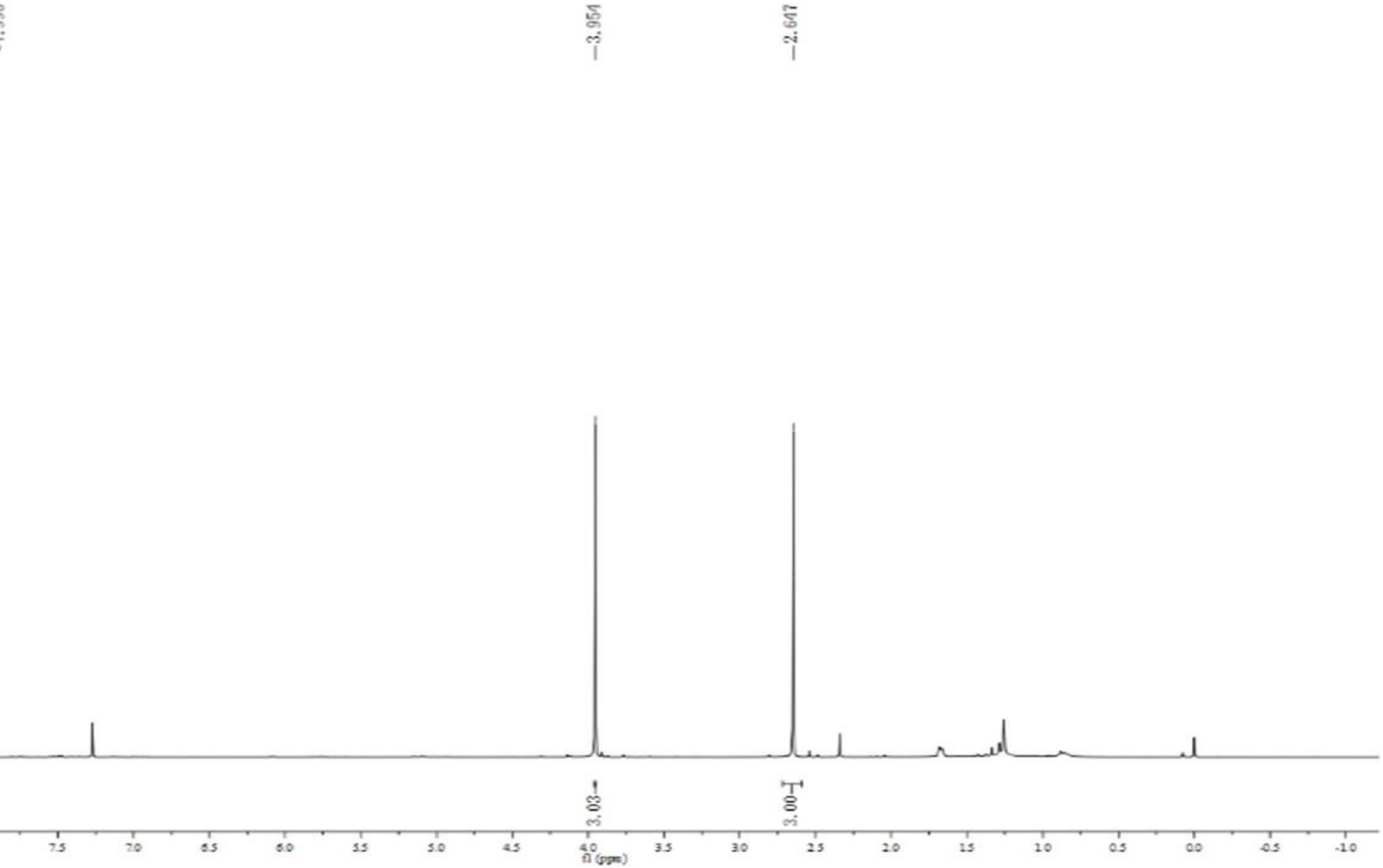

Supplementary Fig. 36. ^{13}C NMR of compound **2p**

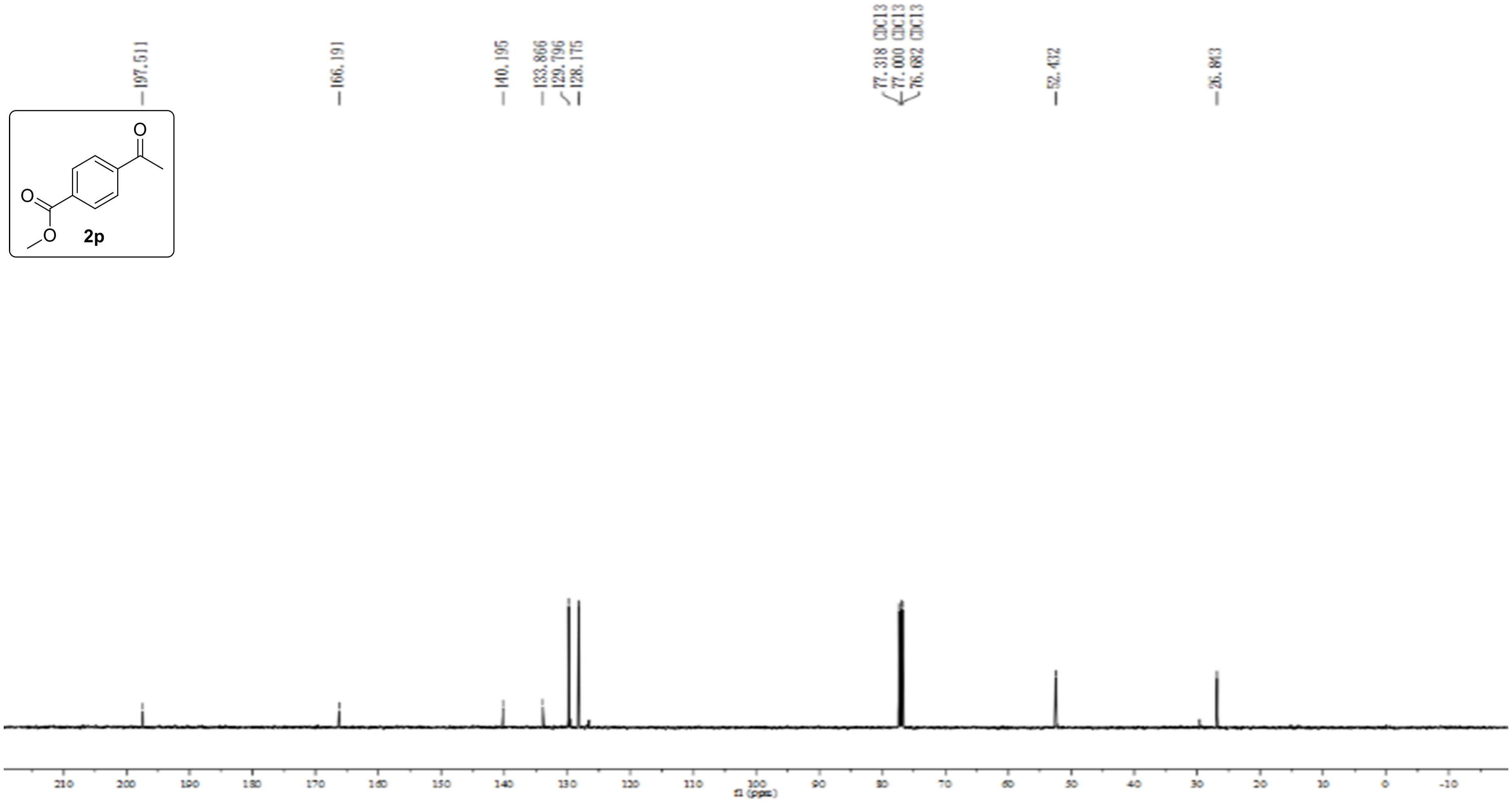

Supplementary Fig. 37.^{1}H NMR of compound **2q**

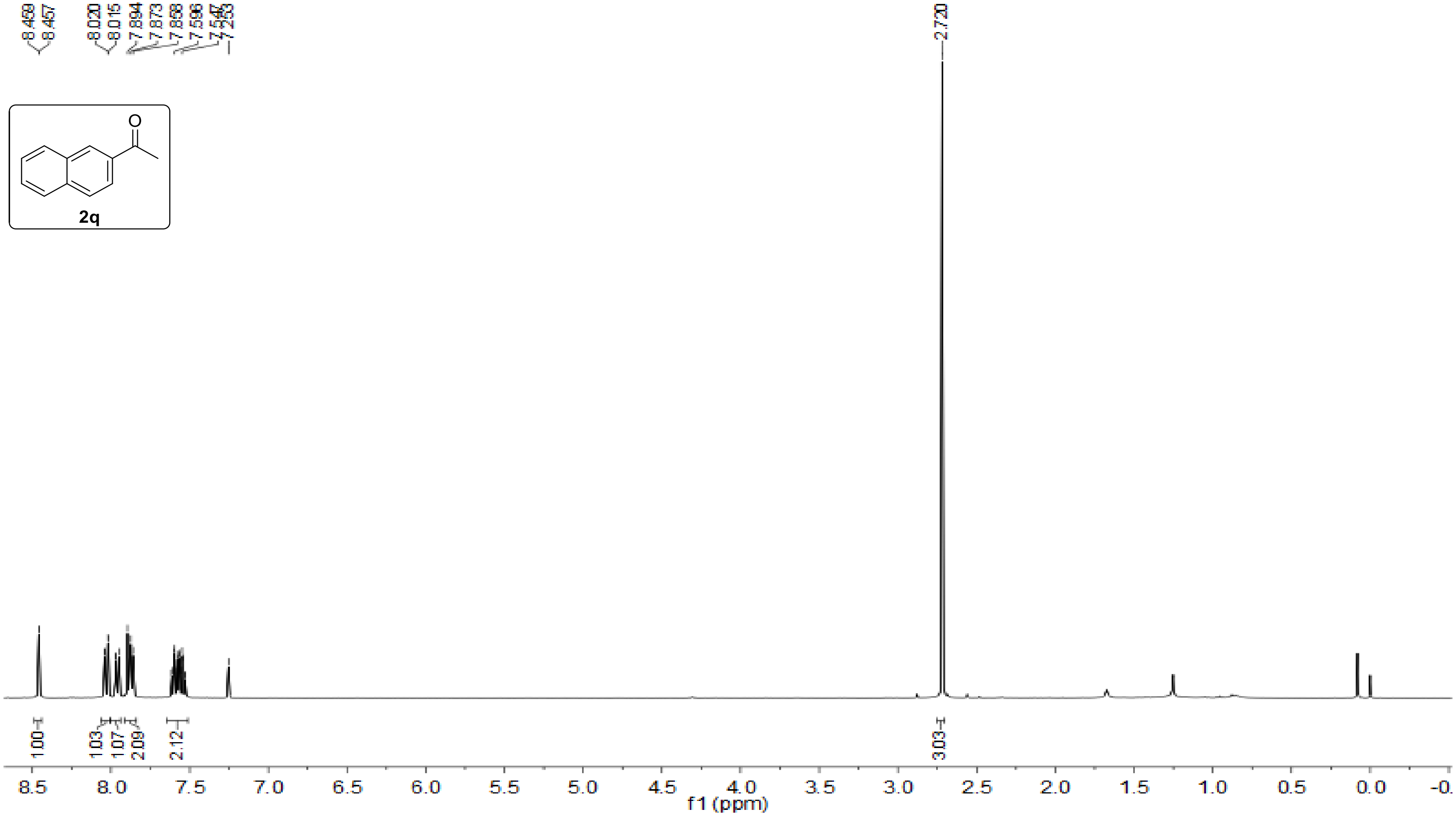

Supplementary Fig. 38. ^{13}C NMR of compound **2q**

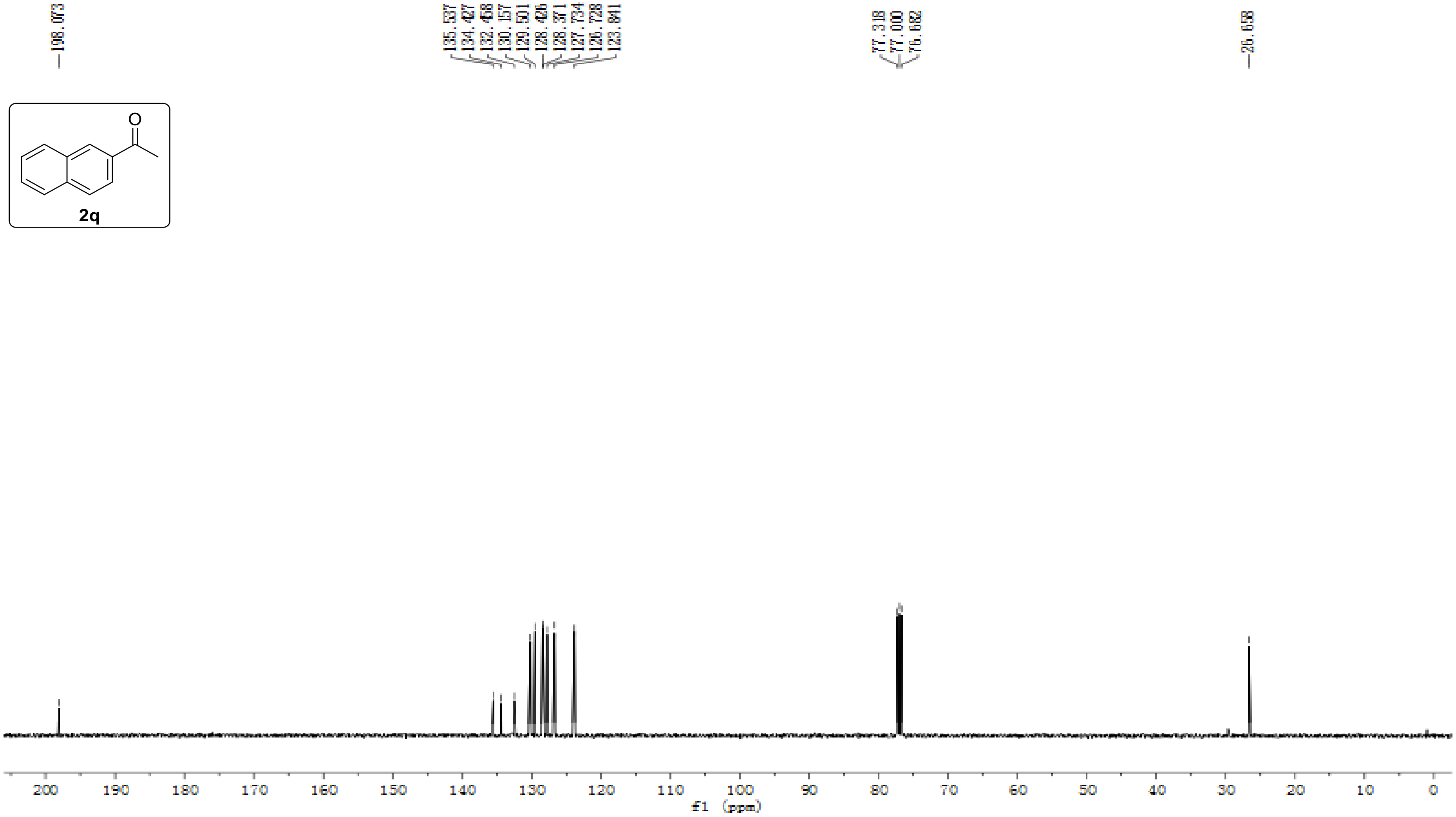

Supplementary Fig. 39. ^{1}H NMR of compound **2r**

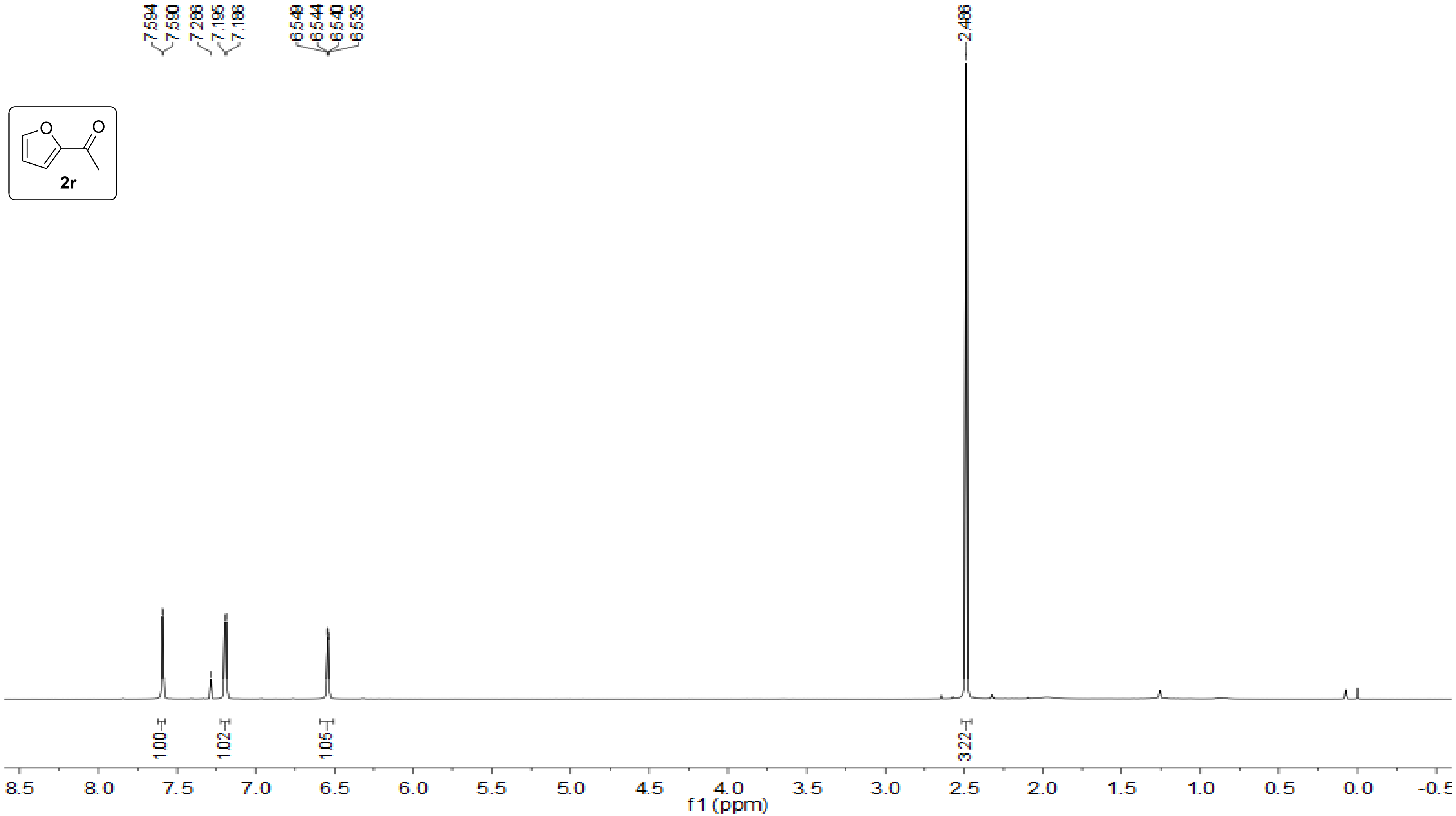

Supplementary Fig. 40. ^{13}C NMR of compound **2r**

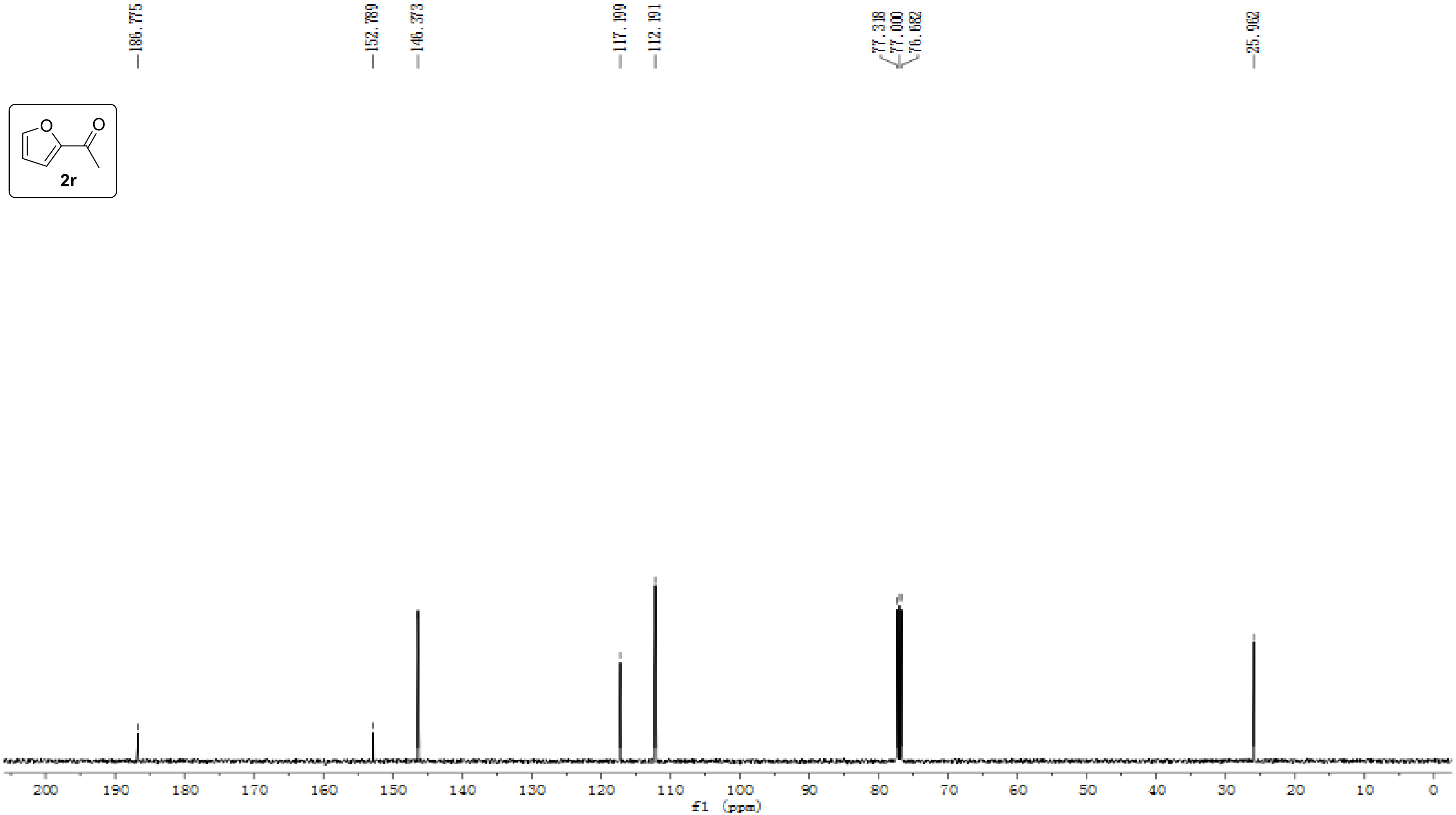

Supplementary Fig. 41.^{1}H NMR of compound **2s**

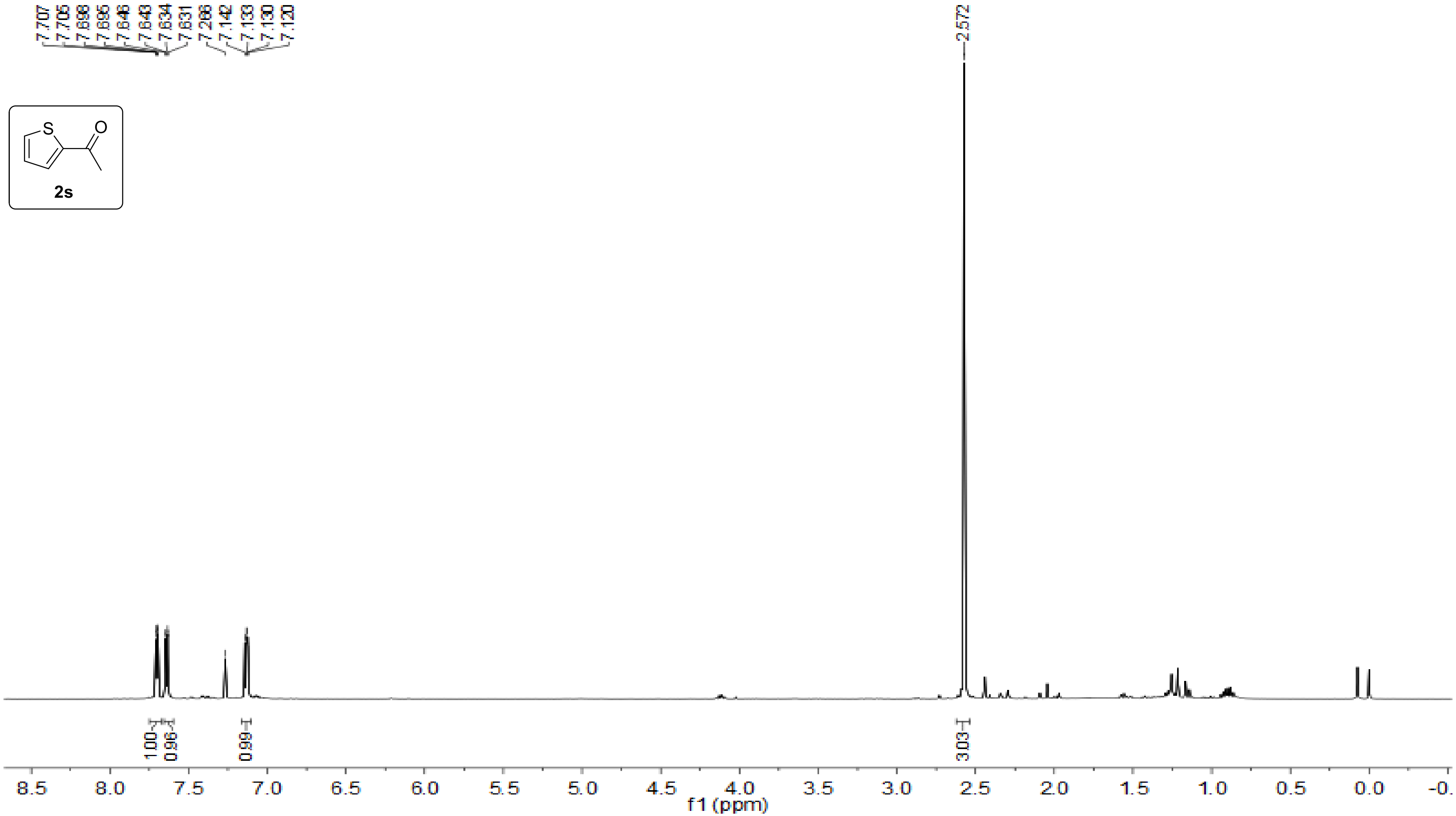

Supplementary Fig. 42. ^{13}C NMR of compound **2s**

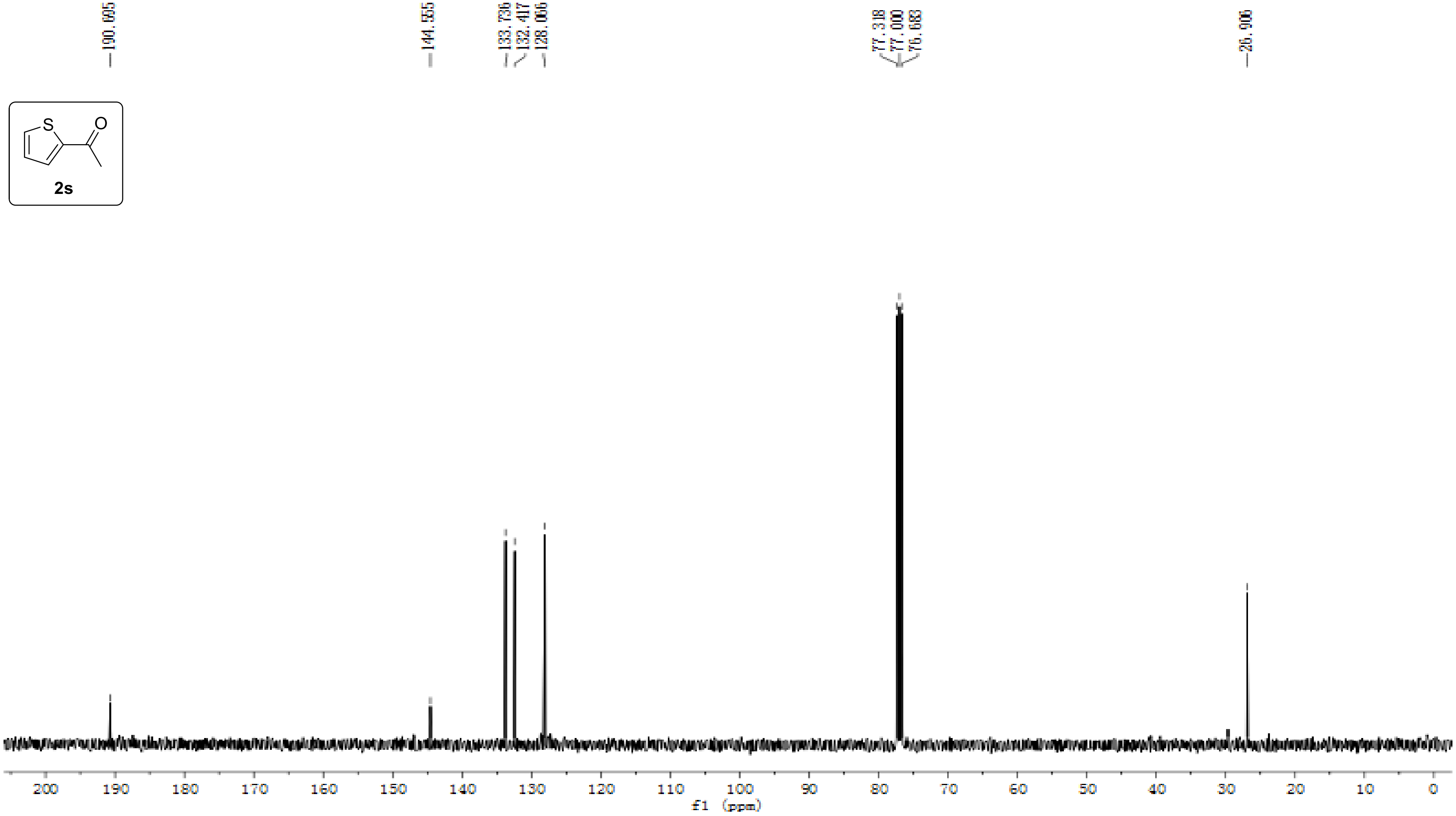

Supplementary Fig. 43. ^{1}H NMR of compound **2t**

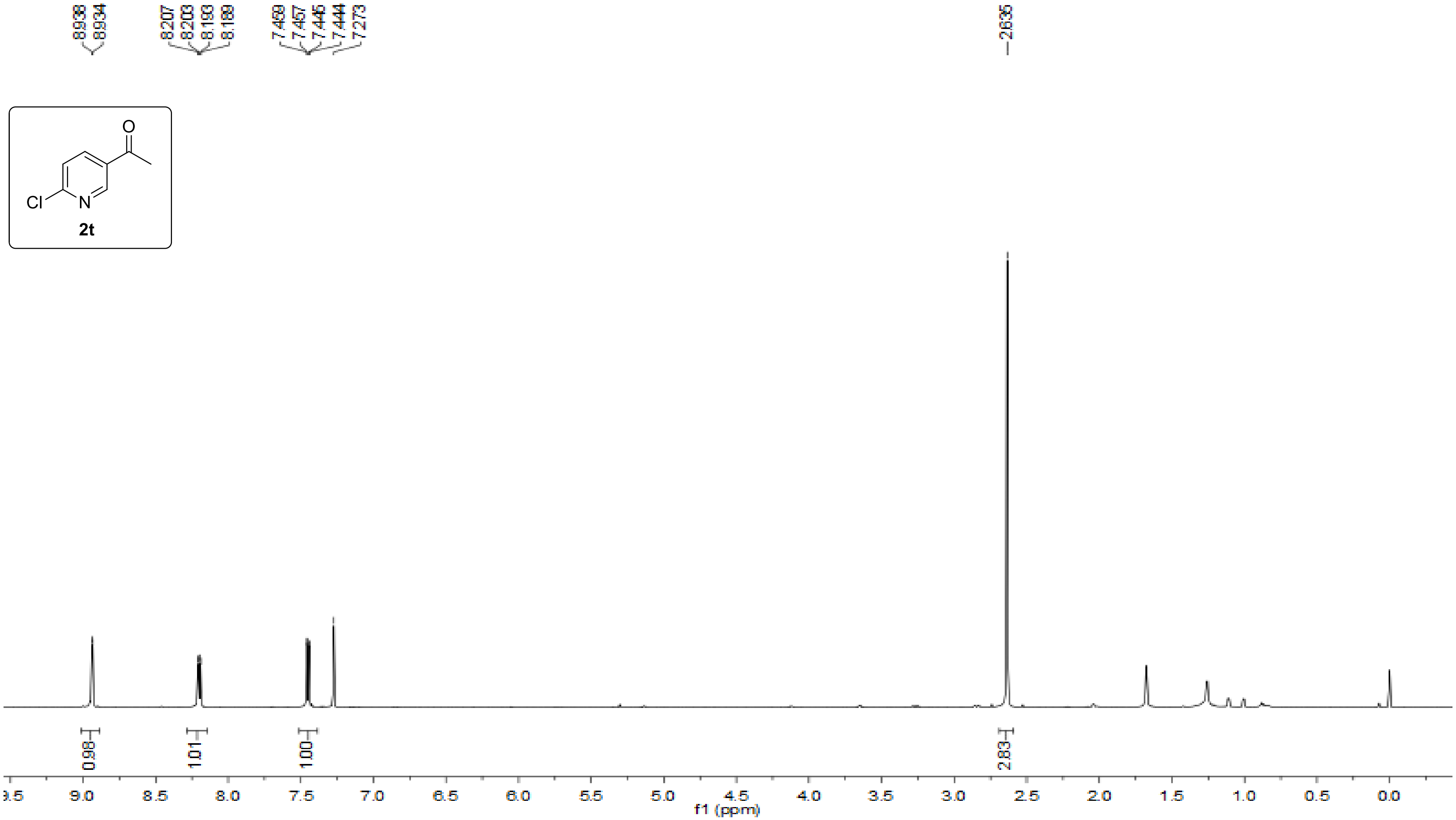

Supplementary Fig. 44. ^{13}C NMR of compound **2t**

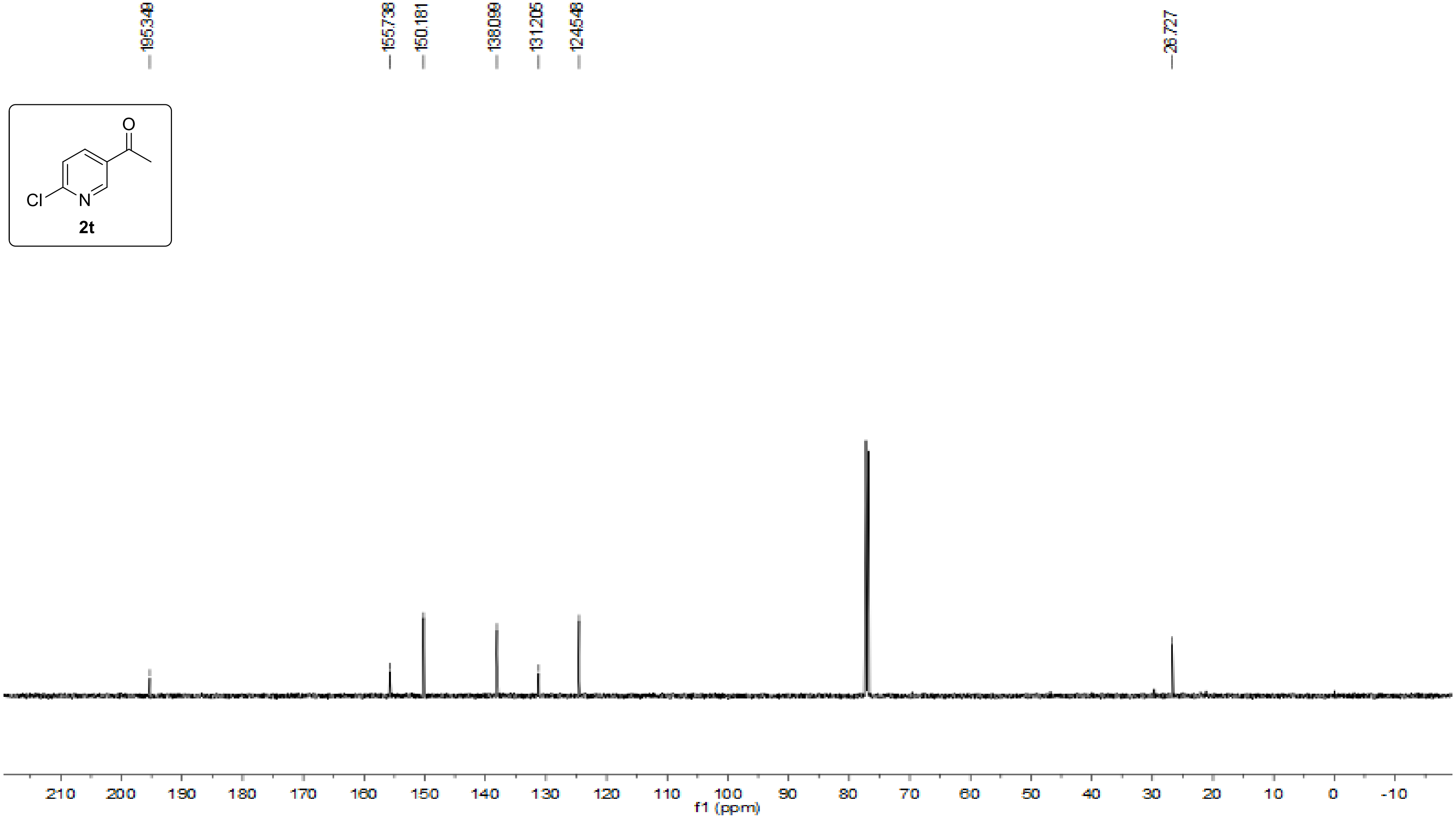

Supplementary Fig. 45. ^{1}H NMR of compound **2w**

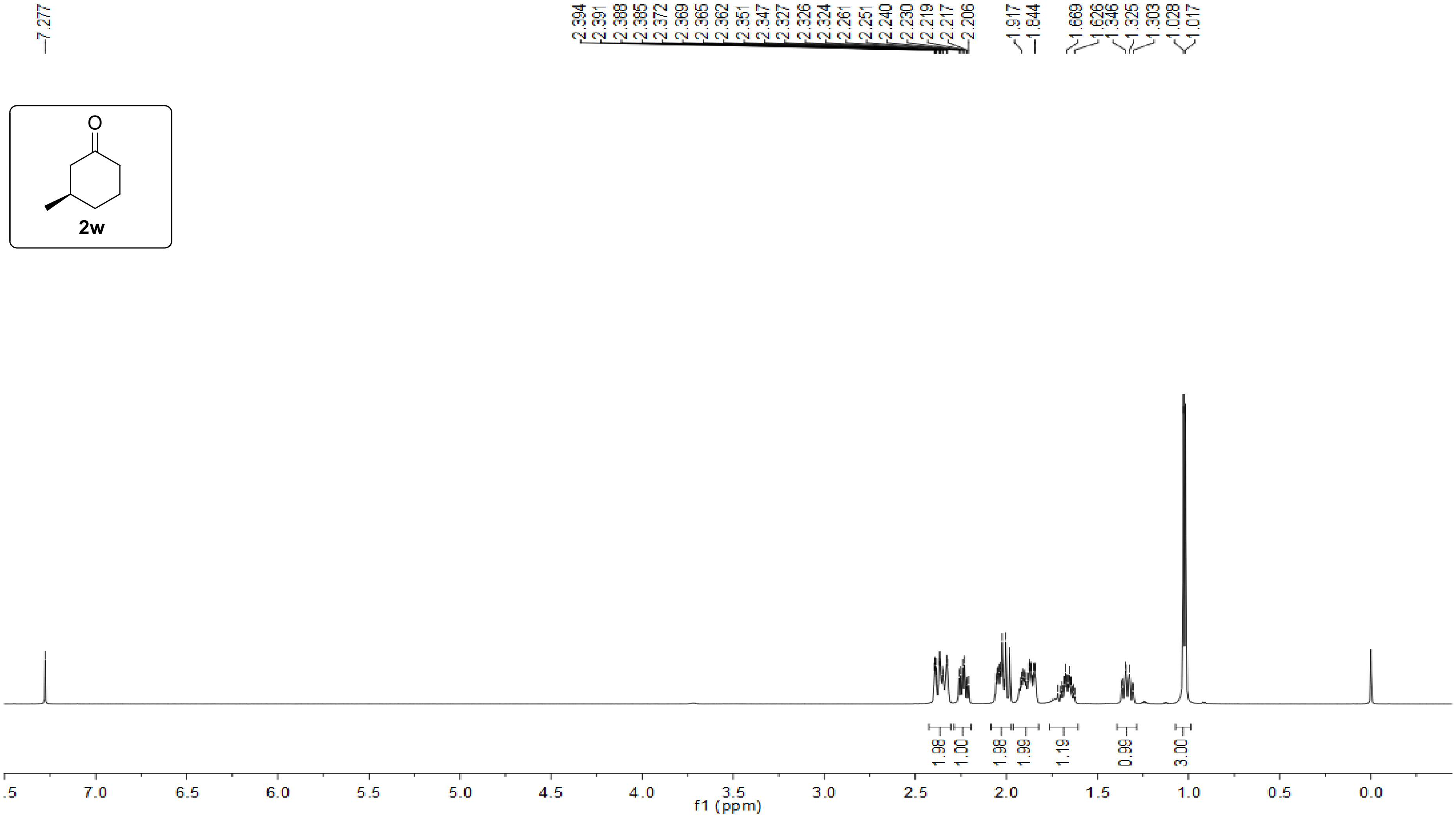

Supplementary Fig. 46.^{13}C NMR of compound **2w**

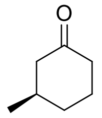
2w

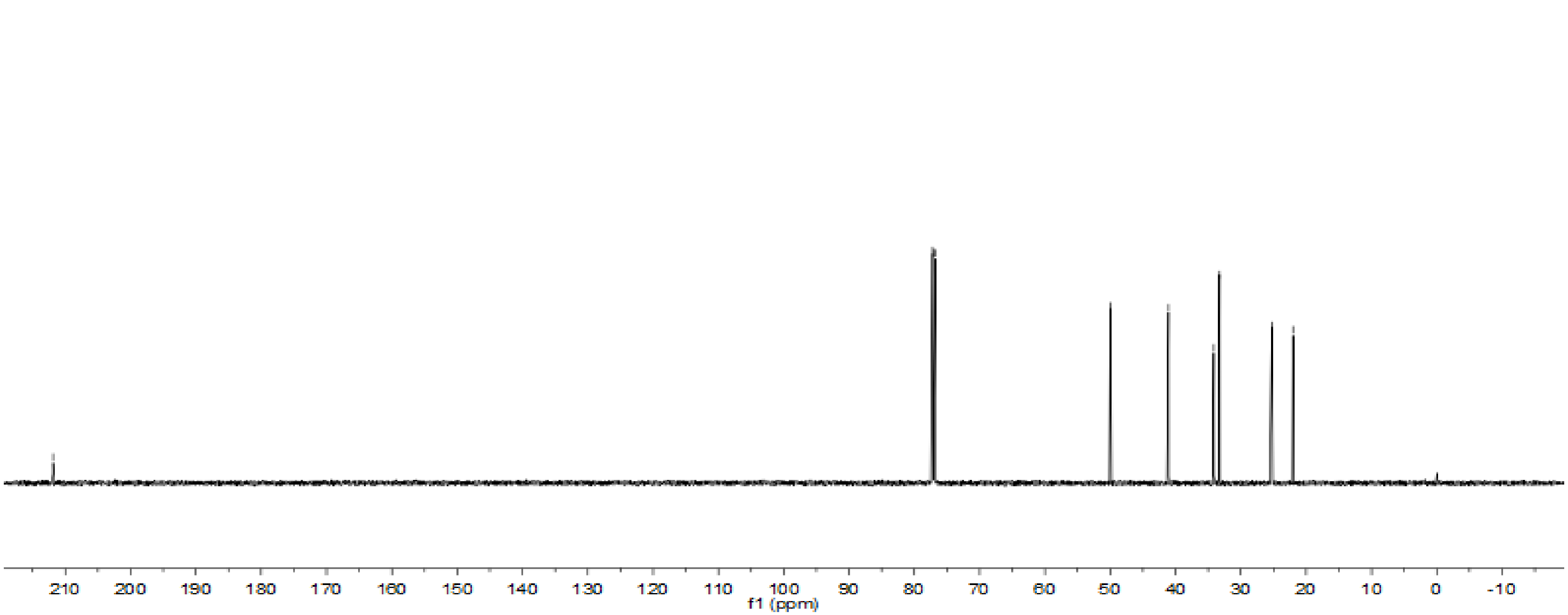

Supplementary Fig. 47. ^{1}H NMR of compound **2x**

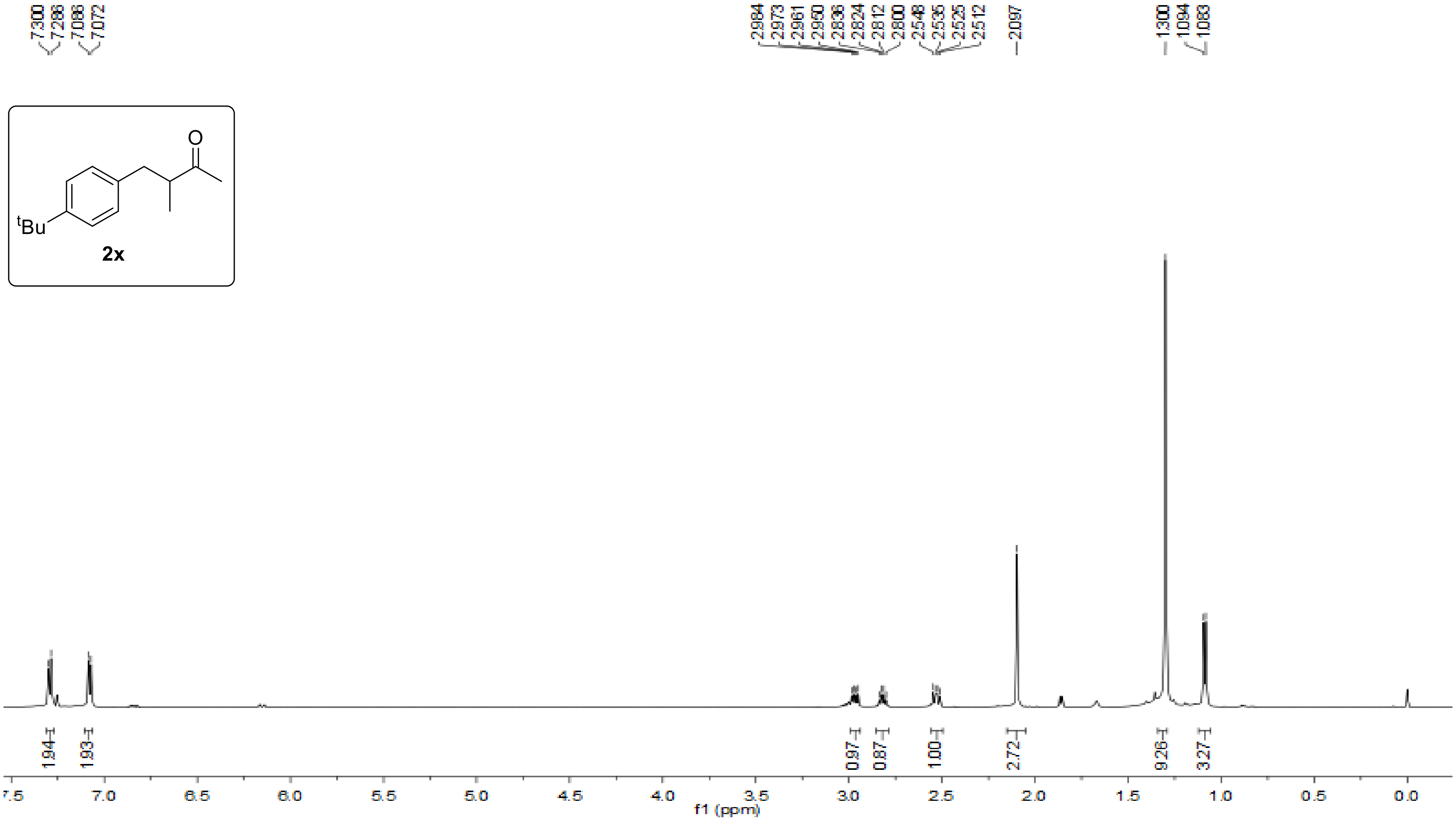

Supplementary Fig. 48.^{13}C NMR of compound **2x**

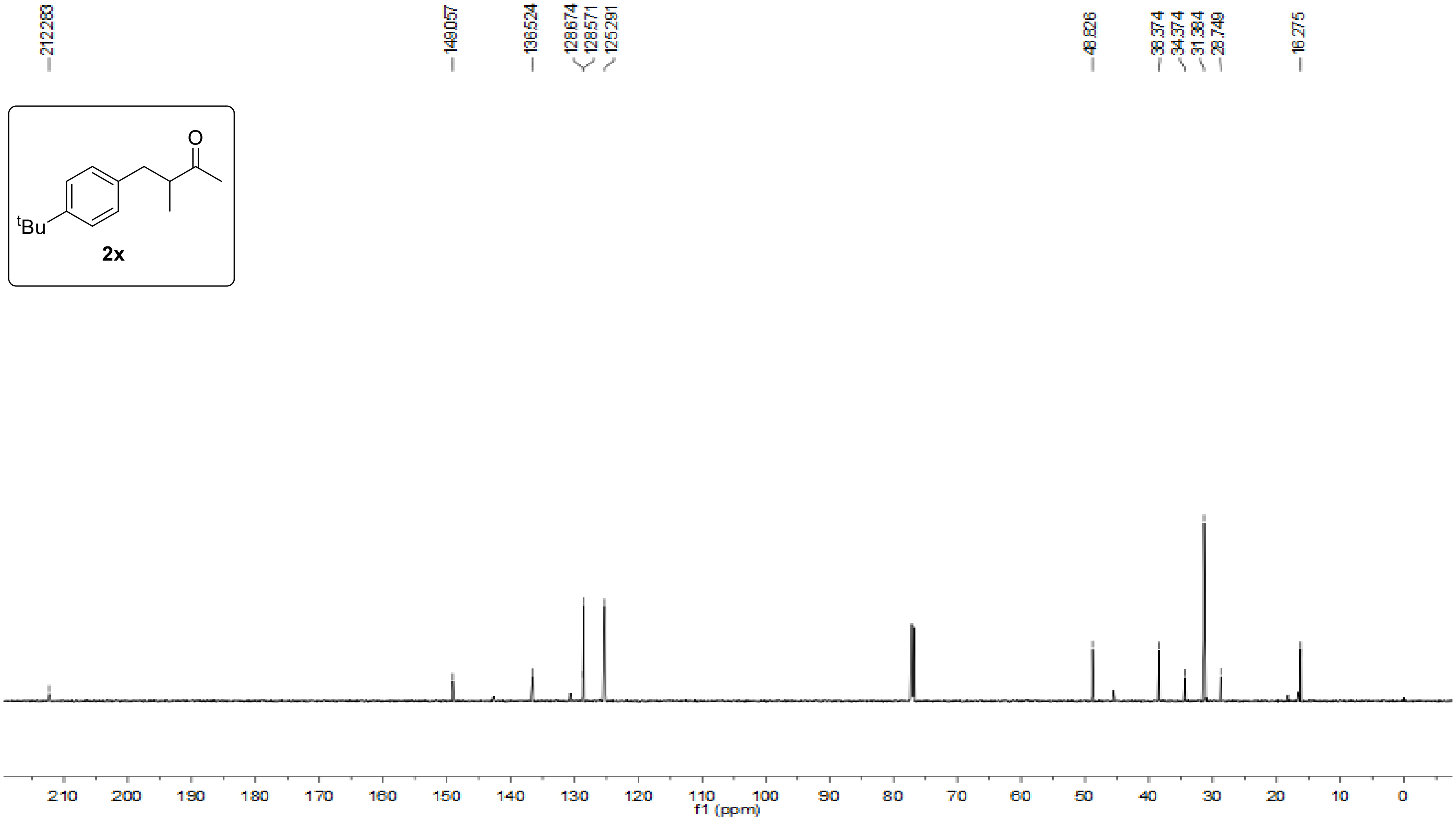

Supplementary Fig. 49.^{1}H NMR of compound **2y**

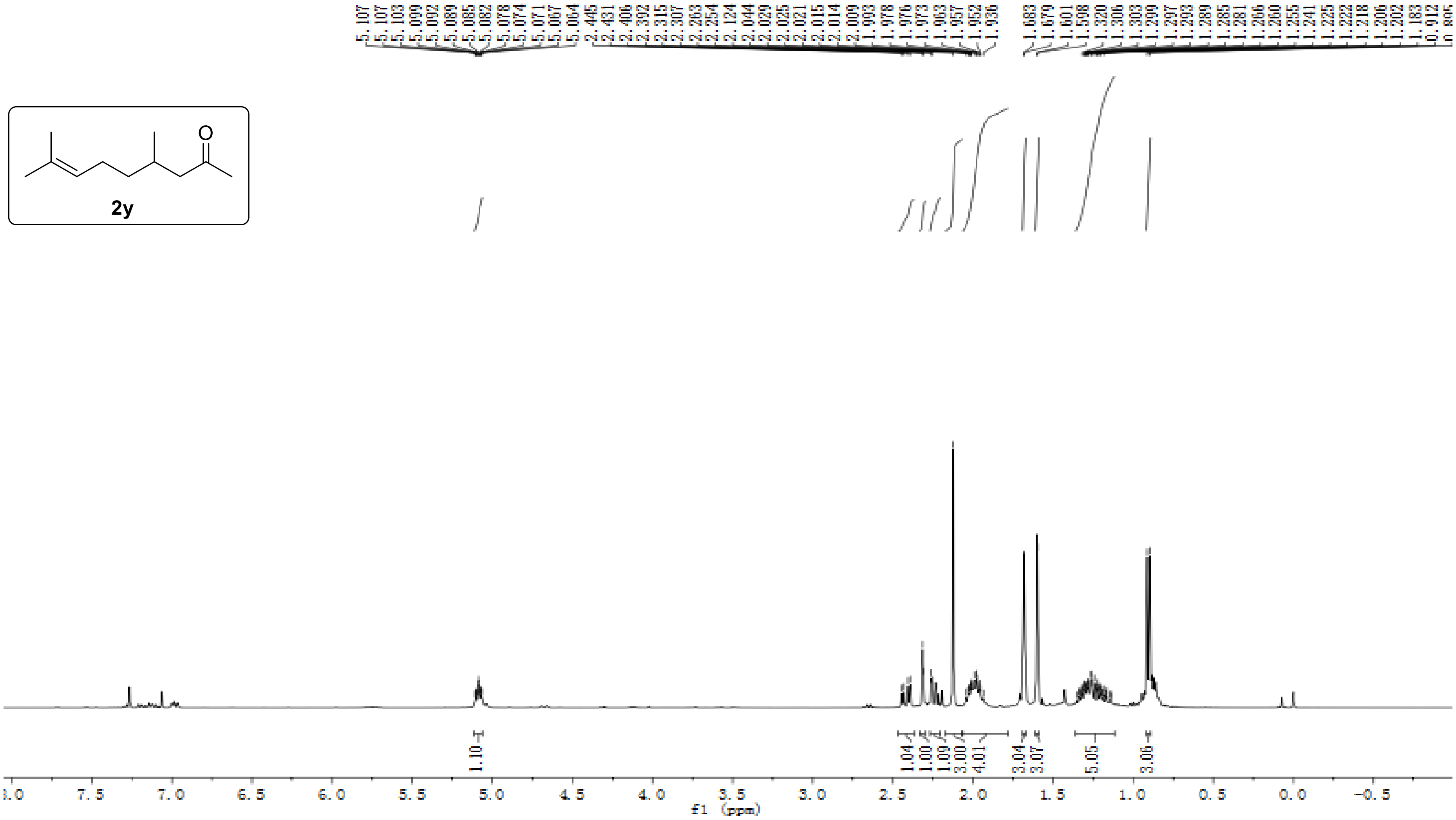

Supplementary Fig. 50. ^{13}C NMR of compound **2y**

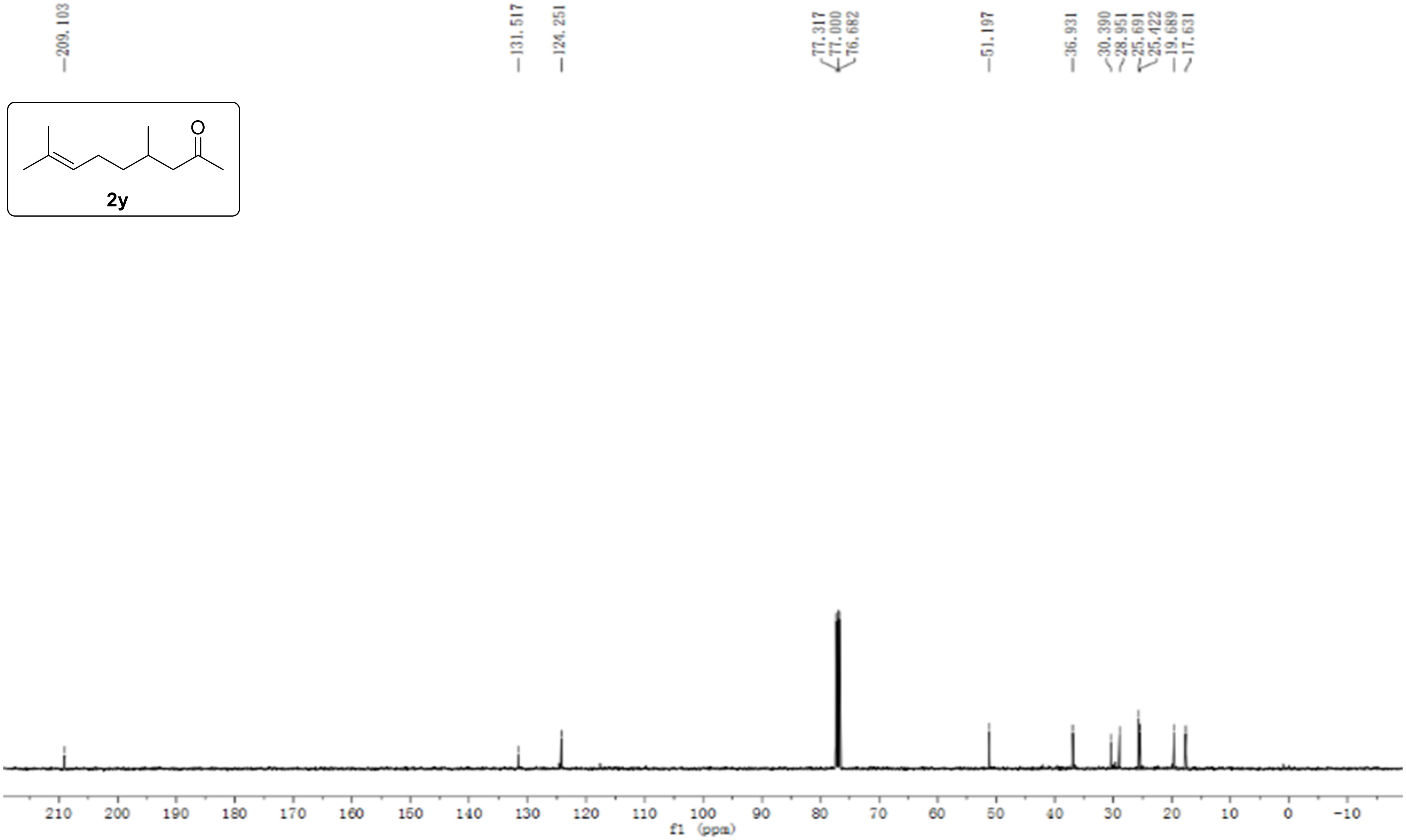

Supplementary Fig. 51.^{1}H NMR of compound **2z**

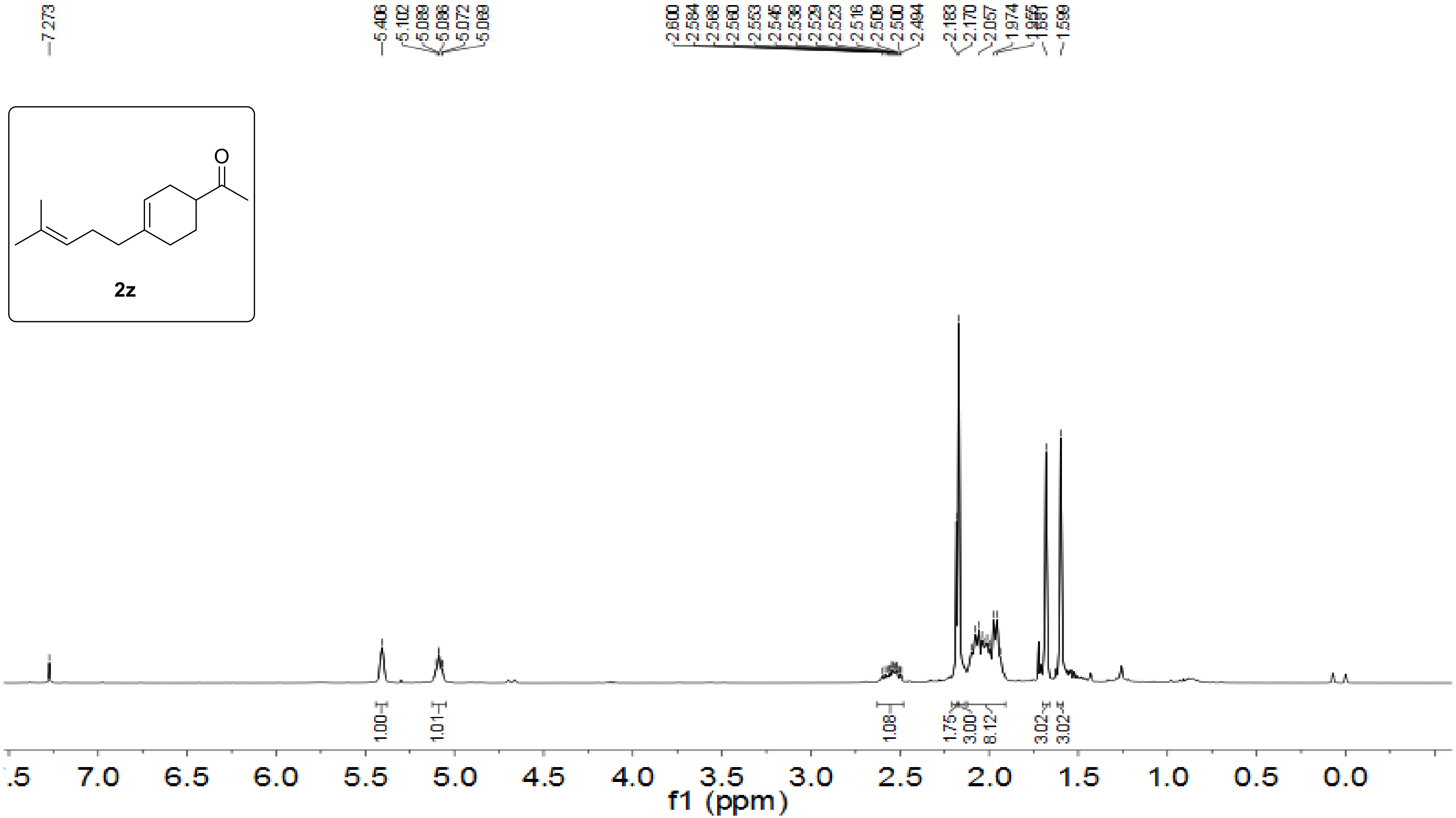

Supplementary Fig. 52. ^{13}C NMR of compound **2z**

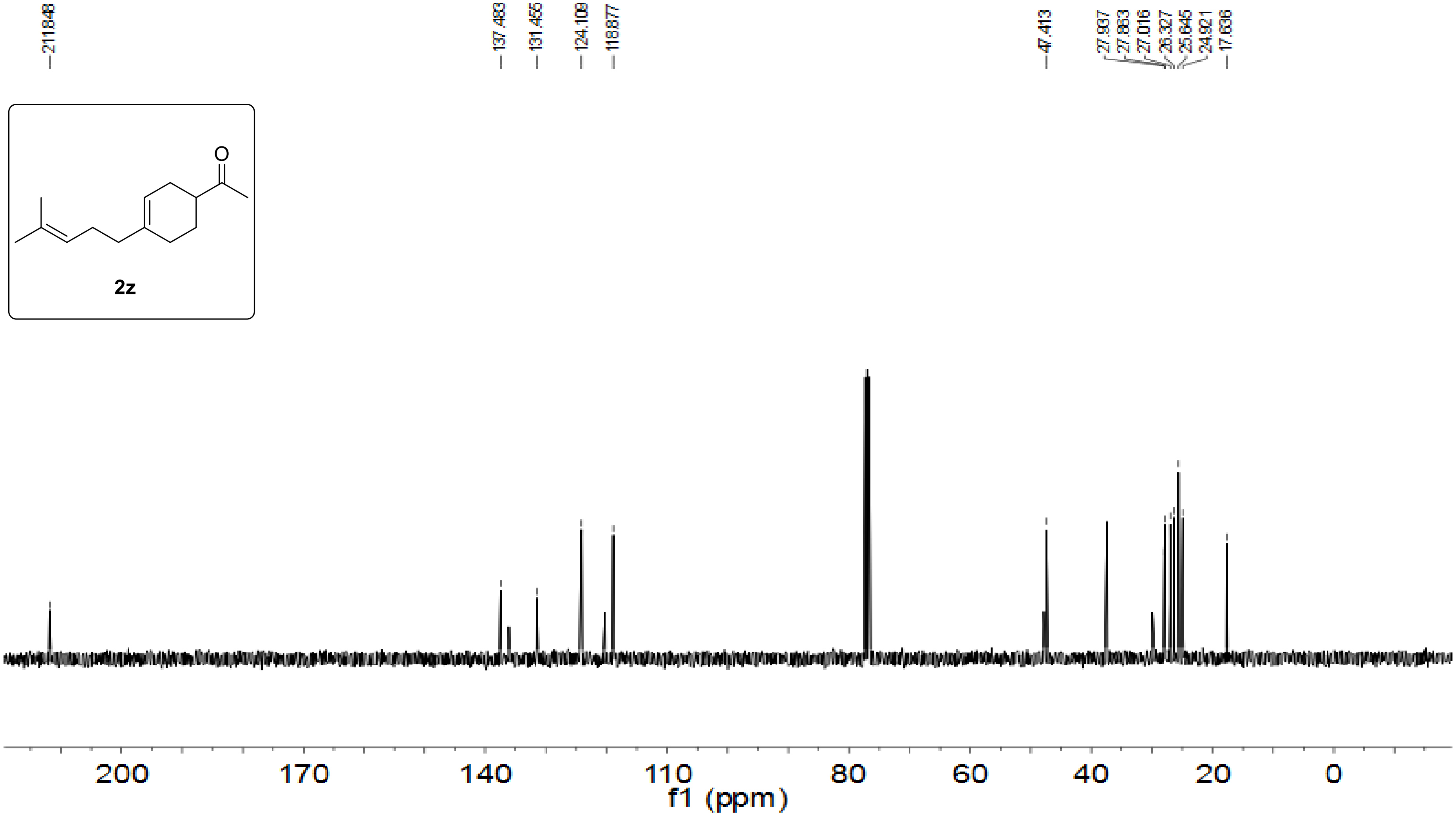

3. Supplementary References

1. Gaussian 09, Revision D.01, M. J. Frisch, G. W. Trucks, H. B. Schlegel, G. E. Scuseria, M. A. Robb, J. R. Cheeseman, G. Scalmani, V. Barone, G. A. Petersson, H. Nakatsuji, X. Li, M. Caricato, A. Marenich, J. Bloino, B. G. Janesko, R. Gomperts, B. Mennucci, H. P. Hratchian, J. V. Ortiz, A. F. Izmaylov, J. L. Sonnenberg, D. Williams-Young, F. Ding, F. Lipparini, F. Egidi, J. Goings, B. Peng, A. Petrone, T. Henderson, D. Ranasinghe, V. G. Zakrzewski, J. Gao, N. Rega, G. Zheng, W. Liang, M. Hada, M. Ehara, K. Toyota, R. Fukuda, J. Hasegawa, M. Ishida, T. Nakajima, Y. Honda, O. Kitao, H. Nakai, T. Vreven, K. Throssell, J. A. Montgomery, Jr., J. E. Peralta, F. Ogliaro, M. Bearpark, J. J. Heyd, E. Brothers, K. N. Kudin, V. N. Staroverov, T. Keith, R. Kobayashi, J. Normand, K. Raghavachari, A. Rendell, J. C. Burant, S. S. Iyengar, J. Tomasi, M. Cossi, J. M. Millam, M. Klene, C. Adamo, R. Cammi, J. W. Ochterski, R. L. Martin, K. Morokuma, O. Farkas, J. B. Foresman, and D. J. Fox, Gaussian, Inc., Wallingford CT, 2016..

2. A. D. Becke, Density-functional thermochemistry. III. The role of exact exchange. *J. Chem. Phys.* **98**, 5648-5652 (1993). DOI:10.1063/1.464913

3. C. Lee, W. Yang and R. G. Parr, Development of the Colle-Salvetti correlation-energy formula into a functional of the electron density. *Phys. Rev. B* **37**, 785-789 (1988). DOI:10.1103/PhysRevB.37.785

4. S. Grimme, J. Antony, S. Ehrlich and H. Krieg, A consistent and accurate *ab initio* parametrization of density functional dispersion correction (DFT-D) for the 94 elements H-Pu. *J. Chem. Phys.* **132**, 154104 (2010). DOI:10.1063/1.3382344

5. A. V. Marenich, C. J. Cramer and D. G. Truhlar, Universal solvation model based on solute electron density and on a continuum model of the solvent defined by the bulk dielectric constant and atomic surface tensions. *J. Phys. Chem. B* **113**, 6378-6396 (2009). DOI:10.1021/jp810292n

6. K. Raghavachari, J. S. Binkley, R. Seeger, and J. A. Pople, Self-Consistent Molecular Orbital Methods. 20. Basis set for correlated wave-functions, *J. Chem. Phys.*, **72**, 650-654 (1980). DOI: 10.1063/1.438955

7. T. Clark, J. Chandrasekhar, G. W. Spitznagel, and P. v. R. Schleyer, Efficient diffuse function-augmented basis-sets for anion calculations. 3. The 3-21+G basis set for 1st-row elements, Li-F, *J. Comp. Chem.*, **4**, 294-301 (1983). DOI: 10.1002/jcc.540040303

8. M. J. Frisch, J. A. Pople, and J. S. Binkley, Self-Consistent Molecular Orbital Methods. 25. Supplementary Functions for Gaussian Basis Sets *J. Chem. Phys.*, **80**, 3265-3269 (1984). DOI: 10.1063/1.447079

9. T. H. Dunning Jr. and P. J. Hay, in Modern Theoretical Chemistry, Ed. H.F. Schaefer III, Vol. 3 (Plenum, New York, 1977) 1-28.

10. D. Andrae, U. Haeussermann, M. Dolg, H. Stoll, and H. Preuss, Energy-adjusted ab initio pseudopotentials for the 2nd and 3rd row transition-elements, *Theor. Chem. Acc.*, **77**, 123-141 (1990). DOI: 10.1007/BF01114537.
