# Supplementary material for: C(alkyl)–C(vinyl) bond cleavage enabled by Retro-Pallada-Diels-Alder reaction
Source: Nat Commun. 2023 May 4;14:2572. doi: 10.1038/s41467-023-38067-7 (PMC10160084; doi:10.1038/s41467-023-38067-7)
Supplement: Supplementary file 3 — Description of Additional Supplementary Files [file 41467_2023_38067_MOESM3_ESM.docx]

**File Name**: Supplementary Data 1
**Description**: The computationally derived energies and Cartesian coordinates
