# Supplementary material for: C(alkyl)–C(vinyl) bond cleavage enabled by Retro-Pallada-Diels-Alder reaction
Source: Nat Commun. 2023 May 4;14:2572. doi: 10.1038/s41467-023-38067-7 (PMC10160084; doi:10.1038/s41467-023-38067-7)
Supplement: Supplementary file 4 — Supplementary Data 1 [file 41467_2023_38067_MOESM4_ESM.docx]

The potential energies, free energies (in hatree) and their relative values with respect to **Im1** (in kcal/mol) calculated by B3LYP-D3/6-311++G(d,p)//SDD in dichloroethane.

|  | Energy | Gibbs | ΔE | ΔG |
| --- | --- | --- | --- | --- |
| **Im1** | -910.5759860 | -910.300890 | 0.00 | 0.00 |
| **TS1** | -910.5722962 | -910.298083 | 2.32 | 1.76 |
| **Im2** | -910.6051755 | -910.325058 | -18.32 | -15.17 |
| **I-Im3** | -910.5491616 | -910.282457 | 16.83 | 11.57 |
| **I-Im4** | -910.5362087 | -910.281067 | 1.10 | 2.80 |
| **I-Im5** | -896.4637667 | -896.202486 | 24.96 | 12.44 |
| **I-TS2** | -896.4769879 | -896.217704 | 78.06 | 60.98 |
| **I-Im6** | -896.4472474 | -896.209735 | 69.77 | 51.43 |
| **Ⅱ-Im6** | -1048.1764170 | -1047.882840 | 14.80 | 15.55 |
| **Ⅱ-TS2** | -1048.1354030 | -1047.843029 | 40.54 | 40.53 |
| **Ⅱ-Im7** | -972.3916295 | -972.112288 | 25.55 | 11.96 |
| **Ⅱ-Im8** | -1048.1764540 | -1047.885376 | 14.78 | 13.96 |
| **Ⅱ-TS3** | -1048.1769170 | -1047.885132 | 14.48 | 14.11 |
| **Ⅱ-Im9** | -934.8484049 | -934.560107 | 5.06 | -6.80 |
| **Ⅱ-TS4** | -934.7889253 | -934.506961 | 42.38 | 28.94 |
| **Ⅱ-Im10** | -934.8083811 | -934.529344 | 30.17 | 13.88 |
| **Ⅲ-Im3** | -1062.226189 | -1061.920888 | -24.08 | -7.56 |
| **Ⅲ-Im4** | -948.8605429 | -948.560197 | -10.21 | -5.64 |
| **Ⅲ-TS2** | -948.8261892 | -948.528814 | 11.35 | 14.05 |
| **Ⅲ-Im5** | -948.8989114 | -948.602424 | -43.29 | -32.14 |
| **Ⅲ-Im6** | -948.8902153 | -948.612020 | -28.83 | -38.16 |
| **Ⅲ-TS3** | -948.8372204 | -948.564952 | 4.43 | -8.62 |
| **Ⅲ-Im7** | -948.8569674 | -948.587245 | -7.97 | -22.61 |
| **Int C** | -576.5535599 | -576.341020 |  |  |
| **Ⅲ-Im8** | -295.8020814 | -295.773465 | 13.82 | -16.67 |
| **Ⅲ-TS4** | -295.7965590 | -295.767520 | 17.28 | -12.94 |
| **Ⅲ-Im9** | -295.8614275 | -295.826982 | -23.42 | -50.26 |
| **Ⅲ-TS5** | -295.8399736 | -295.809407 | -9.96 | -39.23 |
| **Ⅲ-Im10** | -295.8500142 | -295.818577 | -16.26 | -44.98 |
| **Ⅲ-Im11** | -153.8727400 | -153.841684 | 12.45 | -29.46 |
| **PdBr_2_** | -154.7364403 | -154.767025 | 44.69 | 24.55 |
| **Im-a** | -462.6129289 | -462.536301 | -14.29 | -8.35 |
| **TS-a** | -462.5804174 | -462.510724 | 6.11 | 7.70 |
| **Im-b** | -462.5917172 | -462.526613 | -0.98 | -2.27 |
| **HPd(CO)Br** | -462.5471599 | -462.506782 | 26.28 | 10.17 |
| **Im-a’** | -349.2167387 | -349.145860 | 18.75 | 12.24 |
| **TS-a’** | -349.1769011 | -349.110474 | 43.75 | 34.44 |
| **Im-b’** | -349.1844174 | -349.120033 | 39.03 | 28.44 |
| **TS-b’** | -349.1838363 | -349.127060 | 39.39 | 24.03 |
| **Im-c’** | -349.1943827 | -349.138858 | 32.78 | 16.63 |
| **HPdBr** | -141.9315200 | -141.952152 | 51.46 | 22.86 |
| **Im1’** | -797.2171146 | -796.943634 | 9.82 | 1.33 |
| **TS1’** | -797.2017314 | -796.929642 | 19.47 | 9.00 |
| **Im2’** | -797.2360753 | -796.959728 | -2.08 | -9.88 |
| **iPrOH** | -194.4389568 | -194.359214 |  |  |
| **Acetone-HBr** | -207.233084 | -207.176781 |  |  |
| **Acetone** | -193.2320462 | -193.178348 |  |  |
| **HBr** | -13.987815 | -14.001225 |  |  |
| **CO** | -113.3435367 | -113.357628 |  |  |
| **H_2_O_2_** | -151.6118288 | -151.607953 |  |  |

**Im1**

C -5.61189800 -0.48100700 0.66942800

C -4.94357100 -1.35621600 -0.18462800

C -3.67788700 -1.03387600 -0.67370400

C -3.05040800 0.16859900 -0.31140000

C -3.73748600 1.04310000 0.54954100

C -5.00061400 0.72144900 1.03385700

H -6.59809600 -0.72963800 1.04562900

H -5.40391100 -2.29573300 -0.47057000

H -3.17532300 -1.74059000 -1.32279600

H -3.27204700 1.97953400 0.83048500

H -5.51418000 1.41336200 1.69278100

C -1.69096700 0.50724500 -0.80175000

C -1.13674900 -0.16502200 -2.04418800

H -1.95806400 -0.56557100 -2.64387300

H -0.62588100 0.55980000 -2.68580700

H 0.36495700 1.76709800 -1.50637600

N -1.00374800 1.33695100 -0.09937000

N 0.27916200 1.64360400 -0.49671500

H 2.43932700 0.03738300 -1.72748100

C -0.21128400 -1.31911300 -1.72258100

H -0.58141400 -2.02549000 -0.98663900

Pd 1.74489500 -0.63557300 -0.53607500

C 0.87487700 -1.66313600 -2.47428800

H 1.16081900 -1.07816900 -3.34205800

H 1.34799500 -2.63263600 -2.36173800

C 0.85290600 2.82920800 0.20810300

C 1.04044200 2.47972200 1.68681800

H 0.08418200 2.23514700 2.15360200

H 1.70143300 1.61662300 1.79852200

H 1.47850500 3.32908000 2.21789800

C 2.20616400 3.11814600 -0.45062900

H 2.65735600 4.00164700 0.00703500

H 2.89679600 2.28166400 -0.32986100

H 2.09039500 3.31772400 -1.52142000

C -0.07053100 4.04891500 0.04865700

H 0.37173600 4.92471700 0.53143000

H -0.22273100 4.28182000 -1.01035300

H -1.04515100 3.86061900 0.50333500

C 3.41697900 -0.42578700 0.36173700

Br 0.73838700 -1.84103400 1.57821100

O 4.40085800 -0.32038700 0.91913300

**TS1**

C 5.48429900 -0.29314000 -0.57900500

C 4.82371900 -1.18576000 0.26344300

C 3.54382000 -0.89412100 0.73405700

C 2.89351000 0.29449000 0.36705600

C 3.57264900 1.18648900 -0.48202400

C 4.84964400 0.89536800 -0.94952800

H 6.48117100 -0.51790100 -0.94196700

H 5.30119300 -2.11579000 0.55272500

H 3.04489800 -1.61487800 1.37074000

H 3.08900600 2.11208200 -0.76849400

H 5.35575300 1.60051200 -1.60033100

C 1.52097700 0.59374900 0.83849200

C 0.97709400 -0.06443400 2.09197400

H 1.80567800 -0.44635100 2.69371700

H 0.48976500 0.69479000 2.71397200

H -0.61759300 1.73298200 1.51590300

N 0.82175900 1.41901400 0.14022700

N -0.47104500 1.67169500 0.50780200

H -2.46406000 -0.63505400 1.79079300

C 0.01523900 -1.22446700 1.88626300

H 0.43175100 -2.11499200 1.42709000

Pd -1.67246600 -0.87134300 0.45862600

C -1.13771900 -1.35017500 2.66005800

H -1.36617300 -0.59504500 3.40667900

H -1.59117800 -2.32237000 2.81920200

C -1.10886000 2.81931600 -0.18895400

C -1.19315100 2.50387900 -1.68540200

H -0.19720200 2.37361100 -2.11278000

H -1.75457700 1.58148300 -1.85284700

H -1.69331400 3.32174700 -2.21119600

C -2.51478200 2.94860400 0.40801400

H -3.04288400 3.78402500 -0.05750900

H -3.09665700 2.03854500 0.24323000

H -2.47016100 3.13870000 1.48596600

C -0.31349900 4.11345800 0.05180800

H -0.80057500 4.96029400 -0.43995600

H -0.24675400 4.33113700 1.12283000

H 0.70024600 4.02188200 -0.34469800

Br -0.24254500 -1.70742300 -1.56261100

C -3.25688800 -0.70590100 -0.63225000

O -4.16589300 -0.61836300 -1.30733700

**Im2**

C 5.60381000 -1.35944900 -1.05917400

C 4.71012400 -2.26096400 -0.48632900

C 3.51158000 -1.80778100 0.05797800

C 3.18276800 -0.44579400 0.02422200

C 4.08671700 0.45197400 -0.56348400

C 5.28748700 -0.00084400 -1.09528200

H 6.54170600 -1.71211200 -1.47560000

H 4.94306600 -3.32038200 -0.46451300

H 2.81924800 -2.52623700 0.48267900

H 3.83615500 1.50553800 -0.59371800

H 5.98109400 0.70675600 -1.53784000

C 1.88919800 0.03341900 0.56308600

C 1.17607200 -0.69634000 1.67087700

H 1.81594500 -1.47782100 2.08851800

H 1.03204300 0.00960200 2.50407800

H 0.12130600 1.41385800 1.57096200

N 1.39260700 1.09515000 0.03626800

N 0.12799200 1.50361400 0.55182700

H -1.22824000 -0.82386200 3.14365300

C -0.19008800 -1.30648800 1.28590400

H -0.01933000 -2.18285500 0.65620000

C -0.96354200 -1.69950200 2.53917600

H -0.35963900 -2.35868800 3.18029400

H -1.88253000 -2.22784700 2.29090300

C -0.09731200 2.97666500 0.27227600

C -0.09825700 3.22629500 -1.23484000

H 0.84629900 2.91407800 -1.67887400

H -0.90242500 2.68638300 -1.73355300

H -0.24052300 4.29198100 -1.42851500

C -1.43509500 3.36138900 0.90996500

H -2.27396300 2.83440100 0.45606800

H -1.44465200 3.14696200 1.98361400

H -1.60567800 4.43299400 0.78847600

C 1.03759500 3.77234800 0.93964400

H 0.88430300 4.84326300 0.78933400

H 1.06847800 3.59012700 2.01913000

H 2.00556300 3.50068200 0.51831800

Pd -1.33120000 -0.08202400 0.01629800

Br -2.72423200 -2.13022000 -0.51434300

C -2.57508100 0.86449100 -1.25730100

O -3.34436600 1.27951900 -1.97803500

**I-Im3**

C 5.50773400 0.15444300 -0.88139000

C 4.92237800 -1.08632300 -0.63233400

C 3.61364600 -1.16179200 -0.15993700

C 2.86528400 0.00529000 0.05332600

C 3.46417000 1.25036800 -0.20071800

C 4.77473200 1.32257400 -0.66044600

H 6.52904900 0.21225000 -1.24085300

H 5.48326600 -1.99786900 -0.80551800

H 3.17367300 -2.13522100 0.02029200

H 2.89875900 2.15727400 -0.02611600

H 5.22789600 2.29071200 -0.84242400

C 1.45489000 -0.06752200 0.50142400

C 0.94172500 -1.22593400 1.32912300

H 1.75245400 -1.92183300 1.55685700

H 0.59448500 -0.84763700 2.29929300

H -0.68651400 0.54978400 1.58821600

N 0.67588600 0.88815600 0.14329800

N -0.67446200 0.79956400 0.59374100

H -1.20924300 -2.50019900 2.48530100

C -0.17803400 -1.99067700 0.64990600

H 0.15602100 -2.44226200 -0.29852200

C -0.96536100 -2.93677300 1.51428700

H -0.38960000 -3.85841400 1.68317900

H -1.89189500 -3.26409000 1.01747500

C -1.35376400 2.16073700 0.51570900

C -1.42794000 2.62281600 -0.93690400

H -0.43026200 2.73126100 -1.36520500

H -1.99466900 1.92201800 -1.55241500

H -1.92896300 3.59270800 -0.97845700

C -2.74426100 2.00789300 1.13825000

H -3.39133200 1.35450000 0.55158900

H -2.67848200 1.60928400 2.15576800

H -3.21990100 2.98920400 1.19389700

C -0.52778800 3.14819500 1.35845300

H -1.03089300 4.11757200 1.37498700

H -0.43342800 2.79563200 2.39037000

H 0.47156400 3.28422100 0.94336500

Pd -1.66757900 -0.96497100 -0.28877800

C -3.24513200 -0.36569700 -1.43278900

O -4.11462600 -0.13623000 -2.12028700

**I-Im4**

C -5.92794600 -0.24877600 0.42230800

C -5.30051600 -1.24137600 -0.33002100

C -3.92432200 -1.19475300 -0.54536200

C -3.15071300 -0.16209000 0.00504100

C -3.79318000 0.83370900 0.75828000

C -5.16896500 0.79118700 0.96255500

H -7.00007100 -0.28132300 0.58167400

H -5.88162600 -2.05412500 -0.75147900

H -3.45650400 -1.98064200 -1.12615200

H -3.20619900 1.64500000 1.17027700

H -5.65103700 1.57378400 1.53806200

C -1.67414400 -0.12397700 -0.17042600

C -1.02574700 -0.71587000 -1.40721500

H -1.79434000 -1.07470000 -2.09669300

H -0.44495100 0.04230600 -1.94271300

H 0.61819600 1.06450900 -0.17029100

N -1.00659200 0.36862000 0.80650400

N 0.40786600 0.49995200 0.68164000

H 0.94873000 -2.03970900 -2.85476900

C -0.15340000 -1.87349200 -0.98796300

H -0.63829200 -2.53943100 -0.26802200

C 0.78134100 -2.55059800 -1.90761300

H 0.58045700 -3.61659800 -2.03946500

H 1.82838200 -2.61357000 -1.39584900

C 0.92791100 1.28917800 1.87786200

C 0.90143100 0.38590000 3.11011100

H -0.10781100 0.00860000 3.29254300

H 1.57490000 -0.46691200 2.98633300

H 1.22095900 0.94971400 3.99032500

C 2.34825000 1.76863100 1.56176700

H 3.04616200 0.94602800 1.41529400

H 2.36087500 2.38650400 0.66115700

H 2.71333300 2.36404700 2.40185600

C 0.04312900 2.53161600 2.09490500

H 0.50324500 3.16758300 2.85539100

H -0.04189600 3.11081000 1.17120100

H -0.95741800 2.26133600 2.42972900

Pd 1.53552500 -1.33553100 0.04153500

C 3.32647800 -1.39032700 0.94167600

O 4.31179600 -1.60467500 1.46133300

Br 1.24542400 2.19270900 -2.11948900

**I-Im5**

C -5.68675300 -0.50554400 -0.14418300

C -4.92827400 -1.62805800 -0.46895600

C -3.53380300 -1.57558400 -0.43606000

C -2.85622600 -0.39427900 -0.07802000

C -3.64217800 0.73269600 0.24624800

C -5.02961800 0.67698700 0.21370900

H -6.77014000 -0.54706000 -0.16739500

H -5.42008600 -2.55160600 -0.75629900

H -2.97443700 -2.46049600 -0.71483700

H -3.14540800 1.65141400 0.53181600

H -5.60591000 1.55834500 0.47610000

C -1.38001800 -0.32998600 -0.05572200

C -0.57664700 -1.61360400 0.00824700

H -1.20766900 -2.37145500 0.49024700

H -0.34483700 -2.01505100 -0.98942600

N -0.85315700 0.85776300 -0.10310200

C -0.06151200 3.46461400 -0.71050200

H 0.03155000 3.25216600 -1.77968600

H -1.11345800 3.38362500 -0.43540400

H 0.26580500 4.49363100 -0.53346600

C 1.29596000 -2.87151400 1.17096300

H 1.51311100 -3.45048400 0.26849900

H 2.20943900 -2.78055900 1.76271100

H 0.57552500 -3.45034500 1.76643200

C 0.69186200 -1.52075600 0.84682400

H 0.52384900 -0.93718600 1.75559200

C 3.46714100 -1.46349000 -0.36300000

O 4.38854000 -2.13291800 -0.41672600

C 0.80920600 2.49625500 0.10734900

C 0.61975600 2.74750600 1.61299300

H 1.26111200 2.07830900 2.19477000

H 0.87364400 3.78012300 1.87210600

H -0.41850300 2.56504000 1.90230200

C 2.28031600 2.71429000 -0.27274700

H 2.58021600 3.74024100 -0.04315800

H 2.94558200 2.04939500 0.29233500

H 2.43966000 2.54657400 -1.34269400

Pd 1.97136800 -0.27395700 -0.24001000

N 0.44950200 1.07845600 -0.21677100

**I-TS2**

C 5.37320000 -0.02309500 -0.61757000

C 4.96955900 -0.85158800 0.43029100

C 3.66689300 -0.78631200 0.91686100

C 2.73672100 0.10650200 0.35677600

C 3.15749700 0.94040500 -0.69431100

C 4.46178700 0.87495000 -1.17499500

H 6.38957000 -0.07293000 -0.99197400

H 5.67367400 -1.54438900 0.87764100

H 3.38292700 -1.41977400 1.74827100

H 2.45555400 1.63459200 -1.13940400

H 4.76599600 1.52353700 -1.98909600

C 1.33929400 0.15129600 0.82400700

C 0.71404100 -0.82667100 1.54342000

H 1.23416400 -1.71736300 1.86433300

H -0.23671600 -0.62596100 2.02196100

N 0.62680400 1.32609300 0.49062700

N -0.59702400 1.20416200 0.29025500

C -0.46098900 3.72712700 0.14499000

H -0.01999700 3.81584100 1.14066100

H 0.34951100 3.71762800 -0.58673200

H -1.07994000 4.60908600 -0.03812600

C 0.82624700 -3.01677100 -0.45312600

H 0.44646600 -3.68083600 0.32445800

H 0.68201600 -3.51804700 -1.42591200

H 1.91159200 -2.88112800 -0.34672300

C 0.07531200 -1.74304900 -0.58041500

H 0.61249300 -1.07434600 -1.27056300

C -3.59632700 -1.26651200 -0.20224100

O -4.70732900 -1.53792400 -0.36614300

Pd -1.73789300 -1.00984300 -0.05892700

C -1.33597600 2.47783700 0.03082400

C -1.91387000 2.34491400 -1.38809500

H -2.53859300 1.45271600 -1.47494100

H -2.52519100 3.22222900 -1.61498400

H -1.11011100 2.28055900 -2.12678200

C -2.47079900 2.51367500 1.06867700

H -3.08669700 3.40139200 0.90295700

H -3.10269300 1.62686400 0.98392500

H -2.06632500 2.55848200 2.08405200

**I-Im6**

C 4.92829700 0.21566800 -0.53918200

C 4.76851100 -0.05172300 0.82225200

C 3.51896600 0.07899900 1.42082500

C 2.40474600 0.47323100 0.66428500

C 2.57624700 0.74796300 -0.70111900

C 3.82895900 0.61748800 -1.29718700

H 5.90344000 0.11572900 -1.00285400

H 5.62126100 -0.35341200 1.42048900

H 3.40835000 -0.11293800 2.48171500

H 1.72566000 1.04277900 -1.30354100

H 3.94400800 0.82571500 -2.35522000

C 1.05761200 0.55769500 1.26791100

C 0.61221800 -0.15564000 2.30983300

H 1.24538400 -0.88036300 2.80555300

H -0.38759900 -0.00507600 2.69856000

N 0.21181600 1.55625500 0.68572100

N -0.82453200 1.14348000 0.14666900

C -1.14929500 3.61098600 -0.28840600

H -0.95132500 3.89529000 0.74686900

H -0.21407700 3.68323500 -0.84832900

H -1.86186400 4.32331600 -0.71152200

C 1.02167700 -2.97879100 -1.24571700

H 1.40265800 -2.93991600 -0.22371900

H 0.82743300 -4.02700300 -1.52142600

H 1.81954500 -2.64426800 -1.92895200

C -0.18670300 -2.13915800 -1.48425300

H -0.50956700 -2.27674800 -2.52777000

C -2.70108400 -1.52651300 0.88425900

O -3.56366900 -1.96227900 1.50635400

Pd -1.21781200 -1.06425100 -0.24464600

C -1.74838200 2.20778700 -0.38501700

C -2.03625400 1.83790800 -1.84655000

H -2.46200200 0.83393000 -1.92010900

H -2.75009800 2.55103400 -2.26624500

H -1.12075500 1.87042400 -2.44310800

C -3.02662900 2.10324800 0.46282000

H -3.74716700 2.84812800 0.11605200

H -3.48009900 1.11454800 0.37435300

H -2.80879600 2.29633000 1.51684200

**II-Im6**

C -5.80458900 -0.42893500 -0.47634700

C -5.03796600 -1.57075600 -0.69750800

C -3.65474000 -1.53911300 -0.51246600

C -3.00561200 -0.35952100 -0.11108200

C -3.79425800 0.78639300 0.10543000

C -5.17242000 0.75085300 -0.07119100

H -6.87980100 -0.45458600 -0.61526600

H -5.51344400 -2.49197400 -1.01704200

H -3.08271500 -2.43942900 -0.70158600

H -3.31156600 1.70268300 0.42121800

H -5.75922900 1.64475900 0.11233600

C -1.53024500 -0.32104000 0.07365300

C -0.81204900 -1.63296700 0.34432000

H -1.51206000 -2.25126400 0.92351300

H -0.62754900 -2.19526100 -0.58128800

N -0.98300700 0.84570700 0.03020300

C -0.04933600 3.49609000 0.25603400

H 0.18665300 3.63904300 -0.80233800

H -1.12844700 3.37541000 0.35481900

H 0.24893700 4.40043100 0.79437900

C 0.99288400 -2.87171800 1.61873900

H 1.14655300 -3.55689900 0.77952400

H 1.93124600 -2.77928900 2.17065800

H 0.25955500 -3.33873600 2.29157500

C 0.46818400 -1.52136900 1.16342700

H 0.32056800 -0.86324000 2.02117500

C 3.08451400 -1.84023900 -0.28929200

O 3.89536100 -2.63539000 -0.37779100

C 0.70601700 2.28297300 0.82866300

C 0.33496300 2.08263700 2.30704500

H 0.90481100 1.25531900 2.73890500

H 0.55721500 2.98670500 2.88198400

H -0.73022600 1.86252200 2.41526400

C 2.21504600 2.53273700 0.71215500

H 2.48397700 3.42002100 1.29142300

H 2.78889600 1.68917000 1.11064300

H 2.51636800 2.69867800 -0.32391800

Pd 1.75699700 -0.47035900 -0.08659800

N 0.36404500 1.04295900 0.03999600

H 0.79611100 1.50997400 -1.61802800

O 2.50249600 0.87811700 -2.14134100

H 2.48366500 0.21203500 -2.84865400

O 1.32576400 1.67147600 -2.45795200

**II-TS2**

C 5.61589100 -0.54678900 0.34127700

C 4.84622700 -1.70506600 0.26141300

C 3.47364300 -1.62719300 0.04176700

C 2.82902100 -0.38336300 -0.09798500

C 3.62397800 0.77927800 -0.01032600

C 4.99234600 0.69613400 0.20141300

H 6.68477000 -0.60866500 0.51056800

H 5.31432500 -2.67687200 0.37342200

H 2.90296500 -2.54607500 -0.00025900

H 3.14725800 1.74452100 -0.11677000

H 5.58042400 1.60577300 0.25747900

C 1.37882200 -0.29718100 -0.31213900

C 0.62624000 -1.54994700 -0.74896500

H 1.25843100 -2.01959300 -1.51865600

H 0.57907500 -2.27233500 0.07352600

N 0.86377200 0.92881200 -0.37685800

C -0.36662000 3.29261400 0.74119200

H -0.84120600 2.84968800 1.61953300

H 0.71845100 3.23864100 0.85246100

H -0.65494200 4.34580800 0.68117700

C -1.35370400 -2.71295600 -1.81802400

H -1.40541000 -3.43540500 -0.99864300

H -2.35534800 -2.58820600 -2.23350800

H -0.72430900 -3.15419400 -2.60280500

C -0.76055700 -1.38568600 -1.37138500

H -0.74172100 -0.68114300 -2.20261200

C -3.13734200 -1.66735200 0.38851700

O -4.03276700 -2.36302300 0.49553900

C -0.81125900 2.55010000 -0.53343300

C -0.14991600 3.16297400 -1.77964600

H -0.46050200 2.62751700 -2.68100400

H -0.44467200 4.21084900 -1.88176400

H 0.93692500 3.11043000 -1.70425200

C -2.33584000 2.61532900 -0.66807600

H -2.64788700 3.65472200 -0.79705900

H -2.67902400 2.04524600 -1.53503800

H -2.82962100 2.22685700 0.22642600

Pd -1.73177400 -0.40311600 0.18027400

N -0.41082700 1.11274500 -0.42254900

H -1.83314300 -0.09403300 3.00231700

O 0.11374300 -0.10628200 1.97243200

H 0.47101300 0.79232600 2.00683700

O -1.61977900 0.53820600 2.30181400

**II-Im7**

C -5.61871800 -0.48541900 -0.66611200

C -4.92567400 -1.64113200 -0.30518600

C -3.57722800 -1.57632200 0.03154600

C -2.88673100 -0.34794300 0.01113000

C -3.60359500 0.81182200 -0.35665600

C -4.94928600 0.74135500 -0.68906300

H -6.67022500 -0.53839200 -0.92532700

H -5.43628100 -2.59738200 -0.28615500

H -3.06447200 -2.48950300 0.30281500

H -3.09312100 1.76558000 -0.37127000

H -5.48147700 1.64491300 -0.96502800

C -1.45696600 -0.27461200 0.34092500

C -0.73453400 -1.50855600 0.82458800

H -1.32811100 -1.90078800 1.66446700

H -0.81319700 -2.27211100 0.03914100

N -0.90346000 0.91775800 0.22624300

C 0.62875800 3.26337100 -0.88022500

H 1.26162400 2.77805300 -1.62536100

H -0.41030100 3.24333500 -1.21883200

H 0.93830500 4.30616000 -0.76463000

C 1.25772500 -2.73148600 1.73413100

H 1.20718200 -3.48367400 0.94032400

H 2.29695500 -2.65561200 2.06391400

H 0.66989300 -3.11134600 2.58189400

C 0.71572200 -1.38060500 1.27186800

H 0.78764400 -0.66885700 2.10008700

C 2.88457900 -1.86333400 -0.63889000

O 3.59971300 -2.71272000 -0.89062300

C 0.76612000 2.54002000 0.47252500

C -0.14291400 3.17350300 1.53516400

H -0.10606200 2.60295500 2.46779700

H 0.20693900 4.18838400 1.73988700

H -1.18005300 3.22820500 1.20238000

C 2.22270000 2.56336800 0.94774500

H 2.52025600 3.59771700 1.13815800

H 2.33924100 1.99854100 1.87717800

H 2.88417600 2.14428300 0.18805800

Pd 1.74292400 -0.40775300 -0.28418600

N 0.37909600 1.10957900 0.23928600

H 3.22511300 0.31874800 -2.32891800

O 2.55806900 0.80795700 -1.83533500

**II-Im8**

C -5.81509300 -0.53285900 -0.51189600

C -5.08011100 -1.71343200 -0.40379700

C -3.71502000 -1.67114000 -0.13332500

C -3.05263900 -0.43963800 0.02650200

C -3.80951300 0.74557600 -0.08261300

C -5.17144100 0.69686600 -0.34806000

H -6.87921200 -0.56901600 -0.71660100

H -5.57026200 -2.67222300 -0.53093000

H -3.16725600 -2.60212300 -0.06210900

H -3.31927600 1.70076100 0.05588000

H -5.73650900 1.61947800 -0.42148200

C -1.60317800 -0.38675900 0.26838300

C -0.82097900 -1.62866900 0.57594900

H -1.39900400 -2.25757800 1.26490000

H -0.75303600 -2.20846900 -0.36272100

N -1.04124000 0.78190300 0.15677600

C 0.23393600 3.26182400 -1.09003600

H 0.92943700 2.86579800 -1.83239800

H -0.79109200 3.10756900 -1.43692600

H 0.40585000 4.33590200 -0.97347500

C 1.23226000 -2.71192100 1.56511600

H 1.22616900 -3.44897000 0.75589000

H 2.26251800 -2.53351600 1.87740500

H 0.69771600 -3.15546200 2.41649700

C 0.55589900 -1.41798000 1.15569700

H 0.53866300 -0.71626300 1.99178800

C 2.77096600 -1.87713600 -0.92879700

O 3.56045500 -2.63876100 -1.20566900

C 0.46017900 2.56100500 0.26256900

C -0.50225400 3.09521300 1.33183600

H -0.42115400 2.51197000 2.25344400

H -0.23258000 4.12977900 1.55805500

H -1.54091100 3.07855700 1.00015200

C 1.90297500 2.76595100 0.72722800

H 2.06278500 3.83525300 0.88802900

H 2.08619200 2.24354100 1.66775100

H 2.60835300 2.41440000 -0.02563400

Pd 1.65971900 -0.32585800 -0.24979400

N 0.20269000 1.09149300 -0.01788200

H 3.39688800 0.44577500 -2.11169000

O 2.68351800 0.92326400 -1.67275300

O 3.00655900 -0.18904200 1.34697200

H 3.59831700 0.50545100 1.02552100

**II-TS3**

C -5.82716300 -0.57689600 -0.46658800

C -5.07007600 -1.74325800 -0.36353500

C -3.69768200 -1.67470800 -0.13419500

C -3.05186900 -0.43158300 -0.01253100

C -3.82915200 0.73885100 -0.11877500

C -5.19815500 0.66482700 -0.34109600

H -6.89612700 -0.63272100 -0.63919800

H -5.54735500 -2.71186600 -0.46270200

H -3.13182400 -2.59537600 -0.06721300

H -3.35020300 1.70396100 -0.01251700

H -5.77950900 1.57757300 -0.41159200

C -1.59426900 -0.35450000 0.18913500

C -0.79301600 -1.56735900 0.56933900

H -1.37774100 -2.19431600 1.25355900

H -0.65476800 -2.17756300 -0.34348500

N -1.03552300 0.80159000 0.01168800

C -0.60151300 3.48122800 -0.26037700

H -0.67288900 3.40723200 -1.34882000

H -1.57727000 3.25639200 0.17043700

H -0.33575600 4.50880200 -0.00087200

C 1.22928300 -2.49170700 1.78084800

H 1.26012200 -3.32218100 1.06871300

H 2.24580200 -2.25563800 2.10106800

H 0.67422000 -2.83744300 2.66377200

C 0.54586800 -1.27067600 1.20117900

H 0.48026700 -0.47850000 1.94776300

C 2.82737900 -1.96916700 -0.73900000

O 3.66830900 -2.71505300 -0.86343700

C 0.49082400 2.53993100 0.27923400

C 0.48881700 2.52354700 1.81566700

H 1.26212000 1.84620500 2.18283400

H 0.69439400 3.53028100 2.19004800

H -0.48366000 2.20655100 2.20223300

C 1.84896600 2.98907400 -0.25749000

H 1.99686900 4.03919800 0.00792700

H 2.66161800 2.41066200 0.17637800

H 1.90062200 2.87805700 -1.34013500

Pd 1.64801900 -0.34824900 -0.31726900

N 0.19341400 1.13579900 -0.21384000

H 3.19411600 0.00327000 -2.45016500

O 2.62153000 0.61660400 -1.97498800

O 2.87176300 0.17047500 1.27117500

H 3.62054900 0.57330000 0.80849300

**II-Im9**

C 5.58350400 0.11982600 -0.05795800

C 5.01477400 -1.12003300 0.22636900

C 3.62875200 -1.27641200 0.23359200

C 2.78100400 -0.18926100 -0.03855600

C 3.37000400 1.05749200 -0.32514900

C 4.75115700 1.20831800 -0.33492200

H 6.66117700 0.23914500 -0.06742700

H 5.64876700 -1.97146500 0.44862600

H 3.21367300 -2.24719300 0.47508100

H 2.73257500 1.90282000 -0.55303700

H 5.18264400 2.17610300 -0.56689900

C 1.30903500 -0.34230000 -0.00662900

C 0.69861400 -1.72845700 -0.11107100

H 1.37037200 -2.33465500 -0.73150700

H 0.66345900 -2.19082500 0.88171100

N 0.62493900 0.73562400 0.15994900

C -0.25073500 3.34242200 0.70399700

H -0.28157000 3.20268500 1.78760900

H 0.78628300 3.28109700 0.37324600

H -0.63589000 4.33855300 0.47085500

C -1.28722600 -3.18784200 -0.73161800

H -1.34376500 -3.58886900 0.28363100

H -2.27997500 -3.19066900 -1.18600600

H -0.65979100 -3.86953100 -1.32366100

C -0.65588100 -1.81612300 -0.76393300

H -0.67286300 -1.37037400 -1.75868400

C -1.12579500 2.29164800 -0.00858300

C -1.01721900 2.43892100 -1.52866600

H -1.59539600 1.66045500 -2.02983200

H -1.39564400 3.41820800 -1.83494100

H 0.02515100 2.35948000 -1.84879500

C -2.56753300 2.45460300 0.47976100

H -2.90135000 3.47604000 0.28312000

H -3.26336100 1.78582500 -0.03258200

H -2.63613700 2.27282700 1.55566300

Pd -1.99251400 -0.60849900 0.22136900

N -0.61806800 0.93926800 0.45695500

O -1.44710800 -1.20631300 2.05808400

H -0.85402900 -0.54021700 2.42755800

O -2.80965300 -0.34270000 -1.60917800

H -3.49741600 0.33254700 -1.56607000

**II-TS4**

C 4.96657000 0.33093100 -0.55726900

C 4.47862700 -0.85844100 -0.01156300

C 3.20139200 -0.90771200 0.53405900

C 2.38405900 0.23651700 0.54001700

C 2.88221900 1.42677400 -0.01838700

C 4.16340500 1.47143100 -0.55826600

H 5.96434100 0.36645900 -0.98009400

H 5.09359200 -1.75136600 -0.01682900

H 2.83257100 -1.84455500 0.93204000

H 2.26987100 2.31978200 -0.01787800

H 4.53504600 2.39834000 -0.98023700

C 1.02055600 0.17371600 1.07637700

C 0.51168000 -0.83559300 1.85751300

H 1.17768400 -1.55316300 2.31694000

H -0.45690100 -0.70136600 2.31954600

N 0.22971500 1.30637300 0.85577200

N -0.80073000 1.27264700 0.18988400

C -0.93773000 3.73611100 0.72749800

H -0.64342300 3.59784200 1.76996000

H -0.04311700 3.96880500 0.14376200

H -1.61685400 4.59041100 0.67213500

C -0.85829700 -3.27913200 1.30152500

H -1.72962100 -2.90193300 1.83490000

H -1.18277400 -4.15404600 0.71791200

H -0.09712800 -3.62267800 2.00960300

C -0.31934500 -2.30589800 0.31323100

H 0.69593300 -2.47467100 -0.04274400

C -1.64586500 2.49996500 0.17687400

C -2.05060700 2.68699500 -1.29136500

H -2.54479400 1.79072600 -1.67530900

H -2.74314200 3.52784200 -1.37591700

H -1.17513300 2.89317500 -1.91305200

C -2.86950600 2.12000300 1.03224600

H -3.61454500 2.91664600 0.96196300

H -3.30998800 1.18038900 0.69073700

H -2.57987400 2.00402200 2.08051000

O -3.06253000 -1.26205400 0.33780600

H -3.40755400 -2.15613300 0.23798500

Pd -1.32666000 -1.08641600 -0.70907100

O 0.24482900 -0.72494500 -1.93968900

H 0.57796500 -1.55052200 -2.30815200

**II-Im10**

C 5.21080200 -0.82860600 -0.54828700

C 4.36382000 -1.85726400 -0.13169400

C 3.11059000 -1.56369400 0.39837200

C 2.68765100 -0.23225400 0.53146900

C 3.53901700 0.79621700 0.09675000

C 4.79229000 0.49701400 -0.43359000

H 6.18439300 -1.05946600 -0.96620800

H 4.67402200 -2.89149200 -0.23245400

H 2.44728900 -2.36953900 0.69006400

H 3.22488600 1.82853900 0.18688700

H 5.44226700 1.30247300 -0.75746900

C 1.35330000 0.06703900 1.09682900

C 0.72221700 -0.64008500 2.04525700

H 1.19610300 -1.50023600 2.50117100

H -0.25189000 -0.34093100 2.41254600

N 0.78747600 1.28989000 0.62673900

N -0.36244000 1.25193100 0.17688600

C 0.10288500 3.69024000 -0.27525300

H 0.61302000 3.85167800 0.67539200

H 0.85220400 3.44873000 -1.03363200

H -0.39137400 4.62142900 -0.56287200

C -3.13529900 -3.20763000 0.13231100

H -3.42918700 -2.84087500 1.11407300

H -4.01218600 -3.50842700 -0.46199100

H -2.55334500 -4.13909600 0.24495300

C -2.31470200 -2.29920400 -0.66062800

H -2.05191500 -2.67786500 -1.65419500

C -0.95704500 2.59301800 -0.17637700

C -1.68428100 2.42385600 -1.51572700

H -2.45893000 1.65560400 -1.45391900

H -2.15968600 3.36986300 -1.78545100

H -0.98651900 2.14623800 -2.30945700

C -1.95753700 2.89023500 0.95424600

H -2.50486500 3.80521400 0.71296900

H -2.65857700 2.06302500 1.07887600

H -1.42838100 3.03903100 1.89985800

O -2.69204100 -0.29061500 1.39328200

H -3.55340800 -0.70828500 1.28869700

Pd -1.52625800 -0.65423700 -0.23077500

O -0.35498200 -1.00247800 -1.87632600

H 0.41261600 -0.42201400 -1.81526500

**Ⅲ-Im3**

C 5.93340900 -1.12476400 -0.70830100

C 5.57270600 -1.14280900 0.63840400

C 4.30066900 -0.72805200 1.02997900

C 3.37489400 -0.27587100 0.07848200

C 3.74902500 -0.25976400 -1.27556000

C 5.01556200 -0.68359400 -1.66426300

H 6.92002200 -1.45634500 -1.01264100

H 6.28071100 -1.48060200 1.38701000

H 4.04022000 -0.73932200 2.08170000

H 3.03359800 0.07172100 -2.01802100

H 5.28519600 -0.67805200 -2.71465800

C 2.02285800 0.18263300 0.48151600

C 1.28578500 -0.44934900 1.62539500

H 1.94917000 -1.11429000 2.18357000

H 0.95202600 0.31981700 2.33277000

H 0.07292700 1.57636500 1.12653200

N 1.48375300 1.09465300 -0.24434500

N 0.15343600 1.47111800 0.10901800

H -0.01060100 -2.41528600 2.99749000

C 0.07195800 -1.28243100 1.16084700

H 0.42297400 -2.10951600 0.53625700

C -0.68559400 -1.81277100 2.37189000

H -1.52192600 -2.44714500 2.08057100

H -1.06530800 -1.00071300 2.99678100

C -0.14780000 2.84330100 -0.48165300

C -0.04729100 2.78723400 -2.00582100

H 0.96332400 2.52789100 -2.32245300

H -0.73561800 2.05644200 -2.43294800

H 0.65223400 4.84356800 -0.27377400

H 0.85012400 3.84870800 1.17835400

H 1.89406500 3.57998100 -0.23148400

Pd -1.14907100 -0.27813100 -0.23403500

O -2.66360100 1.01791000 2.51336700

H -2.39593100 0.65923100 1.64136400

O -1.39460500 1.53605100 2.99791200

H -1.62583500 2.46424000 3.16807600

Br -2.38310000 -2.48994700 -0.64828900

H -0.29817800 3.76795400 -2.41677400

C -1.54627200 3.26680700 -0.02652600

H -1.76521500 4.25792100 -0.42917200

H -2.32609300 2.58839100 -0.37107900

H -1.60595900 3.33007600 1.06188400

C 0.88198900 3.83714400 0.08409200

C -2.47766900 0.44641100 -1.59139600

O -3.31502600 0.68428900 -2.31738300

**Ⅲ-Im4**

C 5.90254100 -1.07326500 -0.56039000

C 5.40911900 -1.33388900 0.71281400

C 4.10488100 -0.98337600 1.04326400

C 3.27788100 -0.35366100 0.10762300

C 3.78674800 -0.09312900 -1.17154700

C 5.08476000 -0.45320300 -1.50231800

H 6.91458200 -1.35427700 -0.81971200

H 6.03838400 -1.81076100 1.45239900

H 3.74253900 -1.18423700 2.04154400

H 3.15122900 0.37940000 -1.90643300

H 5.45805800 -0.25692900 -2.49870200

C 1.89127900 0.04162800 0.45194200

C 1.06169100 -0.78813100 1.38978600

H 1.67877600 -1.56194300 1.84695300

H 0.66957800 -0.17988200 2.20789200

H -0.03501000 1.53773200 1.15677200

N 1.42530100 1.07410900 -0.14305200

N 0.06912300 1.40685000 0.14701800

H -0.15884300 -3.11308500 2.09582000

C -0.09396100 -1.50948000 0.68161700

H 0.28153000 -2.08406600 -0.16805100

C -0.85635100 -2.39532200 1.64685000

H -1.64194100 -2.95437900 1.14696100

H -1.30071500 -1.82091600 2.45980300

C -0.29315500 2.72640600 -0.51100000

C -0.22760400 2.55651600 -2.02715100

H 0.78092400 2.30698600 -2.35148800

H -0.90223400 1.76634600 -2.36183200

H 0.38281700 4.77281200 -0.45649000

H 0.65888000 3.89695000 1.05271000

H 1.69697700 3.59552400 -0.34845200

Pd -1.28243900 -0.18326600 -0.31140500

O -2.49717700 0.52533000 2.60591500

H -2.26048600 0.29800500 1.67487200

O -1.33068700 1.28319800 3.01841800

H -1.71232300 2.16712400 3.13900100

Br -3.01727300 -1.72194300 -1.10219700

H -0.52484900 3.48749200 -2.50969600

C -1.71461900 3.08344000 -0.07157100

H -1.99697200 4.03686700 -0.51678900

H -2.44214700 2.33660800 -0.39349300

H -1.78336800 3.18791700 1.01157000

C 0.67734200 3.81113200 -0.03593100

**Ⅲ-TS2**

C 5.90169600 -1.09903900 -0.67715000

C 5.36357500 -1.55939200 0.51851700

C 4.06008200 -1.22984600 0.87244900

C 3.27669300 -0.42376500 0.03961600

C 3.83235000 0.03779700 -1.16230800

C 5.12906300 -0.29984200 -1.51729100

H 6.91353500 -1.36187400 -0.95542000

H 5.95733000 -2.17540100 1.18044400

H 3.66759700 -1.58799400 1.81333200

H 3.23390100 0.65213900 -1.81894100

H 5.53783200 0.05545200 -2.45386500

C 1.89200600 -0.04877200 0.41034200

C 1.04681900 -0.95946500 1.25457700

H 1.65458100 -1.77657000 1.64325000

H 0.63331900 -0.43564900 2.11765900

H -0.02905000 1.49022300 1.25609300

N 1.43996100 1.04323000 -0.08206200

N 0.11175900 1.39333500 0.23128800

H -0.02870100 -3.44000300 1.55723300

C -0.07686800 -1.62580900 0.47214000

H 0.24558300 -1.96749100 -0.51431000

C -0.77857100 -2.69437500 1.26827100

H -1.55124300 -3.19680700 0.69660100

H -1.21218200 -2.29184600 2.18269100

C -0.23887900 2.72274300 -0.41618300

C -0.20043600 2.55483700 -1.93336800

H 0.80122900 2.30811200 -2.28161700

H -0.88355700 1.76357900 -2.25204700

H -0.51218000 3.48400700 -2.41013600

C -1.64164600 3.12072900 0.04895800

H -1.84953800 4.13403800 -0.29330700

H -2.40992200 2.46945800 -0.37004800

H -1.72045400 3.08704300 1.13411400

C 0.76828400 3.77994400 0.05027700

H 0.50119800 4.74456200 -0.38192600

H 0.75024100 3.87631100 1.13689300

H 1.78034600 3.53008400 -0.26090400

Pd -1.37402500 -0.15621900 -0.19108900

O -1.84672500 0.40971000 1.68502900

H -2.69081700 0.05182800 2.01681500

O -0.92115500 1.69763200 3.11018100

H -0.68180100 0.92737500 3.64033100

Br -3.24646000 -1.56353400 -0.92491900

**Ⅲ-Im5**

C 5.88280100 -0.25799700 -0.87110600

C 5.32933900 -1.37751400 -0.26147800

C 3.96201000 -1.44176500 -0.01787700

C 3.12095300 -0.37990000 -0.37607600

C 3.69340300 0.74521100 -0.99102200

C 5.05596600 0.80345000 -1.23519400

H 6.94596000 -0.21226200 -1.06548300

H 5.96155700 -2.20575900 0.02974900

H 3.56008400 -2.31657300 0.47237600

H 3.05676500 1.56517800 -1.29061000

H 5.47560300 1.67534600 -1.71936800

C 1.67307900 -0.43032900 -0.09351900

C 1.01180800 -1.76317300 0.18763000

H 1.56958600 -2.52602000 -0.36363900

H 1.10663200 -1.99450800 1.25149200

H 0.74622600 1.46399600 2.28604000

N 1.05889300 0.69423100 -0.04812600

N -0.09400900 1.04466600 0.41693500

H -0.53454400 -4.03086500 -0.31610100

C -0.41725000 -1.91535200 -0.24942100

H -0.53571600 -1.71216900 -1.31017100

C -1.07208500 -3.20595800 0.16736000

H -2.10869000 -3.26346500 -0.16087100

H -1.01877100 -3.36064300 1.24456200

C -0.59724600 2.35736800 -0.16467400

C -0.63730900 2.33204300 -1.68955000

H 0.35736200 2.15386300 -2.09902500

H -1.30937800 1.55795200 -2.05261200

H -0.99131700 3.29499100 -2.05915100

C -1.96284100 2.65945500 0.44732400

H -2.24526900 3.68222900 0.19811700

H -2.74312500 2.00612300 0.06091400

H -1.92843400 2.57513000 1.53454400

C 0.39943800 3.42960500 0.31569400

H 0.07268400 4.39838200 -0.06233700

H 0.42909400 3.48640100 1.40403000

H 1.40230100 3.22945200 -0.05525300

Pd -1.57132300 -0.44112200 0.61486600

O -0.88949100 -0.83569400 2.42204900

H -0.20358800 -0.22218700 2.72023200

O 1.06407700 1.26578300 3.18119000

H 2.00357400 1.07168900 3.07527800

Br -3.07402900 -0.42767100 -1.38350400

**Ⅲ-Im6**

C 6.00633200 -0.08150200 -0.49675700

C 5.41443000 -1.30107000 -0.17357000

C 4.03085500 -1.39845400 -0.03324700

C 3.21191000 -0.27034200 -0.20980700

C 3.82263700 0.95560800 -0.53724200

C 5.20156500 1.04672600 -0.67877600

H 7.08221200 -0.00893400 -0.60999500

H 6.02899200 -2.18206600 -0.02508100

H 3.59850100 -2.35384500 0.23598500

H 3.20420600 1.83084800 -0.69395000

H 5.65181800 1.99830500 -0.93940200

C 1.74732500 -0.36120800 -0.03292200

C 1.07559200 -1.72127200 -0.04323000

H 1.61691700 -2.34424400 -0.76651100

H 1.20080300 -2.17588200 0.94566500

N 1.12203700 0.74300600 0.17660000

N -0.02773600 1.05239000 0.64370400

H -0.46736800 -3.86530100 -0.87203100

C -0.37297800 -1.78134300 -0.45260600

H -0.52967500 -1.38226400 -1.45430700

C -1.01356100 -3.13551800 -0.25691200

H -2.05403600 -3.14872300 -0.58371200

H -0.94872500 -3.47048400 0.78172200

C -0.52721100 2.41206500 0.18133400

C -0.60059400 2.50428000 -1.34313100

H 0.38014600 2.32254400 -1.79046000

H -1.31120300 1.78069700 -1.74444200

H -0.92832100 3.50628500 -1.63258400

C -1.87964400 2.67555800 0.84530500

H -2.16257300 3.71717000 0.67604900

H -2.67219400 2.05158900 0.43018100

H -1.81980400 2.50930300 1.92443000

C 0.49983100 3.42156500 0.73766100

H 0.14648400 4.43277100 0.52159800

H 0.60444300 3.31337000 1.82006300

H 1.47728400 3.28244300 0.27453200

Pd -1.50567400 -0.49631500 0.70372500

O -0.55260300 -1.05740500 2.37735900

H 0.03069100 -0.33163900 2.63570200

Br -3.19445100 -0.24430100 -1.18006800

**Ⅲ-TS3**

C -4.94169500 0.51437000 0.12758700

C -4.45457200 -0.58750100 -0.57887300

C -3.15902000 -0.58128300 -1.08117800

C -2.32288800 0.53184700 -0.88415700

C -2.82065500 1.63271200 -0.16596300

C -4.11967600 1.62245200 0.33181900

H -5.95328000 0.50657300 0.51768500

H -5.08441600 -1.45649100 -0.73241500

H -2.79235700 -1.45343500 -1.60783000

H -2.19314900 2.50064100 -0.00761600

H -4.49008400 2.48130200 0.88006000

C -0.94075300 0.52204100 -1.37922600

C -0.42241400 -0.36131700 -2.28691000

H -1.07488400 -1.00779300 -2.85747700

H 0.58092100 -0.21223000 -2.66243500

N -0.13864800 1.59362600 -0.94949900

N 0.88480600 1.40765600 -0.29650600

C 1.05932900 3.92565700 -0.31415400

H 0.77832900 4.01032100 -1.36586400

H 0.15928700 4.04496200 0.29487600

H 1.74740900 4.74005700 -0.07481200

C 0.73817800 -3.02094900 -1.98784400

H 1.59939700 -2.64120700 -2.53417000

H 1.03984800 -3.96396500 -1.50464400

H -0.08311100 -3.25458800 -2.67271900

C 0.30177300 -2.13378100 -0.88574400

H -0.71673000 -2.25922400 -0.52329600

C 1.74558400 2.59272300 -0.02252000

C 2.13218400 2.46927200 1.45756000

H 2.62100000 1.51154000 1.65479200

H 2.82477900 3.27199500 1.72164000

H 1.25012500 2.54290700 2.09860200

C 2.97730200 2.38174300 -0.92266100

H 3.73085100 3.13460400 -0.67806900

H 3.40093700 1.38520700 -0.77944300

H 2.70309800 2.49015100 -1.97574700

O 2.93537700 -1.06899600 -1.11193100

H 3.32450400 -1.95171200 -1.11007700

Pd 1.38849600 -1.05609500 0.20279100

Br -0.30244300 -1.00983400 2.10747600

**Ⅲ-Im7 (IntC+H)**

C -5.23673600 -0.71113200 0.40220800

C -5.00738500 -0.95100100 -0.95435900

C -3.78263300 -0.62077700 -1.52737800

C -2.76279800 -0.05277700 -0.74871300

C -3.00350900 0.18918100 0.61188100

C -4.23185600 -0.13913800 1.18182900

H -6.19299800 -0.96541500 0.84598100

H -5.78773100 -1.38601100 -1.56911900

H -3.62175100 -0.78823900 -2.58603900

H -2.21977400 0.60701300 1.23156800

H -4.40023100 0.04650400 2.23691900

C -1.44253100 0.27460000 -1.33196700

C -0.86321900 -0.34127300 -2.37184500

H -1.35095900 -1.17763400 -2.85719300

H 0.09006500 -0.00843000 -2.76295300

N -0.84588500 1.44448900 -0.76985300

N 0.30969400 1.37389400 -0.34025600

C 0.47586500 3.83640100 -0.75880200

H 0.86751200 3.64701500 -1.76209900

H -0.60443500 3.96914800 -0.82354800

H 0.91702000 4.76382700 -0.38585600

C 2.77629400 -3.01777000 -1.23403400

H 2.53228000 -2.63158500 -2.22229100

H 3.80999200 -3.38878000 -1.17863900

H 2.15617600 -3.91027800 -1.02613000

C 2.50925800 -2.10364800 -0.13259100

H 2.86375900 -2.45045000 0.84232900

C 0.84895900 2.69238900 0.19006500

C 0.21706200 2.88477200 1.57755600

H 0.43758900 2.03397500 2.22582900

H 0.62475900 3.79030600 2.03392000

H -0.86705000 2.99116400 1.49420600

C 2.36960400 2.56860000 0.30268600

H 2.77618300 3.51357700 0.67116300

H 2.65643500 1.78282000 1.00685100

H 2.81699900 2.34480000 -0.66752400

O 2.34836000 0.05715200 -2.01178300

H 3.25378800 -0.26790100 -2.07868500

Pd 1.54906800 -0.49244500 -0.22338000

Br 0.45009900 -1.05998300 2.00664600

**Int-C**

C -4.11116700 -1.06616100 0.00939300

C -4.11103800 0.31508200 0.21577900

C -2.91492600 1.02646800 0.20808200

C -1.69322800 0.37117000 -0.01599200

C -1.70458400 -1.01874400 -0.21330900

C -2.90351500 -1.72960200 -0.20342900

H -5.04380200 -1.61928600 0.02019000

H -5.04435800 0.83777000 0.39452200

H -2.92903000 2.09416500 0.39302300

H -0.77350900 -1.54584900 -0.38468300

H -2.89237300 -2.80217700 -0.36337300

C -0.41305400 1.11512600 -0.06710200

C -0.27278300 2.41697400 -0.35117200

H -1.12365300 3.04141300 -0.59266400

H 0.70436400 2.88388500 -0.32133400

N 0.72864400 0.33966300 0.32933700

N 1.70963100 0.41749700 -0.41957500

C 2.76772100 -1.03392700 1.37506600

H 2.52555700 -0.33214600 2.17679200

H 1.97745700 -1.78705700 1.33429700

H 3.70734300 -1.53315500 1.62610300

C 2.92593900 -0.31646200 0.03432600

C 3.23326400 -1.31633500 -1.09260300

H 3.32947800 -0.80513300 -2.05400400

H 4.17204800 -1.83439700 -0.87983200

H 2.43783900 -2.06269600 -1.17467600

C 4.02648800 0.75514300 0.11085100

H 4.98270700 0.28275300 0.35068000

H 4.12819900 1.28044100 -0.84224900

H 3.79911100 1.48895400 0.88952300

**Ⅲ-Im8 (Int-H)**

C -1.29844700 2.38021900 -0.30423700

H -1.34626600 2.15725000 -1.36832600

H -2.30048400 2.67148000 0.06052000

H -0.65696400 3.25140800 -0.10344500

C -0.87584800 1.28019900 0.54377300

H -0.72574100 1.47614100 1.60938500

O -2.40360800 -1.20443800 -0.19941300

H -2.80558700 -1.33566700 0.66946400

Pd -0.58436200 -0.43462600 0.00621000

Br 1.91400900 -0.01585200 -0.02843400

**Ⅲ-TS4**

C -1.69920100 2.14925500 -0.27291900

H -1.52339500 2.03574000 -1.34064700

H -2.73970300 2.42747300 -0.05425900

H -1.09121900 2.98860300 0.11517400

C -1.32038400 1.01496400 0.54439400

H -1.58385500 1.03760300 1.60524500

O -2.44846600 -1.05724900 -0.13343500

H -2.67352700 -1.39813600 0.74299400

Pd -0.46085200 -0.49126400 -0.04108700

Br 1.95760400 0.14227200 0.00743300

**Ⅲ-Im9**

C -1.68633800 1.89348500 -0.41732900

H -1.87858500 1.60729600 -1.45430100

H -2.50930200 2.53630800 -0.07937700

H -0.75670400 2.46111100 -0.36803500

C -1.60402400 0.69328300 0.47874400

H -1.38057600 0.89805000 1.52860900

O -2.68335300 -0.17332000 0.30412800

H -2.86011100 -0.65970800 1.12567600

Pd -0.35315400 -0.69909000 -0.15199200

Br 1.90969600 0.31945700 0.09821600

**Ⅲ-TS5**

H 0.73104500 -1.45677900 -0.63840900

C 2.43045900 0.19025400 0.39359100

Pd 0.29656600 -0.16705600 0.05401800

Br -2.17882400 0.15343300 -0.03403300

C 2.31421000 -1.07403200 -0.19086100

H 2.39197500 -1.93761700 0.45967200

H 2.65761400 -1.20384100 -1.21234600

H 2.46618100 0.31588600 1.47387500

O 2.87923300 1.23201600 -0.34700800

H 2.86811700 2.04327800 0.18322200

**Ⅲ-Im10**

H -0.35425800 0.36685900 1.31576500

C -2.52116400 0.16000000 -0.40511000

Pd -0.27775500 -0.23386400 -0.04813100

Br 2.18153000 0.18941000 0.01671800

C -2.28379800 -1.03179000 0.24731700

H -2.22424400 -1.94340300 -0.33810700

H -2.53107200 -1.12886900 1.29890200

H -2.52903000 0.22809300 -1.49103000

O -3.01375700 1.22821000 0.26221500

H -2.99840200 2.01075800 -0.30758100

**Ⅲ-Im11**

C 1.22645300 -0.17924800 -0.00002900

H 1.32393100 -1.25970800 -0.00003100

H 2.12565600 0.42311400 -0.00006500

C 0.03483000 0.41480400 0.00000600

H -0.07675100 1.49648500 -0.00000100

O -1.13206000 -0.29762300 0.00002000

H -1.88404800 0.30776200 0.00007100

**PdBr2**

Pd 0.00000000 0.99758200 0.00000000

Br 1.77183100 -0.65568900 0.00000000

Br -1.77183100 -0.65541900 0.00000000

**Im-a**

Pd -0.11306700 0.20521000 -0.17032400

Br -0.39550300 -2.26542500 0.01559700

Br -2.53089500 0.68130300 0.16737200

H 2.09654500 -1.15650100 -0.33206600

O 1.95451500 -0.21779600 -0.54203100

C 3.06085900 0.58245500 0.03027800

H 2.91284500 1.56713500 -0.41689200

C 4.36511500 -0.02471600 -0.45310600

H 4.39207900 -0.07443800 -1.54368200

H 5.20143600 0.59043500 -0.11142300

H 4.49714300 -1.03271600 -0.04589800

C 2.93597000 0.65154000 1.54102600

H 3.02053700 -0.34673400 1.98110600

H 3.73568500 1.27656300 1.94697800

H 1.97745600 1.08608300 1.83726500

C 0.08810800 2.11266400 -0.27788800

O 0.18185700 3.23818800 -0.32375200

**TS-a**

Pd 0.27025200 0.36737900 0.03481800

Br -0.43305500 -2.06302300 0.06143600

Br 2.81158000 -0.06083300 -0.05141500

H -2.73091700 -1.32749700 0.47652600

O -3.12699600 -0.49484200 0.82710600

C -3.00327200 0.48306300 -0.02029000

H -1.27395200 0.72861000 0.07350800

C -3.37944800 1.81814500 0.50569500

H -3.11952200 1.91713700 1.55875200

H -2.92883200 2.61191400 -0.08806600

H -4.47128000 1.89622200 0.40361500

C -3.01738600 0.20359000 -1.48133200

H -4.07944500 0.11341200 -1.75283800

H -2.58522400 1.02869100 -2.04383600

H -2.51765100 -0.73305900 -1.72642800

C 0.57844900 2.22537100 0.03499200

O 0.74659900 3.34722500 0.03689800

**Im-b**

Pd -0.54908400 0.43678200 -0.41478000

Br 0.63815600 -1.82485000 -0.38998500

Br -2.78603500 -0.37295200 0.70064900

H 2.46694600 -0.82575800 -0.40179900

O 3.34617700 -0.31350300 -0.49035300

C 3.61983100 0.45838100 0.47915700

H 0.73857300 0.97939700 -1.07764200

C 4.87693600 1.21373200 0.39323600

H 5.34624500 1.12445800 -0.58450900

H 4.68810200 2.25898800 0.65731000

H 5.54496200 0.81974100 1.17115300

C 2.71011200 0.61372800 1.62862700

H 3.24788300 0.98345800 2.50057700

H 1.97131700 1.37484500 1.33580100

H 2.16420200 -0.30288400 1.85193900

C -1.18909200 2.19242900 -0.54700500

O -1.57634500 3.25715800 -0.63093300

**HPd(CO)Br**

Pd -0.58255800 -0.56962200 0.00000200

Br 1.78453800 0.34646700 -0.00000100

H -1.88267200 -1.39044100 -0.00005100

C -1.93996700 0.66059400 0.00000000

O -2.76733600 1.43789200 -0.00000100

**Im-a’**

Pd 0.11247200 -0.44304100 -0.23368300

Br 0.35698300 1.99180400 0.02042600

Br 2.47440500 -0.93025700 0.15296600

H -2.16674400 0.78381800 -0.70286200

O -1.94302400 -0.16110700 -0.66862400

C -2.95222600 -0.86663500 0.15230200

H -2.60536100 -1.90156000 0.13887900

C -4.29044200 -0.75208300 -0.55583600

H -4.22664900 -1.14020400 -1.57453600

H -5.04181600 -1.32811600 -0.00937500

H -4.62068500 0.29103800 -0.59199500

C -2.95419100 -0.31992400 1.56851000

H -3.26026700 0.73089700 1.57780800

H -3.66132100 -0.88799600 2.17856000

H -1.96409300 -0.40145800 2.02335100

**TS-a’**

Pd 0.21445600 -0.03408200 -0.18932100

Br -1.74162500 -1.65620800 0.06190800

Br -1.03860800 2.09087800 0.05574900

H 1.64623800 -2.01725900 0.74893200

O 1.84649800 -1.56520300 -0.09519900

C 2.38265200 -0.31649400 0.10122000

H 1.22803000 1.07356200 -0.50969700

C 3.29305700 0.07207500 -1.02535400

H 2.86589800 -0.18886800 -1.99348700

H 3.51451100 1.13781800 -0.98905300

H 4.22652200 -0.48756600 -0.88806900

C 2.75450100 0.06132100 1.50009700

H 3.70910100 -0.42831600 1.73185500

H 2.89389800 1.13953500 1.56754600

H 2.00574400 -0.25436900 2.22859600

**Im-b’**

Pd -0.15428600 0.13376700 -0.61175400

Br 1.87475300 -1.41358000 0.16520600

Br -2.20137100 -0.85384300 0.29425000

H 2.19244500 1.16321100 -0.13203800

O 1.53158000 1.77551400 -0.52858900

C 0.49495900 2.00131200 0.31012400

H -1.23327000 0.96038600 -1.27721700

C -0.35405800 3.15691800 -0.10804000

H -0.41706500 3.24764700 -1.19153700

H -1.34895700 3.07896600 0.32793800

H 0.12464700 4.06008500 0.29385100

C 0.66492600 1.73752200 1.77204800

H 1.18158500 2.61226000 2.19100300

H -0.31000900 1.66093700 2.25188600

H 1.25181100 0.84440100 1.97973100

**TS-b’**

Pd -0.54727600 -0.45769500 -0.01810000

Br 1.17938800 2.21788900 0.01655300

Br -2.97345900 -0.19782800 0.00860200

H 1.79543500 0.53744300 -0.03523200

O 1.66478900 -0.60316100 -0.05088700

C 2.62971500 -1.42226400 -0.00501700

H -0.86801900 -1.92062500 -0.04166000

C 2.31992800 -2.85879600 0.06963300

H 1.31042800 -3.07176200 -0.28085500

H 3.07224600 -3.44226400 -0.46384000

H 2.39093100 -3.13549900 1.13184900

C 4.01978500 -0.93181800 -0.02529100

H 4.40185000 -1.11633400 -1.03922800

H 4.09623500 0.13137900 0.19814400

H 4.63315200 -1.52793200 0.65415100

**Im-c’**

Pd -0.88103900 0.29816500 -0.09135800

Br 2.73210100 -1.84853800 0.07641200

Br -3.14918700 -0.63409200 0.01992500

H 2.10274200 -0.56137400 -0.13339200

O 1.11629800 1.01999800 -0.20024100

C 1.61949300 2.14344800 -0.02623300

H -1.13008300 0.83014400 1.28114900

C 0.81255600 3.30833400 0.42660000

H -0.10082700 2.98825400 0.92754800

H 1.40816400 3.96423800 1.06490700

H 0.54151700 3.88404400 -0.46796400

C 3.07595900 2.33506300 -0.28146900

H 3.58313700 2.37098900 0.69068900

H 3.49104700 1.52186500 -0.87521500

H 3.25167100 3.29725000 -0.76850500

**HPdBr**

Pd 0.01801600 -1.05239800 0.00000000

Br 0.01801600 1.40829900 0.00000000

H -1.45927700 -0.88017000 0.00000000

**Im1’**

C 5.56802300 -0.20868200 -1.31855500

C 5.02917400 -1.31739700 -0.66789300

C 3.80233300 -1.21964100 -0.01247800

C 3.08810300 -0.01218900 -0.00826300

C 3.64032800 1.09805700 -0.66924000

C 4.86811000 1.00034500 -1.31513300

H 6.52556300 -0.28341700 -1.82193900

H 5.56180800 -2.26196000 -0.66995300

H 3.39699000 -2.09797600 0.47596600

H 3.10176200 2.03751500 -0.66564800

H 5.28360100 1.86974400 -1.81286700

C 1.76324400 0.08937700 0.65337700

C 1.40862600 -0.81140100 1.82175600

H 2.29453000 -1.34704100 2.16506500

H 1.09698900 -0.17852700 2.66105700

H -0.27495000 0.91674600 1.79364800

N 0.94446100 0.96631400 0.19350700

N -0.33334900 1.03950800 0.77863400

H -1.84271000 -2.43177100 -0.02210700

C 0.32533900 -1.83603300 1.53444900

H 0.64314200 -2.69771100 0.95454000

Pd -1.34270500 -1.03146200 0.27693500

Br -2.83850900 -0.48916600 -1.68577400

C -0.85353500 -1.89724100 2.25028100

H -1.07030500 -1.14961900 3.00916000

H -1.43598600 -2.80937000 2.29786600

C -0.96900900 2.39441400 0.57082400

C -1.00752300 2.73744100 -0.91849000

H -0.00164800 2.86688500 -1.32071700

H -1.51403900 1.95518500 -1.48373300

H -1.55459400 3.67459100 -1.05319100

C -2.38516700 2.28964100 1.14495800

H -2.87181200 3.26724600 1.10525500

H -2.98636300 1.57985400 0.57368900

H -2.36450000 1.96839800 2.19189500

C -0.15797200 3.44527600 1.34508500

H -0.60624900 4.43420000 1.21865800

H -0.13897400 3.21540700 2.41588600

H 0.87180300 3.48713400 0.98149200

**TS1’**

C -5.94024300 -0.04099200 0.99385900

C -5.66156200 -0.64577100 -0.23075700

C -4.35708700 -0.66656500 -0.72339900

C -3.31245800 -0.06914300 -0.00343800

C -3.60503300 0.53899600 1.22881400

C -4.90527500 0.54951900 1.72286900

H -6.95356100 -0.03209800 1.37982200

H -6.45900300 -1.10256000 -0.80640200

H -4.16382000 -1.13249900 -1.68257900

H -2.80250600 0.99193000 1.79779000

H -5.11172200 1.01362100 2.68112500

C -1.92112100 -0.05818800 -0.52796200

C -1.38594200 -1.26089500 -1.28817800

H -2.21072000 -1.91504100 -1.57779900

H -0.86622700 -0.97414400 -2.20735300

H 0.17214800 0.74670800 -1.64749200

N -1.19409100 0.94373700 -0.19915800

N 0.15088100 0.97248500 -0.64867300

H 1.90584000 -2.41792300 -0.06996200

C -0.46774000 -2.02944300 -0.35700100

H -0.88930700 -2.19658500 0.63369600

Pd 1.34104200 -0.95928500 -0.04380700

Br 3.67404900 -0.20582600 0.78514600

C 0.53842700 -2.92678900 -0.76791300

H 0.78897200 -3.01449000 -1.82144100

H 0.71634800 -3.82446400 -0.18271800

C 0.70993600 2.37417200 -0.51768700

C 0.97374100 2.65049300 0.96245200

H 0.04920600 2.57045200 1.53971900

H 1.70332700 1.94227100 1.36041800

H 1.36738200 3.66292500 1.08673100

C 2.00525600 2.40854900 -1.33404300

H 2.45189300 3.40331800 -1.26246300

H 2.72921300 1.68137700 -0.96788400

H 1.80367500 2.20856400 -2.39244800

C -0.27957600 3.39749600 -1.09868100

H 0.18112600 4.38889100 -1.09618100

H -0.53725000 3.14556900 -2.13274300

H -1.20013300 3.44299600 -0.51560100

**Im2’**

C 5.59789600 -1.31890900 -0.84146200

C 4.65468100 -2.25030300 -0.40873500

C 3.41486500 -1.82517600 0.06509200

C 3.09351700 -0.46006400 0.09790500

C 4.05075500 0.46948200 -0.34047900

C 5.29169300 0.04313900 -0.80297100

H 6.56534300 -1.65029800 -1.20219100

H 4.88195700 -3.31012200 -0.43945500

H 2.69198700 -2.56392500 0.38978200

H 3.81724900 1.52644300 -0.30708300

H 6.02341100 0.77377800 -1.12980800

C 1.75688200 -0.00506800 0.55502500

C 0.95357200 -0.80499400 1.54869400

H 1.55462300 -1.62749600 1.94409500

H 0.72074200 -0.16645800 2.41085900

H 0.01517900 1.51877900 1.52343600

N 1.31040800 1.08833100 0.04905600

N 0.01513700 1.48212200 0.49944000

H -2.06031800 -2.53811500 1.71223000

C -0.35348300 -1.40136400 0.99792500

H -0.13800600 -2.10729600 0.19074400

Pd -1.45106500 -0.00251900 0.00711300

Br -3.24757400 -1.53940000 -0.83215200

C -1.16680400 -2.05286900 2.10313100

H -1.46784300 -1.32643100 2.86439400

H -0.55447300 -2.81640100 2.60534300

C -0.33165500 2.87708200 0.01847300

C -0.43375100 2.86708700 -1.50751300

H 0.51899800 2.59277000 -1.96355000

H -1.19513100 2.15658200 -1.84370600

H -0.71403600 3.86256600 -1.86034200

C -1.68300800 3.22659100 0.65172400

H -2.47241300 2.54963900 0.30957900

H -1.63423200 3.17460300 1.74394900

H -1.96414400 4.24473000 0.37334800

C 0.74297400 3.86253400 0.49528800

H 0.46302800 4.87865100 0.20638700

H 0.84113900 3.83360200 1.58529700

H 1.71340600 3.63306300 0.05279700

**i-PrOH**

H -0.92905900 1.75513500 0.10335200

O -0.08246000 1.37605800 -0.16476500

C 0.00252100 0.03577100 0.36512600

H -0.00902700 0.08913700 1.46294200

C 1.34071300 -0.52355300 -0.08970400

H 2.16127100 0.11157500 0.25452200

H 1.49056200 -1.52932600 0.31200400

H 1.37843600 -0.57721100 -1.18237900

C -1.17747100 -0.80982500 -0.10359400

H -1.18440900 -0.88192200 -1.19576800

H -1.11618700 -1.82021700 0.31222100

H -2.12647700 -0.36999200 0.22025400

**Acetone·HBr**

Br 1.92906200 -0.02436000 0.00065100

H 0.46704400 -0.38910200 -0.00738200

O -1.05368200 -0.86264200 -0.00327300

C -1.99171200 -0.06546500 -0.00448400

C -3.40540800 -0.56031500 0.00683000

H -3.44397100 -1.63137000 0.20257600

H -3.99312200 -0.01051200 0.74756200

H -3.85095800 -0.35015300 -0.97245300

C -1.77702700 1.41889200 -0.00602600

H -1.95900600 1.78407800 1.01184300

H -0.76102900 1.68053100 -0.29852000

H -2.50179900 1.91160900 -0.65814100
